# Discovery of a novel bacterial class with the capacity to drive sulfur cycling and microbiome structure in a paleo-ocean analog

**DOI:** 10.1038/s43705-023-00287-9

**Published:** 2023-08-18

**Authors:** Adrien Vigneron, Warwick F. Vincent, Connie Lovejoy

**Affiliations:** 1 Département de Biologie, Université Laval, Québec, QC Canada; 2 Centre d’études nordiques (CEN), Université Laval, Québec, QC Canada; 3 Institut de Biologie Intégrative et des Systèmes, Université Laval, Québec, QC Canada; 4 Takuvik Joint International Laboratory, Université Laval, Québec, QC Canada; 5 Québec Océan, Université Laval, Québec, QC Canada

**Keywords:** Limnology, Microbial ecology, Biodiversity

## Abstract

Uncultivated microbial taxa represent a large fraction of global microbial diversity and likely drive numerous biogeochemical transformations in natural ecosystems. Geographically isolated, polar ecosystems are complex microbial biomes and refuges of underexplored taxonomic and functional biodiversity. Combining amplicon sequencing with genome-centric metagenomic analysis of samples from one of the world’s northernmost lakes (Lake A, Ellesmere Island, Canadian High Arctic), we identified a novel bacterial taxon that dominates in the bottom layer of anoxic, sulfidic, relict sea water that was isolated from the Arctic Ocean some 3000 years ago. Based on phylogenomic comparative analyses, we propose that these bacteria represent a new Class within the poorly described Electryoneota/AABM5-125-24 candidate phylum. This novel class, for which we propose the name Tariuqbacteria, may be either a relict of ancient ocean conditions or endemic to this High Arctic system, provisionally providing a rare example of high-taxonomy level endemism. Consistent with the geochemistry of the bottom water, the genetic composition of the *Candidatus* Tariuqbacter genome revealed a strictly anaerobic lifestyle with the potential for sulfate and sulfur reduction, a versatile carbon metabolism and the capability to eliminate competing bacteria through methylarsenite production, suggesting an allelochemical influence on microbiome structure by this planktonic microbe.

## Introduction

Advances in DNA sequencing and computational approaches have accelerated the reconstruction of metagenome assembled genomes (MAGs) of uncultured microbial populations in multiple ecosystems [1]. This approach has revealed many novel lineages and expanded our vision of the tree of life and the ecological roles of uncultured microorganisms [2–5]. For example, the guild of potential sulfate and sulfite reducers has significantly expanded with genome-centric metagenomics [6]. As a result, the taxonomic diversity of potential sulfate-reducers now extends far away from taxa of culturable sulfate reducers to include numerous poorly characterized lineages such as *Schekmanbacteria*, *Rokubacteria*, *Zixibacteria*, and *Abyssubacteria* [6, 7]. Although these genomic results require physiological confirmation, they illustrate the power of genome-centric metagenomics to associate uncultured lineages with potential biogeochemical reactions and the need for revision of biogeochemical process models based on microbial community composition. Despite these significant advances, however, diversity surveys have suggested that many taxa remain underexplored and uncharacterized, including sulfate/sulfite reducers [8, 9].

With the contribution of metagenome assembled genomes (MAGs) the total numbers of partial and complete genomes in databases are growing exponentially with up to 317,000 and 907,000 curated genomes available in the Genome Taxonomy Database (GTDB; release 207) [10] and proGenomes v.3 database [11] respectively. Furthermore, around 1.5 million genomes are now deposited in NCBI (January 2023), making it possible to carry out extensive genomic comparisons, deep phylogenomic analysis, mapping of global distributions and exploration of community assembly [1, 12].

Extreme environments, such as hydrothermal vents [13–15], hypersaline basins [16], deep underground aquifers [3] and polar lakes [17] are major sources of previously unknown microbial biodiversity and are useful systems to investigate limits of life, microbial biogeography and the distribution of lineages in nature [18]. High Arctic Lake A (latitude 83 °N) is one of the northernmost lakes of the world and has many extreme features. Located at the far northern coast of Canada, Lake A is a perennially ice-covered, highly stratified, meromictic lake, with a bottom saline layer derived from Arctic Ocean seawater that was trapped by isostatic uplift around 3000 years ago [19]. Water column mixing is inhibited by the perennial ice cover over the lake, and by a surface 14-m thick layer of low-salinity water derived from snow and ice melt. This stratification ensures the isolation of the relict oceanic water that extends from 24 m to the bottom of the lake (128 m) and that is fully anoxic and sulfidic [19]. Microbial community composition along the steep geochemical gradients of Lake A has been previously reported, and provided insights into aquatic biogeochemical cycles across a broad range of light, salinity, oxygen and sulfur conditions [7, 20, 21]. These studies revealed a vertical succession of freshwater, marine and deep-sea anoxic microbiomes, centered on sulfur transformations and biomass recycling down the water column [7, 20, 22].

In our previous work on Lake A, we detected an intriguing microbial community in the bottom waters of the lake dominated by a large fraction of unclassified microbial lineages. The unclassified lineages from 65 m largely predominated over other microbial taxa at that depth, including *Dehalococcoidia*, *Omnitrophicaeota*, *Atribacteria* and *Desulfobacterota*, which are typically recovered from deep sea and marine sediments [7]. In the present study, we combined a new analysis of 16 S rRNA (cDNA) and rRNA gene (DNA) sequence data, with genome-centric metagenomics with a specific focus on these unknown lineages. This allowed us to recover and characterize a novel bacterial Class-level lineage. The lineage was unique to the system and appears to be a major contributor to sulfur cycling in these relict Arctic Ocean waters.

## Methods

### Site description and sample collection

Lake A is located on the northern coast of Ellesmere Island, Nunavut, in the Canadian High Arctic (latitude 83° 00’ N, longitude 75° 27’ W). The lake has an oxygenated freshwater layer (14.1 mg L^−1^ of oxygen; salinity <0.7 PSU) that extends from under the ice down to 12 m depth, a chemo- and halocline located below the freshwater down to 24 m, and an anoxic marine-derived saline deeper layer with oxygen below the detection limit of the oxygen profiler and salinity >30 PSU. Sulfate rich waters (>18 mM) are found below 24 m and sulfide concentration increase with depth (~0.5 mM at 65 m). Water samples were collected in summer 2017 from three separate 24 cm-diameter holes drilled through the ice in the center of the lake as previously detailed [7]. One liter of water was collected at eight different depths (2 m, 6 m, 14 m, 22 m, 34 m, 40 m, 55 m and 65 m) from the three holes. These depths span the oxygen, salinity and temperature gradients of the water column [7]. The triplicate water samples were filtered through separate 0.22 µm pore size Sterivex filters^TM^ (Merck Millipore Ltd., Oakville, Canada) that were then stored below −50 °C until nucleic acid extraction.

### Nucleic acid extraction

Nucleic acids (DNA and RNA) were co-extracted from two of the replicates per depth using Qiagen Allprep DNA/RNA Mini Kit with modifications (Qiagen, Hilden, Germany) [23]. The DNA extracts were stored at −20 °C until library preparation. For RNA extracts, two additional DNase steps (DNase I, Ambion, Foster City, CA, USA) were carried out to remove any trace of carried over DNA. The absence of DNA contamination was confirmed by amplification of 16 S rRNA genes with bacterial primers using the RNA extracts (undiluted and diluted ten times) as template, with no product detected after 35 PCR cycles. The RNA was then immediately converted to cDNA using a High-Capacity cDNA Reverse Transcription kit (Applied Biosystems, Foster City, CA, USA) and stored as cDNA at −20 °C until library preparation.

### PCR amplification, sequencing and analysis

Microbial community composition of the samples was determined by high throughput sequencing of bacterial 16 S rRNA (cDNA) and 16 S rRNA genes (DNA) using primers targeting the bacterial V4-V5 region (S-D-Bact-0516-a-S-18/S-D-Bact-0907-a-A-20; 460 bp product). Amplifications were carried out in duplicate using Brilliant III master Mix (Agilent Technologies, Santa Clara, CA, USA), 500 nM of each primer and 1 ng of DNA/cDNA template on 25 µl reaction volume. PCR conditions were as follows: initial denaturation at 95 °C for 2 min, then 30 cycles of denaturation at 95 °C for 15 s, annealing at 58 °C for 30 s and extension at 72 °C for 30 s; and a final extension at 72 °C for 7 min. Duplicate PCR products were pooled and purified on agarose gels using the QIAquick Gel Extraction Kit (QIAGEN), then Illumina MiSeq adaptors and sample barcodes were added during a second 10 cycles-PCR step as detailed in [24]. Samples were pooled equimolarly, then sequenced along with negative controls for nucleic acids extraction, transcription and PCRs steps, using an Illumina MiSeq v3 kit at the IBIS/Laval University, Plate-forme d’Analyses Génomiques (Québec, QC). Reads were assembled into single paired-end sequences using FLASH v2.2.0, curated and clustered into OTUs (97% sequence similarity) using VSEARCH v2.3.4. as detailed in a GitHub repository (https://github.com/CruaudPe/MiSeq_Multigenique). Taxonomic affiliations of the reads were determined with mothur [25] using both the mothur version of the Bayesian classifier and BLAST against Silva database release 132 and 138 [26]. For the BLAST algorithm, the 5 best hits of BLASTn for each sequence were collected and taxonomic affiliation was defined by the lowest classification shared by the 5 hits. In addition, the sequences of unclassified bacterial OTUs were also compared against NCBI-nr (access date: Nov. 2022), RDP (Release 11.5, taxonomy 18), IMG-16S (access date: Nov. 2022), EzBioCloud (Release 2021-07) and Greengenes v.13.5 databases.

### Metagenomic library preparation, sequencing and analysis

One metagenomic library per sampled depth was prepared using Illumina Nextera XT kit and sequenced in two Illumina MiSeq (2*300 bp) runs and one Illumina NexSeq run (2*150 bp) at the Institut de Biologie Integrative et des Systèmes (IBIS) sequencing platform (Université Laval, Canada) and at the CGEB – Integrated Microbiome Resource (Dalhousie University, Canada), respectively. Datasets were quality filtered using Trimmomatic v.0.39 [27] keeping both R1 and R2 reads when reads overlapped. Reads of ribosomal small subunit (rRNA) were extracted from metagenomic reads using Infernal v.1.1.4 [28], and taxonomic affiliation of the extracted 16 S rRNA reads longer than 100 bp were determined using BLAST against the Silva database release 138 as reference [26]. Full length 16 S rRNA genes were reconstructed from extracted 16 S rRNA reads using SPADES v.3.15.4 [29] (careful mode) and metagenomic reads were mapped against the complete 16 S rRNA genes using BBMAP v.38.86 (perfectmode option) [30] to determine depth distribution of 16 S rRNA genes. For metagenome assembled genome reconstruction, all quality filtered sequences were pooled and co-assembled using MEGAHIT v.1.2.9 [31]. Read coverage of the contigs was carried out using bwa-mem (http://bio-bwa.sourceforge.net), followed by contig binning using MetaBAT-2 [32] with contigs longer than 2000 bp. The completeness and contamination level of the MAGs were evaluated using CheckM v.1.1.5 [33]. Relative abundance of the MAGs was estimated with the average coverage of the contigs determined using bwa-mem and averaged using the jgi_summarize_bam_contig_depths script. Taxonomic affiliation of the MAGs was carried out using GTDB-tk v.2.2.6 [34]. Genomes taxonomically close to our bin of interest (bin 685) on the phylogenomic tree were downloaded and investigated for the presence of the dsr operon as previously described [6]. Amino acid identity percentage between bin 685 and these genomes was determined using AAI calculator [35]. The genetic composition of genomic bins was explored using KofamScan with the KEGG database v103.0 [36] and METABOLIC v.4 [37]. The results were manually checked for the presence of specific pathways.

For phylogenetic analysis of dsrA gene, amino acid sequences of dsrA were recovered from the 65 m metagenome and only sequences longer than 260 AA were analysed. Recovered amino acid sequences were compared against public databases (NCBI and IMG/MR) using BLASTp. Sequences with the best BLAST hits were downloaded and aligned with the metagenomic sequences using MAFFT v.7.471 [38]. Maximum likelihood tree of 180 sequences with 491 amino acid sites was constructed using IQTree v.2 with 1000 bootstraps and the LG + I + G4 model [39] and visualized using iTOL v.6 [40]. A similar methodology was used for the phylogenetic analysis of the *arsM* gene of *Candidatus* Tariuqbacter. The sequence was compared against NCBI amino-acid sequence database using BLASTp and aligned with the best hits of BLAST using MAFFT. A maximum likelihood tree of 80 sequences with 302 amino acid sites was constructed using IQTree v.2 with 1000 bootstraps and the JTT + I + G4 model, and was visualized using iTOL v.6.

## Results and discussion

### A single unclassified microorganism predominates in the bottom seawater

**Fig. 1:**
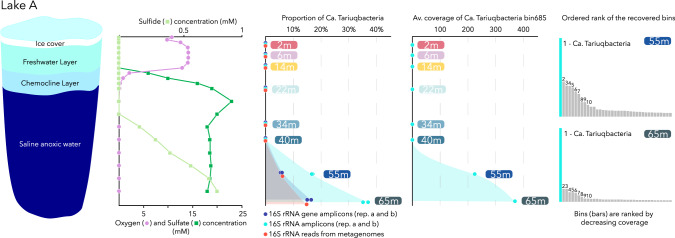
Depth distribution of *Candidatus* Tariuqbacter arcticus in the Lake A water column, based on 16 S rRNA and rRNA gene sequencing and metagenomic sequencing. Numbers in the histogram of ranked coverage refers to the following lineages: For the 55 m sample: 1 Ca. Tariuqbacteria (222 rpb), 2 Desulfaltia (99 rpb), 3 Atribacterota (88 rpb), 4 Dehalococcoidales (88 rpb), 5 Marinisomatales (83 rpb),6 Dehalococcoidales (70 rpb), 7 Marinisomatales (65 rpb),8 Aminicenantaceae (41 rpb),9 Cyanobiaceae (41 rpb),10 Dehalococcoidales (34 rpb). For the 65 m sample: 1 Ca. Tariuqbacteria (377 rpb), 2 Atribacteria (68 rpb), 3 Desulfaltia (67 rpb), 4 Dehalococcoidales (51 rpb), 5 Dehalococcoidales (51 rpb),6 Marinisomatales (48 rpb), 7 Aminicenantaceae (36 rpb),8 Ca. JABMQX01 (28 rpb),9 Desulfatibia (19 rpb),10 Bipolaricaulia (18 rpb).

The microbial survey using 16 S rRNA gene and rRNA amplicon sequencing of the Lake A water column revealed the predominance of a single OTU within bottom waters of the lake (16.5% of the 16 S rRNA genes at 65 m, Fig. 1). This single OTU, represented up to 36% of the 16 S rRNA sequences (from cDNA) amplified from the same samples, suggesting that these microorganisms were metabolically active (Fig. 1). The 16 S ribosomal sequence of the OTU was unclassified at the phylum level (Unclassified Bacteria) using both Silva (v132 and v138), RDP (Release 11.5, taxonomy 18), EzBioCloud (Release 2021-07) and GreenGenes v.13.5 databases with both RDP classifier and BLAST classification algorithms. After metagenomic sequencing of the samples, a full length 16 S rRNA gene with 100% similarity with the unclassified 16 S rRNA OTU was assembled. Mapping of the metagenomic reads sequenced from 2 m, 6 m, 14 m, 22 m, 34 m, 40 m, 55 m and 65 m samples against the novel 16 S rRNA gene sequence confirmed its absence (not-detected) in upper waters and the predominance of this lineage in the anoxic and marine bottom waters of Lake A, where it represented 15% of the 16 S rRNA metagenomic reads (Fig. 1). Comparison of the full 16 S rRNA gene sequence assembled from the metagenome dataset and the 16 S V4-V5 region amplified by PCR against the NCBI-nr database indicated that only one sequence (KM018918), amplified from the brine-seawater interface of Erba Deep brine pool samples (Red Sea), shared a percentage of identity higher than 90% over the full 16 S rRNA gene sequence (91.7%) and V4-V5 PCR-amplified region (93.3%). In addition, we then searched publicly available metagenomes through the Integrated Microbial Genomes and Microbiomes (IMG/M) portal. Only a few sequences, all recovered from the sulfidic waters of meromictic Lake Kivu (Congo, Rwanda), were found to share a percentage of identity higher than 90% (max: 91.8%) with the novel 16 S rRNA gene sequence.

**Fig. 2:**
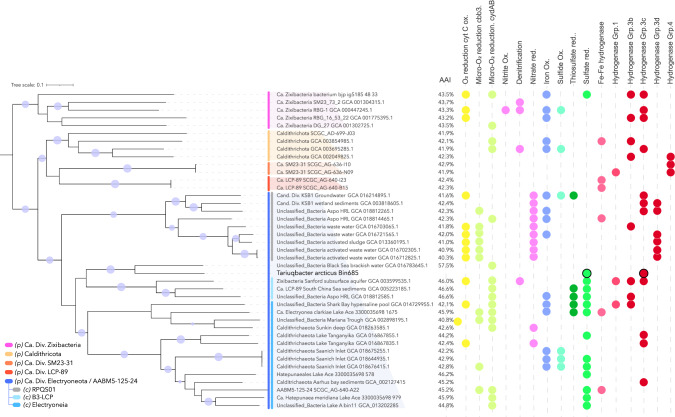
Phylogenetic tree of the Electryoneota/AABM5-125-24 phylum (blue line), including reference genomes from phylum Caldithrichota (yellow line) and candidate phylum Zixibacteria (pink line), SM23-31 (orange line), LCP-89 (red line). Only bootstraps higher than 80% are represented by purple dots. Dots indicate the detection of marker genes for specific metabolic pathways. Yellow shade: oxygen reduction, purple: nitrogen cycling, blue: iron oxidation, green: sulfur cycling and red: hydrogen metabolism. Average amino-acid identity (AAI) was calculated for each genome against the tariuqbacterial MAG.

**Fig. 3:**
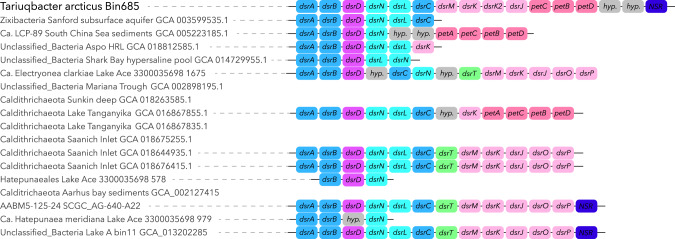
Structure of the dsr operon identified in Tariuqbacter arcticus and related genomes from the Electryoneota/AABM5-125-24 phyla. Dsr operons were represented only when at least two genes of the dsr operon including dsrA or dsrB were identified in the genomes. Representation of the operon stops where genes identified in the contigs were not related to the dsr operon.

Co-assembly and binning of the contigs from Lake A enabled the reconstruction of 250 draft metagenome-assembled genomes (MAGs), as reported previously [7, 20, 22]. The novel MAG was estimated to be 79.91% complete (contamination: 2.01%), with high coverage (up to 377 reads per base) for the 55 m and 65 m metagenomes (Fig. 1), and a 16 S rRNA gene sequence matching the unclassified rRNA gene OTU. Taxonomic affiliation of the MAG using the genome classifier tool in the proGenomes database (900,000 genomes) resulted in no match, whereas phylogenetic analyses of the MAG using GTDB-tk pipeline [34] revealed a deep branching within the Fibrobacterota, Chlorobiota and Bacteroidota (FCB) group, basal to Zixibacteria and Calditrichota phyla and within the “AABM5-125-24” phylum (Fig. 2, Supplementary Fig. 1). A few members of the AABM5-125-24 phylum, also named *Candidatus* Electryoneota [41] have been identified as rare members of Ace Lake, Antarctica [17], which is a meromictic marine-derived lake like Lake A. However, analysis of the average amino acid identity (AAI, Fig. 3) indicated a low similarity with most of Electryoneota/AABM5-125-24 genomes (<46%), including the Ace Lake representatives and a maximum of 57.5% of amino acid identity percentage with a genome recovered from Black Sea brackish waters (Fig. 2). Together these results support the classification of this MAG as a new bacterial class, order, family, genus and species within the Electryoneota/AABM5-125-24 phylum. We propose that this candidate taxon be named *Candidatus* Tariuqbacter arcticus, family Tariuqbacteraceae, order Tariuqbacterales and class Tariuqbacteria with “Tariuq” meaning saltwater in Inuktitut, the language of the Inuit, including in the community of Aujuittuq (Grise Fiord, Nunavut, Canada), the nearest human settlement to Lake A.

The low sequence similarity with reference genomes from all investigated databases suggests that Tariuqbacteria represented by *Tariuqbacter arcticus* may potentially be endemic to High Arctic Lake A and similar relict Arctic Ocean seawater habitats (e.g., lakes B, C1 and C2 in the same region [42]) that remain to be explored. The inferred endemicity of a lineage is based on the molecular markers that were used, and strongly depends on the reference databases and their taxonomic resolution [43]. Reported microbial endemism often refers to genotypes and sub-species [44–46], therefore this lineage would represent a rare example of bacterial endemism at a high taxonomic level [43]. Similar results have been previously reported in isolates of geothermal samples from Antarctica, where a single unclassified archaeon predominated the microbial community [18], suggesting that habitats in polar regions are refuges for ancient lineages that have evolved in isolation, resulting in novel biodiversity and potentially endemic populations with unusual metabolic features. However, perennially ice-covered polar lakes are largely under-sampled and little studied compared to non-polar systems, which could explain the absence of taxonomically close species in databases. In this context, the addition of the *Tariuqbacter* genome to public databases may enhance the detection of related Tariuqbacteria in future environmental studies, as for other recently described novel lineages [4].

### *Tariuqbacter* drives the sulfur cycle in relict Arctic Ocean water

The predominance of the highly abundant population of *Tariuqbacter* in Lake A suggests that members of this taxon have outcompeted other microbes and are uniquely adapted to the extreme biogeochemical conditions in the lower water column of Lake A. To decipher the underlying evolutionary and ecological forces driving this limited distribution, predicted proteins encoded in the tariuqbacterial MAG were compared against the Kegg database (release 103.0) (Supplementary Table 1) and using the METABOLIC v.4 pipeline [37], which uses numerous curated HMM profiles. A complete sulfate reduction pathway was identified, including genes coding sulfate adenyltransferase (*sat*), adenylsulfate reductase (*aprAB*) and the QmoABC membrane complex that provides AprAB electrons, and dissimilatory sulfite reductase (*dsrABC*) (Fig. 3). This capability to reduce sulfate is particularly relevant under the environmental conditions near the bottom of the lake, since sulfate concentrations of around 18 mM and higher have been measured in these fully anoxic waters (Fig. 1) [19]. Analysis of the genes identified in the vicinity of *dsrABC* revealed the presence of a complex dsr operon in *Tariuqbacter* (Fig. 3). Two divergent copies of *dsrK* gene, coding for the trisulfide reductase that act on DsrC and release hydrogen sulfide from the sulfate reduction pathway [47] were detected. Genes of periplasmic DsrO and membrane protein DsrP involved in electron transfer in the dsrKMOP complex [6] were not identified using Kegg nor METABOLIC dsr operon HMM profiles. Instead, petBCD genes coding for Rieske proteins and the cytochrome b_6_f complex were detected and specific domains of these proteins were confirmed by sequence analysis using Interpro [48]. These results suggest that electron transfer to F-type ATPase could be mediated by the cytochrome b_6_f complex. Genes coding the heterodisulfide reductase-[NiFe]-hydrogenase (HdrABC-MvhAGD) frequently found in sulfate-reducing bacteria and methanogens were also identified [49]. This complex reduces disulfide with the reduction of ferredoxin in an energy-conserving flavin-based electron bifurcation process [50]. In addition, the dsr operon in *Tariuqbacter* also included a NAD(P)H elemental sulfur oxidoreductase (NSR) suggesting that it could also reduce S_0_ (Fig. 3) [51]. A sulfidic smell and the yellow-orange color of the water below 22 m was noted during sampling, indicating the presence of polysulfides and aqueous elemental sulfur in the water, and supporting the S_0_-reducing potential of *Tariuqbacter*. Mining of the 28 publicly available genomes affiliated to “Electryoneota/AABM5-125-24” phylum failed to detect the sulfate-reducing potential in the taxonomically closest genome (57.5% AAI). However, the analysis identified 12 MAGs with the dsr operon, including one (bin11; Fig. 2) that was also recovered from Lake A deep waters, supporting the sulfate reduction potential of some Electryoneota lineages [5] (Figs. 2 and 3). However, the structure of the operon and more particularly the genes involved in electron transfer were variable, and included, in two other MAGs, genes coding the cytochrome b_6_f instead of dsrJOP. In addition to the potential for sulfate reduction, previous studies on Electryoneota lineages have reported a potential capability for aerobic respiration using high affinity cytochromes (cbb3-type and cydAB) and sulfide oxidation genes, suggesting a facultative anaerobic lifestyle [17]. Analysis of energetic pathways found in publicly available draft genomes of Electryoneota revealed that genes for aerobic respiration at low oxygen levels are frequent in the phylum (75% of the MAGs). However, none of these aerobic pathways were detected in the *Tariuqbacter* genome, suggesting a strictly anaerobic metabolism that differs from Antarctic and other Electryoneota lineages. This result is consistent with the detection of *Tariuqbacter* only in the deeper anoxic water of Lake A, while Electryoneota populations were detected in the oxic-anoxic interface of Ace Lake. The *Tariuqbacter* MAG also differed from other Electryoneota genomes by the apparent absence of nitrate and thiosulfate reduction or iron and sulfide oxidation pathways that were identified in the RPQS01 class and in other Electryoneota genomes (Fig. 2). The unique energetic pathway gene combination of *Tariuqbacter* supports its position as a novel class within the Electryoneota phylum.

**Fig. 4:**
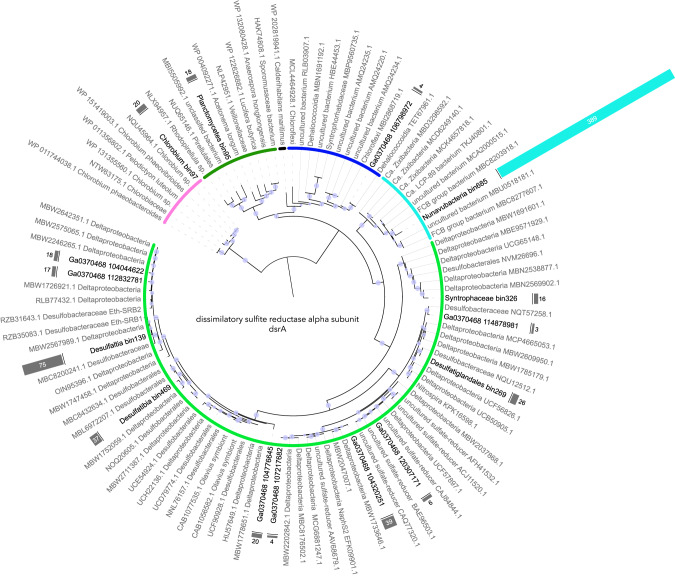
Taxonomic tree of the alpha subunit of the dissimilatory sulfite reductase (dsrA) gene (sequences longer than 250 amino-acids) recovered from the metagenome of the 65 m sample. Bars in front of the labels indicate the estimated number of copies of the sequence, indicated in or next to the bars.

Taxonomic analysis of the drsA gene recovered from the metagenomic dataset of the 65 m sample confirmed the taxonomic placement of Tariuqbacteria within the FCB group as well as the strong predominance of this lineage in the deep water of Lake A (Fig. 4). Up to 389 copies of the *Tariuqbacter* dsrA gene were identified at 65 m, representing 69% of all dsrA genes identified at this depth whereas the second most abundant dsrA gene, related to *Desulfaltia*, (MAG number 3 in Fig. 1 – 65 m) had 75 copies (Fig. 4). These results suggest that *Tariuqbacter* plays a major role in the sulfur cycle in the relict Arctic Ocean water of Lake A. Utilisation of intermediates of the sulfur cycle and sulfides has been proposed to shape a large fraction of the Lake A microbiome throughout the water column [7], and therefore *Tariuqbacter arcticus* may be a major structuring species for this extreme ecosystem.

### Metabolic versatility of *Tariuqbacter arcticus*

**Fig. 5:**
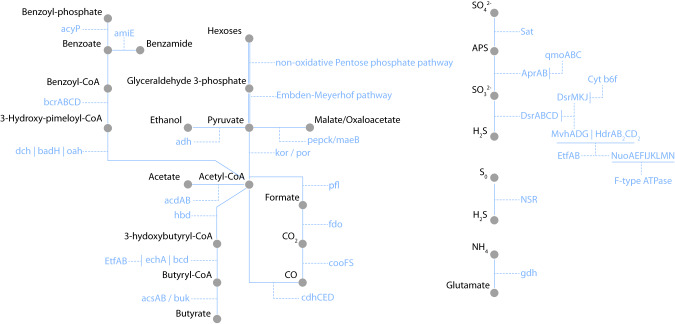
Metabolic reconstruction of *Tariuqbacter arcticus* based on genes identified using the Kegg database (release 103.0).

The potential for degradation of hexoses to pyruvate through the non-oxidative pentose phosphate pathway and the Embden-Meyerhof pathway was detected in the *Tariuqbacter* MAG (Fig. 5), suggesting a sugar-based metabolism. The TCA cycle was incomplete in the MAG. However, an alcohol dehydrogenase gene was identified providing the Lake A *Tariuqbacter* with a pathway for pyruvate fermentation. Genes coding for pyruvate ferredoxin oxidoreductase (por) and 2-oxoglutarate ferredoxin oxidoreductase (kor), converting pyruvate into acetyl-CoA were detected as well as a gene coding for the acetyl-CoA synthetase (ADP-forming) allowing acetate production and ATP generation from acetyl-CoA. This feature, usually found in fermentative Archaea, has also been observed in Zixibacteria a taxonomically close phyla of Electryoneota, where it was proposed to catalyse the reverse reaction, enabling acetate utilisation [52]. Genes coding for pyruvate formate lyase and activating enzymes (plfACEX) were also detected, suggesting a potential for formate utilisation [53]. Formate could also be converted to acetyl-CoA through CO_2_ and CO by formate dehydrogenase (fdo), anaerobic carbon-monoxide dehydrogenase (cooFS) and the acetyl-CoA decarbonylase/synthase complex (cdhCED) (Fig. 5). Formate is a volatile fatty acid frequently associated with metabolic exchanges and electron transfer, notably under anaerobic conditions [54, 55]. These results suggest that *Tariuqbacter* could be involved in cooperative interspecies interactions.

A pathway for the anaerobic degradation of aromatic compounds (benzoate and aminobenzoate) to acetyl-CoA was identified (Fig. 5). Genome analysis also suggested the capability of butyrate-fermentation in Tariuqbacteria, as reported in Electryoneia and Zixibacteria [56]. In this pathway, butyryl-CoA is converted from acetyl-CoA by four enzymes (Hbd, Crt, Ech and Bcd) and the electron transfer flavoprotein complex (Etf). The butyryl-CoA dehydrogenase-EtfAB complex provide another cytoplasmic flavin-based electron bifurcation mechanism for energy conservation and hydrogen production in *Tariuqbacter* [50]. Butyryl-CoA is then converted to butyrate by butyrate kinase (Buk) or acetyl-CoA synthetase (Acs) resulting in the generation of ATP by substrate-level phosphorylation (Fig. 5) [52, 56]. All of these processes can be reversible under certain conditions and may thus serve in alcohol, acetate, carbon monoxide and butyrate utilization, indicating the versatility of anaerobic metabolism in *Tariuqbacter*, and potentially explaining its predominance in Lake A. In addition, autotrophic abilities through reverse TCA cycle have been previously proposed for Electryoneota lineages recovered from Ace Lake. We did not detect genes coding acetate citrate lyase, fumarate reductase and citryl-CoA synthetase or lyase (*aclAB*, *frdABCDE*, *ccsAB* and *ccl*, respectively), nor did we detect other CO_2_ fixation pathways. Although we can not exclude the possibility that key genes were not detected due to the incompleteness of the MAG, the lack of CO_2_ fixation pathways suggests that *Tariuqbacter* has a strictly heterotrophic lifestyle.

### Toxic success of *Tariuqbacter*?

**Fig. 6:**
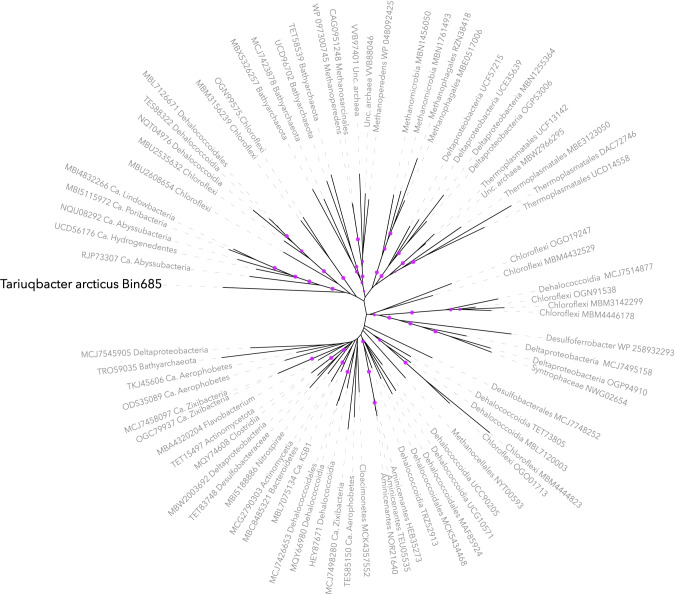
Unrooted taxonomic tree of closest representatives for the arsenite S-adenomethyltransferase (ArsM) gene recovered from the *Tariuqbacteria* MAG.

Heavy metals such as mercury and arsenic occur naturally in the environment and are also conveyed from anthropogenic sources to the Arctic region by atmospheric circulation [57], leading to a bioaccumulation of these pollutants in Arctic aquatic ecosystems. For example, 0.1 µg of Hg per gram of sediment was detected in Lake A [58], supporting the presence of heavy metals in the ecosystem. Arsenic has never been quantified in Lake A. However, 118 MAGs over the 250 good-quality MAGs (44.8%) recovered from Lake A samples from all depths included arsenic resistance and detoxification systems, such as arsenate reductase and arsenite transporters [59], suggesting that arsenic is likely present in the system. The *Tariuqbacter* MAG includes arsenite transporters (ACR3/arsB) and intracellular arsenate reductase (ArsC) that catalyzes the reduction of arsenate to arsenite. Interestingly, the *Tariuqbacter* MAG also includes the arsenite S-adenomethyltransferase gene (*arsM*), conferring the capability to methylate arsenite into highly toxic trivalent methylated species such as methylarsenite and dimethylarsenite [60, 61]. Under aerobic conditions, this enzyme is considered as a major detoxification pathway since trivalent methylated species are rapidly and abiotically oxidized to non-toxic pentavalent species methylarsenate, dimethylarsenate and volatile trimethylarsine [60]. However, under anoxic conditions such as at the bottom waters of Lake A, methylarsenite and dimethylarsenite are thermodynamically stable and are highly toxic antimicrobial compounds [62]. This gene has been previously detected in a few genomes related to the sister phyla Zixibacteria as well as in other anaerobic marine lineages (Fig. 6). However, phylogenetic analysis of the arsM sequence revealed that *Tariuqbacter* gene was more related to sequences recovered form *Candidate* phylum Hydrogenedentes/Abyssubacteria bacteria (Fig. 6), illustrating the numerous horizontal transfer events that characterize this gene [60]. Analysis of the sequence confirmed the presence of a SAM-binding domain, as well as the four conserved cysteine residues allowing arsenite binding and supporting its activity. A gene coding for a methylarsenite efflux permease (*arsP*) [63], which is frequently observed in anaerobic arsenite methylators [60], was also identified in the *Tariuqbacter* MAG. This enzyme protects the producing bacteria by pumping out toxic methylarsenite, leading to the death of neighboring bacteria [62]. This system has been proposed as an important selective mechanism that potentially favored cyanobacterial blooms in the anoxic paleo-ocean [62]. Known methylarsenite detoxification pathways so far are limited to oxic and nitrate-reducing conditions [64], and were only identified at the upper oxic layer of Lake A. Therefore, in Lake A, which might be considered a geochemical analog of the anoxic paleo-ocean, this pathway could confer a significant fitness advantage for *Tariuqbacter*, potentially explaining their ecological success and their predominance in the anoxic marine waters of this ecosystem.

## Conclusions

Isolated by perennial ice-cover at the northern limit of Canada, meromictic Lake A is a compelling model to explore microbial metabolism and adaptation to environmental extremes. Our genomic analysis of the deep anoxic and sulfidic water of the lake revealed the predominance of a novel candidate bacterial taxon that likely drives the sulfur cycle of the ecosystem and that may have a controlling influence on overall microbiome structure. Culturing or activity measurements are needed to confirm the ecophysiology of Tariuqbacteria. However, our results indicate that these bacteria may outcompete other microorganisms by relying on a combination of metabolic versatility and biotoxicity. Although additional explorations of polar lakes are needed to better estimate bacterial distribution in these remote ecosystems, this lineage may potentially represent a rare example of high taxonomy level endemism outside of hydro-geothermal areas. Our observations support the hypothesis that isolated polar habitats host an unexplored microbial diversity with lineages that escape global dispersion and that may avoid competition with cosmopolitan taxa.

## Supplementary information

Supplementary Figure 1

Supplementary Material 1

Supplementary Material 2

Supplementary Material 3

## Data Availability

Assembled metagenome data are available in IMG/MR (https://img.jgi.doe.gov/mer/) under the following accession numbers: 3300033443, 3300033444, 3300033445, 3300033439, 3300033411, 3300033473, 3300033474, 3300033495. Co-assembly is also available on IMG/MR under accession number 3300033064. Raw amplicon sequences and bin files were deposited in the NCBI public database under Bioproject PRJNA616293. The Tariuqbacter MAG is available in Supplementary Material as well as in GTDB-tk and GenBank/NCBI databases under accession number GCA_013202315.1/SAMN14944631. In addition, the full length 16 S rRNA gene sequence of *Ca*. Tariuqbacter is deposited in GenBank under accession number OQ709077. The list of Kegg Orthologies identified in the Tariuqbacter MAG is available in Supplementary Material. In-house scripts used in this study are available on GitHub/CruaudPe. Environmental metadata were previously published [7, 19, 42] and additional data for Lake A limnology and local climate are available in the Nordicana D database (https://nordicana.cen.ulaval.ca/).
