# Supplementary figures and images for: Discovery of a novel bacterial class with the capacity to drive sulfur cycling and microbiome structure in a paleo-ocean analog
Source: ISME Commun. 2023 Aug 18;3:82. doi: 10.1038/s43705-023-00287-9 (PMC10439189; doi:10.1038/s43705-023-00287-9)

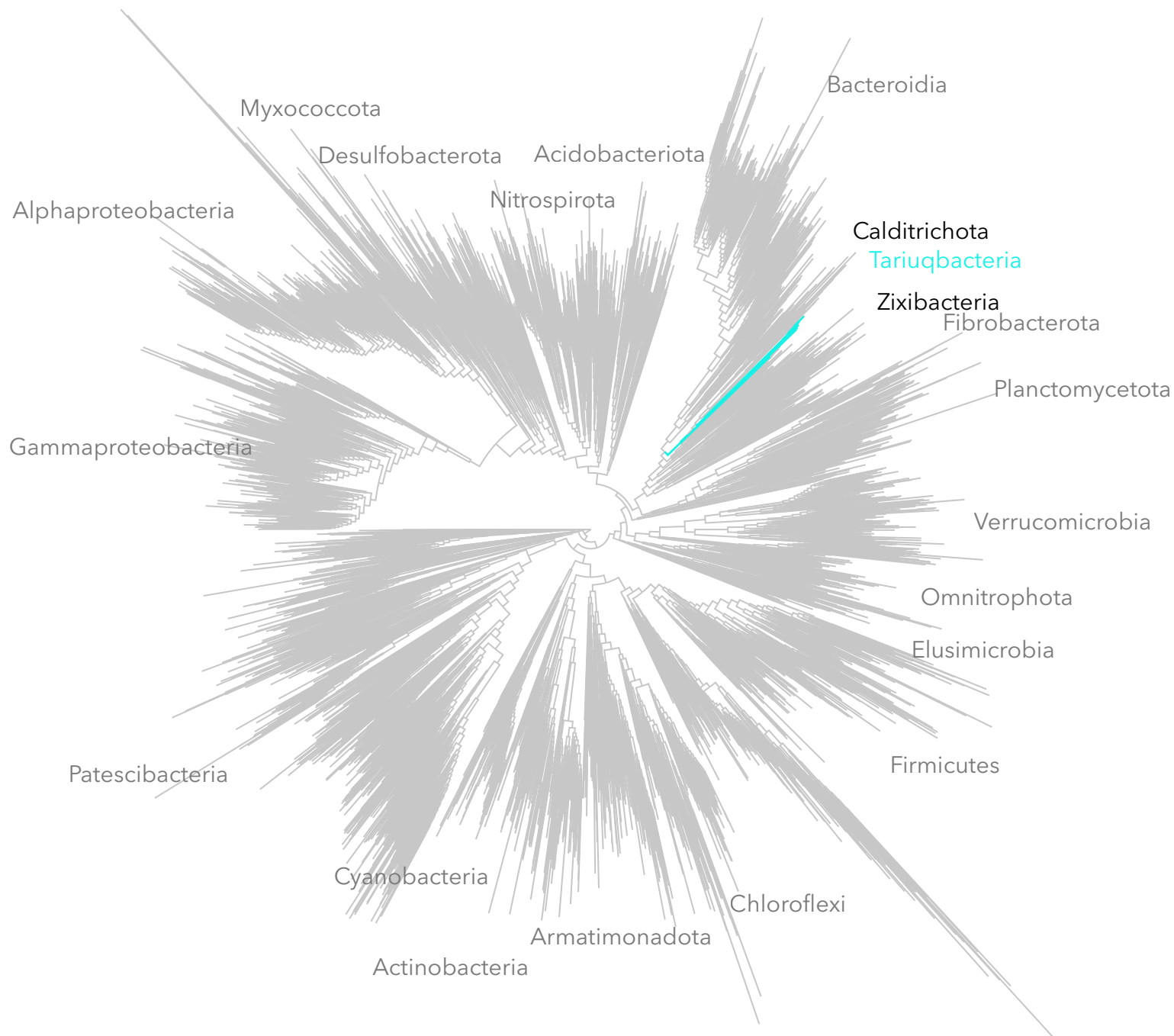

Supplement: Supplementary file 1 — Supplementary Figure 1 [file 43705_2023_287_MOESM1_ESM.pdf]
