# Supplementary material for: Discovery of a novel bacterial class with the capacity to drive sulfur cycling and microbiome structure in a paleo-ocean analog
Source: ISME Commun. 2023 Aug 18;3:82. doi: 10.1038/s43705-023-00287-9 (PMC10439189; doi:10.1038/s43705-023-00287-9)
Supplement: Supplementary file 4 — Supplementary Material 3 [file 43705_2023_287_MOESM4_ESM.pdf]

(((((GB_GCA_001829155.1:0.63259,GB_GCA_002450905.1:0.49676)'69.0:c__UBA6919':0.12072,(GB_
GCA_003645695.1:0.52194,(GB_GCA_012517545.1:0.94457,(GB_GCA_002070605.1:0.68726,((RS_G
CF_011812985.1:0.70964,((GB_GCA_016936615.1:0.47636,((GB_GCA_016936555.1:0.32804,GB_GC
A_016936295.1:0.37804)'83.0:o__Spirochaetales_E':0.05605,(RS_GCF_000426705.1:0.30887,GB_GC
A_013043995.1:0.47183)35.0:0.05755)32.0:0.03076)13.0:0.02615,((GB_GCA_002307015.1:0.27939,(
(GB_GCA_009782495.1:0.48259,GB_GCA_017614995.1:0.55001)73.0:0.04629,(GB_GCA_016926955.
1:0.28928,GB_GCA_015657395.1:0.45081)100.0:0.11267)53.0:0.03609)'100.0:o__Treponematales':0
.13181,(((GB_GCA_009991955.1:0.43949,((RS_GCF_000373545.1:0.43186,RS_GCF_000242595.2:0.3
1846)48.0:0.05622,(GB_GCA_003552745.1:0.38794,(GB_GCA_007130025.1:0.31964,(GB_GCA_0071
30265.1:0.35008,(GB_GCA_003566875.1:0.30681,GB_GCA_007126415.1:0.35223)45.0:0.02836)24.0:
0.01861)85.0:0.03791)27.0:0.02464)85.0:0.03627)'99.0:o__DSM-
27196':0.05681,(GB_GCA_011329335.1:0.29407,RS_GCF_000147075.1:0.38024)'86.0:o__Spirochaet
ales_A':0.06182)84.0:0.0405,((((GB_GCA_003645645.1:0.28083,GB_GCA_004525155.1:0.30172)'76.0
:o__SPDA01':0.05175,((GB_GCA_003641675.1:0.31841,(GB_GCA_016935715.1:0.34192,(GB_GCA_00
3154555.1:0.37054,GB_GCA_002238925.1:0.54897)83.0:0.06818)80.0:0.04628)47.0:0.0279,(GB_GC
A_003818675.1:0.49143,GB_GCA_016935735.1:0.43983)51.0:0.02697)'100.0:o__SZUA-
6':0.08507)40.0:0.02133,GB_GCA_017535435.1:0.4832)3.0:0.01441,(((GB_GCA_003454605.1:0.3666
5,GB_GCA_016935695.1:0.3466)'95.0:o__JC444':0.04219,((((GB_GCA_018399255.1:0.155362,bin.3:0
.148502):0.127838,GB_GCA_017632495.1:0.62168)69.0:0.05269,GB_GCA_003559765.1:0.37626)87.
0:0.09938,(GB_GCA_015493635.1:0.28473,RS_GCF_000143985.1:0.29074)71.0:0.03204)46.0:0.0459
6)26.0:0.0183,(GB_GCA_016930115.1:0.34043,GB_GCA_016932135.1:0.29534)77.0:0.06186)22.0:0.
03267)12.0:0.02434)27.0:0.0248)67.0:0.04331)52.0:0.05808)92.0:0.06575,(RS_GCF_000222305.1:0.
68108,GB_GCA_011524535.1:0.78009)79.0:0.08991)'100.0:c__Spirochaetia':0.1304)100.0:0.06739)7
6.0:0.04461)39.0:0.03885)55.0:0.04112,((GB_GCA_011358125.1:0.72154,(RS_GCF_900112165.1:0.5
4997,(GB_GCA_001829415.1:0.37144,GB_GCA_013177995.1:0.44539)53.0:0.06788)'100.0:o__Brevi
nematales':0.1864)'100.0:c__Brevinematia':0.14412,((GB_GCA_016214035.1:0.40897,GB_GCA_9105
88665.1:0.52092)'100.0:c__Brachyspirae;o__Brachyspirales':0.29787,(GB_GCA_003497555.1:0.5616
4,(GB_GCA_013426755.1:0.78295,GB_GCA_017302015.1:0.55276)'100.0:o__COTS27':0.15613)'100.0
:c__UBA12135':0.18945)97.0:0.07875)37.0:0.04142)97.0:0.05532,((GB_GCA_018817865.1:0.35751,G
B_GCA_016931325.1:0.32492)'100.0:c__JAHITG01;o__JAHITG01':0.35411,((GB_GCA_016934935.1:0.
441,(GB_GCA_002328585.1:0.33808,(GB_GCA_016933025.1:0.42789,(GB_GCA_016932015.1:0.3427
8,(GB_GCA_017998375.1:0.25374,GB_GCA_002069125.1:0.32229)90.0:0.03255)66.0:0.02802)65.0:0
.03436)100.0:0.06811)'100.0:c__UBA4802;o__UBA4802':0.21049,((GB_GCA_003695005.1:0.36432,G
B_GCA_017302045.1:0.49002)'100.0:o__Turneriellales':0.27933,(RS_GCF_002811765.1:0.68915,(GB
_GCA_014238825.1:0.56843,(GB_GCA_015075425.1:0.40723,GB_GCA_002730875.1:0.4565)55.0:0.0
5496)100.0:0.16982)'100.0:o__Leptospirales':0.12251)'100.0:c__Leptospirae':0.12382)100.0:0.09777
)78.0:0.03809)'96.0:p__Spirochaetota':0.07558,(GB_GCA_017999075.1:0.75991,((GB_GCA_0027800
65.1:0.3812,GB_GCA_003697085.1:0.37501)'100.0:p__UBA8481;c__UBA8481;o__UBA8481':0.35889
,(GB_GCA_001784175.1:0.70909,GB_GCA_003557545.1:0.79846)99.0:0.10132)90.0:0.05135)21.0:0.
06176,((((GB_GCA_002069765.1:0.63899,GB_GCA_009928855.1:0.76047)84.0:0.09228,((GB_GCA_0
02422125.1:0.80313,(GB_GCA_016213885.1:0.41472,GB_GCA_016213865.1:0.45051)'100.0:o__JAC
PRK01':0.23265)'100.0:p__UBP17;c__UBA6191':0.11404,GB_GCA_002869225.1:0.68505)49.0:0.0466
2)84.0:0.06402,((GB_GCA_008086245.1:0.37408,GB_GCA_016937175.1:0.34628)'100.0:p__Mcinern
eyibacteriota;c__Mcinerneyibacteria;o__Mcinerneyibacteriales':0.34382,GB_GCA_003554345.1:0.78
717)37.0:0.09747)2.0:0.03331,((((GB_GCA_002313635.1:0.70508,(RS_GCF_000024405.1:0.42142,(G
B_GCA_012519615.1:0.33137,RS_GCF_008633215.1:0.55434)99.0:0.06075)'100.0:o__Fusobacteriale
s':0.14619)'100.0:p__Fusobacteriota;c__Fusobacteriia':0.23025,((GB_GCA_011373475.1:0.91319,(GB
_GCA_011047215.1:0.83529,GB_GCA_014384545.1:1.20414)39.0:0.08727)46.0:0.09465,(((GB_GCA_

016871815.1:0.65119,(GB_GCA_003511165.1:0.41834,(GB_GCA_016699045.1:0.37738,(GB_GCA_01
8817025.1:0.34282,GB_GCA_014730005.1:0.33252)69.0:0.03815)49.0:0.03367)81.0:0.04296)100.0:0
.10422,(((((GB_GCA_016191325.1:0.34101,GB_GCA_000989525.1:0.36492)36.0:0.05449,GB_GCA_01
6929235.1:0.39573)18.0:0.03558,(((GB_GCA_018061805.1:0.63103,GB_GCA_000991575.1:0.45039)
26.0:0.03279,GB_GCA_013287265.1:0.3832)27.0:0.02931,(GB_GCA_018647505.1:0.3741,(GB_GCA_
903873255.1:0.275848,bin.331:0.291277):0.129452)15.0:0.05256)5.0:0.02263)19.0:0.02416,GB_GCA
_002441565.1:0.46051)27.0:0.02351,(GB_GCA_013821315.1:0.4165,GB_GCA_018663245.1:0.5379)5
0.0:0.03838)100.0:0.11902)'100.0:p__Dependentiae;c__Babeliae;o__Babeliales':0.5433,(((GB_GCA_0
13153685.1:0.2296,RS_GCF_000021725.1:0.2744)'100.0:o__Nautiliales':0.11056,(GB_GCA_0002769
65.1:0.43317,(RS_GCF_015100395.1:0.24322,((GB_GCA_015494675.1:0.28238,GB_GCA_018822805.
1:0.50392)86.0:0.04558,((GB_GCA_903885085.1:0.46554,(GB_GCA_011334775.1:0.38868,GB_GCA_
003250175.1:0.3251)100.0:0.06699)35.0:0.02943,(((GB_GCA_011390245.1:0.27601,RS_GCF_003364
265.1:0.63129)100.0:0.07835,(GB_GCA_002382325.1:0.3772,(GB_GCA_013388565.1:0.28785,GB_G
CA_016744535.1:0.24169)80.0:0.04535)50.0:0.02356)80.0:0.02472,(((GB_GCA_018058685.1:0.2746
7,(RS_GCF_002912895.1:0.44669,GB_GCA_014859645.1:0.25253)70.0:0.03255)82.0:0.03782,(GB_G
CA_013153915.1:0.25551,(GB_GCA_013154075.1:0.20692,GB_GCA_003978405.1:0.32136)42.0:0.02
137)61.0:0.0218)69.0:0.02477,GB_GCA_902727925.1:0.42142)90.0:0.04929)81.0:0.02642)15.0:0.023
85)11.0:0.02774)67.0:0.04724)'100.0:o__Campylobacterales':0.06571)'100.0:c__Campylobacteria':0.
45918,(GB_GCA_013152955.1:0.71098,(GB_GCA_000517565.1:0.3746,GB_GCA_015490005.1:0.476
03)'100.0:o__Desulfurellales':0.20197)'100.0:c__Desulfurellia':0.13429)'86.0:p__Campylobacterota':
0.06589)21.0:0.03853)8.0:0.03616)0.0:0.04638,(((GB_GCA_001443005.1:0.44916,(GB_GCA_0113759
65.1:0.46068,((GB_GCA_001508935.1:0.31157,((GB_GCA_017656375.1:0.40067,((GB_GCA_0169277
65.1:0.31949,GB_GCA_017454625.1:0.92134)100.0:0.05261,RS_GCF_000177335.1:0.57657)100.0:0.
058)42.0:0.02027,((GB_GCA_015657435.1:0.37355,GB_GCA_012728165.1:0.5645)65.0:0.0273,(GB_
GCA_002425685.1:0.31427,GB_GCA_018398915.1:0.31658)78.0:0.05076)98.0:0.02682)100.0:0.0516
7)56.0:0.03728,RS_GCF_000160455.2:0.35907)'100.0:c__Synergistia;o__Synergistales':0.11492)100.
0:0.1611)'100.0:p__Synergistota':0.14256,(((GB_GCA_016179105.1:0.39011,(GB_GCA_011052375.1:
0.35559,GB_GCA_018609825.1:0.44219)100.0:0.07249)'100.0:o__RBG-16-55-
9':0.13905,(GB_GCA_018334915.1:0.66196,(RS_GCF_900465355.1:0.66826,(GB_GCA_003535305.1:
0.36644,(GB_GCA_018817505.1:0.408328,bin.435:0.25863):0.121562)'100.0:o__UBA7950':0.12517)
100.0:0.08578)80.0:0.04251)'100.0:p__Bipolaricaulota;c__Bipolaricaulia':0.29046,(GB_GCA_0036415
55.1:0.53547,(GB_GCA_011357985.1:0.6166,((GB_GCA_003641795.1:0.42885,((RS_GCF_002865985.
1:0.37645,(RS_GCF_000504085.1:0.45866,RS_GCF_000016905.1:0.4866)84.0:0.03588)'100.0:o__The
rmotogales':0.08773,(GB_GCA_001603185.1:0.59446,RS_GCF_002895525.1:0.72038)'97.0:o__Petrot
ogales':0.06424)53.0:0.02995)80.0:0.03982,((GB_GCA_011043375.1:0.2639,GB_GCA_003641545.1:0
.25925)'100.0:o__B21-
G9':0.10343,GB_GCA_012744415.1:0.63654)37.0:0.03978)83.0:0.05308)100.0:0.07463)'100.0:p__Th
ermotogota;c__Thermotogae':0.17298)85.0:0.0551)24.0:0.03162,((GB_GCA_014360335.1:0.61174,(
GB_GCA_002878375.1:0.55023,GB_GCA_014360465.1:0.61808)27.0:0.05999)22.0:0.04457,(((GB_GC
A_017992555.1:0.67953,(GB_GCA_018054005.1:0.3635,GB_GCA_002428295.1:0.49716)'100.0:o__U
BA6126':0.31257)100.0:0.11195,(GB_GCA_012517755.1:0.66589,(RS_GCF_003570985.1:0.75762,GB
_GCA_001871575.1:0.60416)100.0:0.11973)77.0:0.0529)'100.0:p__Caldisericota;c__Caldisericia':0.1
0101,(RS_GCF_003057965.1:0.85572,(GB_GCA_018725485.1:0.75107,(GB_GCA_013153035.1:0.534
27,RS_GCF_000020945.1:0.6378)'100.0:p__Coprothermobacterota;c__Coprothermobacteria;o__Cop
rothermobacterales':0.23199)84.0:0.06321)25.0:0.06902)10.0:0.0461)14.0:0.04312)9.0:0.03805)0.0:
0.02966,((((((((GB_GCA_018817365.1:0.65615,(((GB_GCA_016220165.1:0.3147,GB_GCA_01693421
5.1:0.44225)'100.0:o__UM-FILTER-43-
11':0.19073,(GB_GCA_018652265.1:0.35934,(((GB_GCA_002772745.1:0.293679,bin.208:0.186936):0

.00646454,bin.620:0.166955):0.0736239,bin.686:0.246617):0.186512)'100.0:o__Peribacterales':0.28
515)67.0:0:0.05013,((GB_GCA_002401425.1:0.6639,(GB_GCA_016699755.1:0.49939,GB_GCA_903882
825.1:0.57088)'100.0:o__CAIRYL01':0.13045)61.0:0:0.06139,GB_GCA_018608565.1:0.78337)46.0:0.05
446)40.0:0:0.0294)67.0:0:0.04134,((((GB_GCA_018816885.1:0.45561,(GB_GCA_000993615.1:0.46484,G
B_GCA_016699145.1:0.5529)67.0:0:0.05141)90.0:0:0.04817,(GB_GCA_000989565.1:0.44113,GB_GCA_0
18829865.1:0.39676)94.0:0:0.09135)5.0:0:0.03269,((GB_GCA_002788775.1:0.59127,GB_GCA_01623591
5.1:0.52019)59.0:0:0.04908,(GB_GCA_903858295.1:0.58319,(GB_GCA_016215735.1:0.50025,(GB_GCA
_016928915.1:0.40804,(GB_GCA_018826445.1:0.35291,GB_GCA_018818755.1:0.42829)96.0:0:0.0434
8)37.0:0:0.02702)85.0:0:0.03114)90.0:0:0.04301)27.0:0:0.03159)41.0:0:0.04559,((GB_GCA_002788755.1:0.44
961,GB_GCA_018662325.1:0.66074)100.0:0:0.11195,(GB_GCA_018697675.1:0.46034,GB_GCA_00178
7465.1:0.46971)93.0:0:0.06945)35.0:0:0.04325)'100.0:o__UBA1369':0.06644)88.0:0:0.06144,(GB_GCA_00
2783265.1:0.70628,GB_GCA_017984655.1:0.76665)72.0:0:0.0896)'77.0:c__Gracilibacteria':0.06265,((G
B_GCA_015489035.1:1.13281,(GB_GCA_010119095.1:0.54366,(GB_GCA_017992415.1:0.72281,GB_
GCA_017993345.1:0.58714)97.0:0:0.08723)'100.0:o__BD1-
5':0.22963)'86.0:c__JAEDAM01':0.07033,GB_GCA_014376695.1:1.45529)33.0:0:0.08526)68.0:0.11775,
((((GB_GCA_018814055.1:0.6074,GB_GCA_016188915.1:0.59624)60.0:0:0.08751,(GB_GCA_001771185
.1:0.47689,GB_GCA_003524745.1:0.57321)'100.0:c__Kazan-3B-28;o__Kazan-3B-
28':0.30439)11.0:0:0.0554,((GB_GCA_009694835.1:0.71859,(GB_GCA_002792735.1:0.53641,GB_GCA_
004374725.1:0.53862)33.0:0:0.08024)1.0:0:0.0409,((GB_GCA_000993275.1:0.51439,(GB_GCA_0027853
25.1:0.42334,(GB_GCA_903842085.1:0.42148,GB_GCA_002338125.1:0.45396)76.0:0:0.0531)'99.0:o__
UBA1875':0.06928)'92.0:c__CPR2':0.08017,(((GB_GCA_004297735.1:0.30451,GB_GCA_016699105.1:
0.45137)'100.0:o__UBA4664':0.15901,(GB_GCA_007131065.1:0.49621,((GB_GCA_018057585.1:0.36
409,GB_GCA_012965045.1:0.35214)100.0:0:0.10774,((GB_GCA_018061375.1:0.36511,(GB_GCA_0168
80595.1:0.333,((GB_GCA_002861585.1:0.26399,GB_GCA_016789455.1:0.31764)59.0:0:0.03823,(GB_G
CA_009787015.1:0.4928,(GB_GCA_010014665.1:0.36117,(GB_GCA_016196195.1:0.30085,((((GB_GC
A_905371595.1:0.40309,GB_GCA_000503915.1:0.22089)55.0:0:0.02323,(GB_GCA_002331045.1:0.220
65,GB_GCA_002429245.1:0.35939)32.0:0:0.02763)21.0:0:0.02417,(GB_GCA_013333595.2:0.29014,GB_G
CA_905373795.1:0.30934)78.0:0:0.0245)76.0:0:0.04197,((GB_GCA_002363125.1:0.82562,GB_GCA_0152
56915.2:0.31895)79.0:0:0.05927,(GB_GCA_018064165.1:0.25765,GB_GCA_007845205.1:0.33226)42.0:
0.0409)36.0:0:0.03321)88.0:0:0.0448)67.0:0:0.03097)33.0:0:0.02115)99.0:0:0.05044)65.0:0:0.03526)100.0:0:0.077
4)100.0:0:0.05995,((GB_GCA_903880655.1:0.40718,(((GB_GCA_009923445.1:0.4057,(GB_GCA_01621
9345.1:0.33334,GB_GCA_007135955.1:0.381)41.0:0:0.04655)5.0:0:0.02748,((((GB_GCA_018500155.1:0.
25509,GB_GCA_903946795.1:0.2879)40.0:0:0.05179,((GB_GCA_900299395.1:0.1926,GB_GCA_001790
455.1:0.28211)37.0:0:0.04992,GB_GCA_013815785.1:0.27025)28.0:0:0.03348)10.0:0:0.01823,GB_GCA_00
2413865.1:0.37898)3.0:0:0.02732,(GB_GCA_013694995.1:0.30003,GB_GCA_001788565.1:0.46686)23.
0:0.04542)2.0:0:0.02499)7.0:0:0.0254,GB_GCA_903836325.1:0.43979)95.0:0:0.03784)81.0:0:0.04163,GB_GC
A_009839685.1:0.73353)89.0:0:0.04823)99.0:0:0.07717)'100.0:o__Saccharimonadales':0.12391)100.0:0:0.
09583)81.0:0:0.05486,(GB_GCA_017983775.1:0.79869,(GB_GCA_016700035.1:0.46518,(GB_GCA_009
691435.1:0.37111,(GB_GCA_016191105.1:0.30678,GB_GCA_903832035.1:0.35613)70.0:0:0.06397)100
.0:0:0.11964)'100.0:o__CAILAD01':0.09131)99.0:0:0.10545)'100.0:c__Saccharimonadia':0.13904)32.0:0:0.0
5253)2.0:0:0.03624)15.0:0:0.03034,((((GB_GCA_016203935.1:0.6929,(GB_GCA_016197005.1:0.68377,G
B_GCA_007376145.1:0.65166)'100.0:o__UBA10190':0.13554)'92.0:c__Andersenbacteria':0.08292,(G
B_GCA_001779955.1:0.57217,((GB_GCA_007375915.1:0.38526,GB_GCA_003151625.1:0.49329)100.
0:0.13627,(GB_GCA_016182875.1:0.56551,(GB_GCA_001779925.1:0.45963,GB_GCA_016205055.1:0
.57962)100.0:0:0.07696)55.0:0:0.04531)66.0:0:0.04626)'100.0:c__Doudnabacteria;o__UBA920':0.16932)2
6.0:0:0.03186,(((GB_GCA_002792135.1:0.60358,((GB_GCA_013202585.1:6.34186e-
06,bin.624:6.11352e-
06):0.415614,GB_GCA_018818205.1:0.51821)'100.0:c__JABMPQ01;o__JABMPQ01':0.10695)66.0:0:0.0

6573,(((((((GB_GCA_001818835.1:0.55111,GB_GCA_016784445.1:0.51987)78.0:0.0526,((GB_GCA_00
1818365.1:0.58449,GB_GCA_018664825.1:0.53327)100.0:0.09779,GB_GCA_019248485.1:0.56697)4
6.0:0.0431)'95.0:o__UBA1558':0.07461,(GB_GCA_015485655.1:0.60475,((GB_GCA_018698735.1:0.5
4808,GB_GCA_001818155.1:0.59541)100.0:0.08789,GB_GCA_016181765.1:0.74542)65.0:0.05648)27
.0:0.03316)26.0:0.0348,(GB_GCA_015485715.1:0.56648,(GB_GCA_012798565.1:0.52196,GB_GCA_0
02778535.1:0.47961)100.0:0.10843)'95.0:o__UBA2196':0.06992)30.0:0.03501,((((GB_GCA_0147290
25.1:0.44027,(GB_GCA_016212745.1:0.47343,(GB_GCA_001778235.1:0.40381,(GB_GCA_018674305
.1:0.210679,bin.190:0.167023):0.214331)99.0:0.05924)'100.0:o__UBA11705':0.12867)47.0:0.03515,(
GB_GCA_016218485.1:0.54592,((GB_GCA_002771615.1:0.47414,(((GB_GCA_903839955.1:0.51706,
GB_GCA_014728685.1:0.34614)72.0:0.04833,(((((GB_GCA_018822835.1:0.29544,bin.167:0.214016):
0.0353195,GB_GCA_003647675.1:0.30201)22.0:0.03265,((GB_GCA_003231075.1:0.19536,GB_GCA_
002781265.1:0.24084)79.0:0.04923,(GB_GCA_018896595.1:0.2192,GB_GCA_018813805.1:0.33036)7
9.0:0.02848)65.0:0.02446)10.0:0.02456,(GB_GCA_013335665.1:0.40183,GB_GCA_002869265.1:0.34
46)28.0:0.04564)3.0:0.01644,GB_GCA_016784175.1:0.46809)34.0:0.02633)48.0:0.0297,GB_GCA_00
2778705.1:0.59274)62.0:0.02271)100.0:0.05398,GB_GCA_002773655.1:0.43845)'100.0:o__BM507':0
.11086)42.0:0.04646)16.0:0.02672,((GB_GCA_016214745.1:0.43905,(GB_GCA_018824325.1:0.43062
,GB_GCA_018895765.1:0.48526)33.0:0.06757)38.0:0.06018,(GB_GCA_001817505.1:0.51492,((GB_G
CA_016930315.1:0.40866,GB_GCA_007376135.1:0.61008)73.0:0.03628,(GB_GCA_018818885.1:0.33
964,GB_GCA_002773845.1:0.46371)99.0:0.06553)'100.0:o__Buchananbacterales':0.09173)68.0:0.04
532)1.0:0.02192)6.0:0.02394,(((GB_GCA_016209005.1:0.43051,((GB_GCA_002402995.1:0.47241,(GB
_GCA_018896315.1:0.0801352,bin.253:0.0647335):0.420205)100.0:0.11989,(GB_GCA_007378955.1:
0.34036,GB_GCA_002339755.1:0.38108)70.0:0.04883)56.0:0.0474)'64.0:o__UBA2591':0.03704,(GB_
GCA_018896645.1:0.222744,bin.303:0.254114):0.201426)61.0:0.03931,(GB_GCA_011682665.1:0.47
275,GB_GCA_018053685.1:0.6901)18.0:0.06893)15.0:0.03758)10.0:0.02826)6.0:0.03261,((GB_GCA_
005239895.1:0.74078,(GB_GCA_002774425.1:0.53076,GB_GCA_007375725.1:0.62913)25.0:0.06593)
15.0:0.05552,((GB_GCA_016206145.1:0.67239,((GB_GCA_001791465.1:0.60433,GB_GCA_01621532
5.1:0.62892)43.0:0.07911,GB_GCA_016217875.1:0.62537)10.0:0.05034)1.0:0.03225,(GB_GCA_0017
91425.1:0.62413,((GB_GCA_001790815.1:0.49873,(GB_GCA_016206165.1:0.42018,GB_GCA_016188
355.1:0.58417)49.0:0.07358)39.0:0.0688,(((GB_GCA_001791745.1:0.59975,(GB_GCA_903870305.1:0
.5023,GB_GCA_004297205.1:0.48468)64.0:0.05618)73.0:0.04867,(GB_GCA_016203535.1:0.72128,((
GB_GCA_003497135.1:0.49,(GB_GCA_016187485.1:0.51584,(((GB_GCA_002791295.1:0.246735,bin.
251:0.204187):0.00724188,bin.153:0.196559):0.183293,bin.405:0.300894):0.0716897)44.0:0.0286)5
6.0:0.02698,((GB_GCA_002782865.1:0.27504,bin.430:0.238099):0.27724,((GB_GCA_013203725.1:9.
48074e-06,bin.195:6.11352e-
06):0.567557,bin.471:0.336782):0.0537635)68.0:0.061)100.0:0.09729)77.0:0.0414)'71.0:o__SG8-
24':0.05394,(GB_GCA_002688135.1:0.6197,((GB_GCA_003501255.1:0.51739,((GB_GCA_016182085.
1:0.358936,bin.476:0.279963):0.163084,GB_GCA_018661775.1:0.73151)100.0:0.09488)100.0:0.0759
,((GB_GCA_001001925.1:0.4293,GB_GCA_001783145.1:0.43126)100.0:0.10707,GB_GCA_001782975
.1:0.66963)45.0:0.03483)83.0:0.04288)'98.0:o__Magasanikbacterales':0.06441)61.0:0.04245)28.0:0.
03434)9.0:0.03169)3.0:0.02282)9.0:0.04421)26.0:0.02868,((((GB_GCA_001003705.1:0.43536,GB_GC
A_016214565.1:0.44122)'100.0:o__UBA10025':0.10418,(GB_GCA_018814765.1:0.46937,GB_GCA_00
2784945.1:0.42008)'74.0:o__UM-FILTER-42-
10':0.04959)37.0:0.0501,(GB_GCA_003597955.1:0.62251,(GB_GCA_016192205.1:0.52511,(GB_GCA_
016204585.1:0.5012,GB_GCA_011047195.1:0.58224)65.0:0.05187)100.0:0.08576)'78.0:o__SBBC01':
0.08079)48.0:0.05217,(((GB_GCA_005239655.1:0.61611,(GB_GCA_018822435.1:0.37661,GB_GCA_0
16182495.1:0.51414)100.0:0.09191)'95.0:o__UBA9570':0.0786,(GB_GCA_016182525.1:0.49056,GB_
GCA_001818775.1:0.51842)25.0:0.05175)57.0:0.04705,GB_GCA_004298615.1:0.63192)26.0:0.03226
)37.0:0.03885)'95.0:c__ABY1':0.07542)33.0:0.03555,(((GB_GCA_001824325.1:0.84086,(((GB_GCA_01

4238475.1:0.95878,(((GB_GCA_002376885.1:0.43973,(GB_GCA_002774795.1:0.53397,GB_GCA_016
215345.1:0.50491)80.0:0.05323)41.0:0.044,(GB_GCA_001823065.1:0.50431,(GB_GCA_001823095.1:
0.44526,GB_GCA_016181465.1:0.45856)24.0:0.06583)60.0:0.05917)95.0:0.0457,(((((GB_GCA_00182
1395.1:0.5511,GB_GCA_014383885.1:0.44873)21.0:0.04205,GB_GCA_016214545.1:0.43481)13.0:0.0
295,(GB_GCA_903824855.1:0.43632,((GB_GCA_001823605.1:0.41115,(GB_GCA_016201355.1:0.489
79,GB_GCA_007375595.1:0.3915)48.0:0.04637)46.0:0.03906,GB_GCA_018814635.1:0.47599)56.0:0.
02915)45.0:0.04085)77.0:0.0349,GB_GCA_016176785.1:0.52774)56.0:0.03615,(((((GB_GCA_0142379
15.1:0.66065,GB_GCA_014378965.1:0.6538)1.0:0.09955,(((((GB_GCA_017881075.1:0.35303,(GB_GC
A_903854195.1:0.30702,(GB_GCA_903848795.1:0.33188,GB_GCA_018062305.1:0.31253)95.0:0.125
65)28.0:0.08027)17.0:0.04779,((GB_GCA_003455555.1:0.40191,GB_GCA_016215405.1:0.45369)37.0
:0.06587,((((GB_GCA_903954165.1:0.37409,((GB_GCA_001821135.1:0.48589,(GB_GCA_007375925.
1:0.38409,GB_GCA_001820955.1:0.40213)30.0:0.04822)47.0:0.04835,GB_GCA_018968805.1:0.3657)
23.0:0.02889)36.0:0.02991,(GB_GCA_000999175.1:0.42414,GB_GCA_016181525.1:0.49774)25.0:0.0
2483)28.0:0.03861,(GB_GCA_001822655.1:0.44771,GB_GCA_002787175.1:0.3788)25.0:0.05082)41.
0:0.03275,(GB_GCA_903870235.1:0.63358,GB_GCA_903823865.1:0.54903)60.0:0.03385)13.0:0.0168
7)20.0:0.02601)3.0:0.02045,(GB_GCA_903882905.1:0.39389,GB_GCA_903933085.1:0.35985)59.0:0.
08761)7.0:0.01984,((GB_GCA_016860785.1:0.40226,GB_GCA_001824215.1:0.51395)48.0:0.05961,(G
B_GCA_002791475.1:0.437072,bin.649:0.299941):0.0697181)40.0:0.03225)10.0:0.0396,(GB_GCA_00
7125155.1:0.59612,(GB_GCA_002787695.1:0.48095,GB_GCA_018061745.1:0.50944)41.0:0.0398)8.0:
0.03192)5.0:0.02516)0.0:0.0209,((GB_GCA_018401295.1:0.45346,(GB_GCA_002771565.1:0.44878,G
B_GCA_002771585.1:0.47163)52.0:0.04111)44.0:0.03004,(GB_GCA_002401445.1:0.4178,GB_GCA_0
07376725.1:0.41039)28.0:0.0705)5.0:0.02459)1.0:0.02347,((((GB_GCA_002327585.1:0.326248,bin.4
57:0.325285):0.0607421,GB_GCA_001781435.1:0.44308)10.0:0.05227,((GB_GCA_002778575.1:0.33
2546,bin.102:0.311432):0.0908639,(((GB_GCA_000998805.1:0.58784,(GB_GCA_002255585.1:0.6283
1,GB_GCA_002710785.1:0.62036)41.0:0.02991)57.0:0.03104,GB_GCA_903878285.1:0.71542)16.0:0.
03198,GB_GCA_016217935.1:0.71084)28.0:0.0382)16.0:0.04585)52.0:0.03803,(GB_GCA_003022255
.1:0.74041,GB_GCA_016699465.1:0.41835)10.0:0.12245)4.0:0.02712)1.0:0.04543,((GB_GCA_018062
965.1:0.63454,(GB_GCA_007117675.1:0.53262,GB_GCA_001785725.1:0.65887)51.0:0.05518)28.0:0.
04594,GB_GCA_903869825.1:0.72392)41.0:0.06307)4.0:0.02454)3.0:0.04242)26.0:0.09789)'96.0:o__
UBA9983_A':0.1791,(((GB_GCA_012510515.1:0.55616,(GB_GCA_012798235.1:0.64006,GB_GCA_002
898235.1:0.89082)59.0:0.06016)13.0:0.05903,(GB_GCA_016217555.1:0.65508,(GB_GCA_001824435
.1:0.5574,(((GB_GCA_016176505.1:0.57291,((GB_GCA_001818015.1:0.53086,((GB_GCA_001003205.
1:0.42226,GB_GCA_016214655.1:0.4341)61.0:0.04624,((GB_GCA_002773415.1:0.53956,GB_GCA_00
2773535.1:0.43179)55.0:0.04352,GB_GCA_003539915.1:0.47289)59.0:0.03964)96.0:0.04253)54.0:0.
02936,(GB_GCA_016215765.1:0.41068,GB_GCA_001793855.1:0.37242)59.0:0.06316)13.0:0.03055)8
.0:0.01994,((GB_GCA_001779575.1:0.4695,(GB_GCA_001003475.1:0.47764,GB_GCA_002781185.1:0
.39673)100.0:0.07886)80.0:0.0603,(GB_GCA_016867315.1:0.59701,(GB_GCA_001821375.1:0.52012,
GB_GCA_016182095.1:0.43823)67.0:0.06633)69.0:0.04311)6.0:0.0226)43.0:0.02798,GB_GCA_00179
3865.1:0.69601)95.0:0.07826)98.0:0.05376)64.0:0.06205)7.0:0.03127,GB_GCA_001001795.1:0.9035
5)'90.0:o__UBA6257':0.05394)7.0:0.04334,(((GB_GCA_016204035.1:0.62488,(GB_GCA_016186395.1
:0.51015,(GB_GCA_001794055.1:0.55503,(GB_GCA_016181175.1:0.42881,GB_GCA_016185525.1:0.
42125)97.0:0.04551)100.0:0.06818)'100.0:o__2-02-FULL-40-
12':0.1342)50.0:0.06137,(((GB_GCA_016188775.1:0.46426,GB_GCA_003599335.1:0.52704)61.0:0.07
014,(GB_GCA_016196625.1:0.5684,GB_GCA_016186325.1:0.58466)57.0:0.0743)28.0:0.04309,((GB_
GCA_016201505.1:0.52774,((GB_GCA_018971745.1:0.38283,GB_GCA_016186145.1:0.4015)72.0:0.0
3966,GB_GCA_016186095.1:0.47206)91.0:0.04906)'100.0:o__Sungbacterales':0.11018,((GB_GCA_00
2793915.1:0.59697,GB_GCA_001821765.1:0.58409)'87.0:o__Ryanbacterales':0.06048,((GB_GCA_00
1778905.1:0.5802,GB_GCA_001001285.1:0.56175)89.0:0.07671,(((GB_GCA_016181935.1:0.51509,G

B_GCA_002772995.1:0.51613)91.0:0:0.04585,GB_GCA_001821615.1:0.57955)80.0:0:0.05818,((GB_GCA
_016186495.1:0.4359,GB_GCA_016179715.1:0.51968)31.0:0:0.04484,((GB_GCA_018817995.1:0.32184
1,bin.131:0.244076):0.181289,(GB_GCA_002772825.1:0.447,GB_GCA_013152035.1:0.47907)67.0:0:0.
06142)73.0:0:0.09464)9.0:0:0.03352)41.0:0:0.03)'63.0:o__UBA9983':0.05084)66.0:0:0.08727)64.0:0:0.05475)1
4.0:0:0.02806)8.0:0:0.02545,((GB_GCA_001774105.1:0.39182,((GB_GCA_000992345.1:0.41512,(GB_GCA
_003056085.1:0.39308,GB_GCA_016200965.1:0.48069)100.0:0:0.09776)78.0:0:0.04973,GB_GCA_01619
5045.1:0.68118)'86.0:o__GWC2-36-
17':0.05173)35.0:0:0.05457,(((GB_GCA_016177905.1:0.48197,(GB_GCA_016188605.1:0.49603,GB_GC
A_001820055.1:0.53435)73.0:0:0.07464)'100.0:o__Spechtbacterales':0.12366,((GB_GCA_000996085.1
:0.47718,GB_GCA_001824745.1:0.62292)'100.0:o__Terrybacterales':0.16468,GB_GCA_018609185.1:
0.40925)38.0:0:0.05657)37.0:0:0.0506,((GB_GCA_002787255.1:0.40507,(((((GB_GCA_003556925.1:0.52
836,(GB_GCA_003557065.1:0.58157,GB_GCA_002069755.1:0.52129)94.0:0:0.05398)55.0:0:0.04386,((GB
_GCA_018817645.1:0.269522,bin.440:0.217435):0.236878,GB_GCA_017991975.1:0.61019)50.0:0:0.06
675)33.0:0:0.03889,(GB_GCA_011338085.1:0.56554,(GB_GCA_002923395.1:0.54118,GB_GCA_015491
015.1:0.54928)66.0:0:0.11026)33.0:0:0.06483)2.0:0:0.02904,(((GB_GCA_016188735.1:0.69724,GB_GCA_0
18895125.1:0.58121)19.0:0:0.04726,(GB_GCA_002452735.1:0.60238,(GB_GCA_002779355.1:0.38494,
GB_GCA_002423555.1:0.59348)70.0:0:0.05543)50.0:0:0.03839)4.0:0:0.02273,((GB_GCA_001821475.1:0.4
0507,(GB_GCA_011355095.1:0.62701,GB_GCA_903845295.1:0.67316)78.0:0:0.04439)50.0:0:0.03274,((G
B_GCA_007376185.1:0.38797,(GB_GCA_018894495.1:0.31407,GB_GCA_018813025.1:0.45669)10.0:
0.02532)10.0:0:0.03924,((GB_GCA_016186485.1:0.39125,(GB_GCA_002787295.1:0.2604,(GB_GCA_01
6181965.1:0.35397,bin.173:0.180999):0.0695298)39.0:0:0.0287)71.0:0:0.03522,(GB_GCA_011050465.1:
0.35438,((GB_GCA_000398025.1:0.22905,(GB_GCA_002781125.1:0.1935,GB_GCA_001303125.1:0.2
4979)53.0:0:0.0421)89.0:0:0.04261,(GB_GCA_016782605.1:0.241438,bin.324:0.259834):0.0571624)74.0
:0.05524)8.0:0:0.01929)23.0:0:0.03113)42.0:0:0.04164)11.0:0:0.0318)6.0:0:0.0301)15.0:0:0.05745,(GB_GCA_00
2788555.1:0.57364,GB_GCA_002329255.1:0.69055)17.0:0:0.11142)15.0:0:0.03052)'81.0:o__Paceibacter
ales':0.08123,((GB_GCA_018829905.1:0.55416,GB_GCA_000992445.2:0.48425)57.0:0:0.06005,((GB_G
CA_002793835.1:0.55338,GB_GCA_018899995.1:0.53405)38.0:0:0.09927,((((GB_GCA_002791035.1:0.
320304,bin.239:0.259056):0.0172783,bin.79:0.238151):0.173208,(GB_GCA_002792395.1:0.42252,G
B_GCA_016208745.1:0.36387)91.0:0:0.06082)51.0:0:0.02998,GB_GCA_001872305.1:0.62528)48.0:0:0.036
3)'13.0:o__Portnoybacterales':0.02944)15.0:0:0.04026)5.0:0:0.03348)1.0:0:0.02294)3.0:0:0.02594)6.0:0:0.034
73)9.0:0:0.03813)22.0:0:0.03128,GB_GCA_003565105.1:0.8493)54.0:0:0.038,(((GB_GCA_011362555.1:0.4
1012,(GB_GCA_018399935.1:0.45023,GB_GCA_015493225.1:0.46979)97.0:0:0.04671)100.0:0:0.1477,(((
GB_GCA_018061645.1:0.49166,GB_GCA_018401135.1:0.56321)100.0:0:0.05369,(GB_GCA_002400405
.1:0.43583,(((GB_GCA_018400055.1:0.26847,(GB_GCA_000990585.1:0.25499,GB_GCA_003056165.1
:0.33621)94.0:0:0.04316)57.0:0:0.03171,GB_GCA_002923195.1:0.26437)55.0:0:0.02436,(GB_GCA_004297
805.1:0.3507,GB_GCA_013336225.1:0.36819)96.0:0:0.03927)63.0:0:0.02733)69.0:0:0.03848)98.0:0:0.06948,
GB_GCA_002790655.1:0.4043)100.0:0:0.06477)'100.0:o__Moranbacterales':0.11333,(GB_GCA_90389
8335.1:0.68791,GB_GCA_018399475.1:0.57971)'100.0:o__CAIUEV01':0.11623)100.0:0:0.08572)'76.0:c
__Paceibacteria':0.05014)31.0:0:0.03243)63.0:0:0.06034,(GB_GCA_018830605.1:0.69961,(((((GB_GCA_0
02305395.1:0.64233,(GB_GCA_002778735.1:0.52764,GB_GCA_002071685.1:0.5482)100.0:0:0.1747)'9
9.0:o__UBA1384':0.09965,(GB_GCA_007376535.1:0.62922,(GB_GCA_000991185.1:0.58324,GB_GCA
_002780235.1:0.59739)54.0:0:0.09802)'100.0:o__GWA2-35-
9':0.12484)73.0:0:0.05634,((GB_GCA_009885355.1:0.46877,GB_GCA_001774395.1:0.78917)68.0:0:0.08
834,(GB_GCA_016844295.1:0.34937,GB_GCA_001029735.1:0.37475)'100.0:o__UBA12157':0.17656)
28.0:0:0.04749)16.0:0:0.03156,(GB_GCA_011332705.1:0.57442,GB_GCA_002771215.1:0.45835)51.0:0:0.1
151)17.0:0:0.04677,((GB_GCA_903851235.1:0.42386,(GB_GCA_002773985.1:0.4017,GB_GCA_002070
055.1:0.4548)100.0:0:0.08805)'100.0:o__XYA2-FULL-43-
10':0.19661,((GB_GCA_903834195.1:0.69831,GB_GCA_903954185.1:0.62696)49.0:0:0.10443,(GB_GCA

_016198415.1:0.73032,GB_GCA_011334535.1:0.73088)10.0:0.06629)17.0:0.04875)14.0:0.03551)60.0:0.05102)'88.0:c__UBA1384':0.07515)15.0:0.03134)65.0:0.04096)73.0:0.0502,((((GB_GCA_016699595.1:0.81692,((GB_GCA_016788825.1:0.54345,GB_GCA_016861265.1:0.54487)'100.0:o__J137':0.20946,(GB_GCA_018816645.1:0.62304,(GB_GCA_016931155.1:0.43268,(GB_GCA_001567355.1:0.52492,(GB_GCA_016927655.1:0.43528,GB_GCA_014729085.1:0.49814)60.0:0.05478)34.0:0.04475)'97.0:o__B142':0.06514)76.0:0.05648)36.0:0.0514)100.0:0.09426,((GB_GCA_016929645.1:0.57236,(GB_GCA_014729235.1:0.48171,(GB_GCA_003560355.1:0.46305,GB_GCA_016182915.1:0.52945)96.0:0.06365)100.0:0.12635)'100.0:o__SC72':0.10531,(GB_GCA_016927995.1:0.78189,GB_GCA_015489595.1:0.86296)86.0:0.08694)100.0:0.10541)'100.0:c__Dojkabacteria':0.16496,(((GB_GCA_018818265.1:0.63863,GB_GCA_013335095.1:0.72118)'100.0:c__4484-211;o__4484-
211':0.17482,(GB_GCA_011057755.1:0.81625,(GB_GCA_002774225.1:0.7943,((GB_GCA_001772615.1:0.47651,GB_GCA_001773295.1:0.71264)'100.0:o__UBA101185':0.13173,((GB_GCA_009995545.1:0.75441,GB_GCA_002789015.1:0.68506)'100.0:o__2-01-FULL-39-
13':0.11979,(GB_GCA_002770755.1:0.56024,GB_GCA_002774285.1:0.62224)99.0:0.08555)71.0:0.04313)99.0:0.05806)60.0:0.04438)'93.0:c__WWE3':0.05768)30.0:0.03983,((GB_GCA_001771135.1:0.5053,GB_GCA_002791435.1:0.47593)'100.0:c__CPR3;o__CPR3':0.22023,(((GB_GCA_001816835.1:0.50353,GB_GCA_001816645.1:0.48307)'100.0:o__Woykebacterales':0.15068,(GB_GCA_001774865.1:0.78193,GB_GCA_016198275.1:0.67847)83.0:0.06289)73.0:0.06638,(((((GB_GCA_016182365.1:0.57513,((GB_GCA_016204905.1:0.35385,GB_GCA_016195685.1:0.4932)'100.0:o__2-02-FULL-39-
11':0.10715,(GB_GCA_000995415.1:0.44675,GB_GCA_018062625.1:0.62489)'100.0:o__UBA10105':0.08485)71.0:0.03891)43.0:0.03533,(GB_GCA_016199065.1:0.5622,(GB_GCA_016182055.1:0.39136,(GB_GCA_016204525.1:0.63982,GB_GCA_016182425.1:0.55195)100.0:0.06781)'100.0:o__Levybacterales':0.09519)56.0:0.04698)57.0:0.04995,(GB_GCA_016782545.1:0.48732,(GB_GCA_002788855.1:0.56799,GB_GCA_001788215.1:0.50959)'99.0:o__GCA-
001788215':0.0873)82.0:0.07807)66.0:0.04012,(GB_GCA_015486555.1:0.87306,(GB_GCA_001788295.1:0.77337,(GB_GCA_001788095.1:0.57316,GB_GCA_001789855.1:0.77251)100.0:0.09697)'100.0:o__UBA1406':0.14535)68.0:0.08534)39.0:0.05027,(((((GB_GCA_016219325.1:0.511393,bin.361:0.597788):0.162887,(((GB_GCA_003176165.1:0.55009,(GB_GCA_002772705.1:0.78396,GB_GCA_002305195.1:0.59443)75.0:0.05458)100.0:0.14129,(GB_GCA_001803465.1:0.45268,GB_GCA_001803415.1:0.55544)95.0:0.06677)94.0:0.06688,(GB_GCA_016176555.1:0.50119,GB_GCA_001816585.1:0.49021)60.0:0.07822)17.0:0.04107)23.0:0.02799,((RS_GCF_009936135.1:0.47797,GB_GCA_018819325.1:0.53518)100.0:0.16693,GB_GCA_903945195.1:0.74573)56.0:0.07232)98.0:0.05395,((GB_GCA_018813815.1:0.48288,GB_GCA_001029675.1:0.4994)72.0:0.09103,(GB_GCA_002773945.1:0.48845,GB_GCA_012797845.1:0.61768)28.0:0.03819)95.0:0.05624)'98.0:o__UBA1400':0.06019,((((GB_GCA_002774085.1:0.56785,(GB_GCA_018902845.1:0.68163,(GB_GCA_018830585.1:0.49488,(GB_GCA_016783425.1:0.49306,GB_GCA_015485895.1:0.56283)98.0:0.07615)83.0:0.06738)'100.0:o__UBA12405':0.1569)77.0:0.07069,(GB_GCA_002780305.1:0.58313,(GB_GCA_002784465.1:0.45483,GB_GCA_016866735.1:0.47259)39.0:0.06239)81.0:0.07393)54.0:0.03961,(GB_GCA_016927565.1:0.5023,(GB_GCA_018900995.1:0.32189,GB_GCA_002793635.1:0.40139)81.0:0.08061)'100.0:o__PFEM01':0.11396)36.0:0.04361,((GB_GCA_000993205.1:0.78215,(GB_GCA_016204995.1:0.50544,GB_GCA_001816475.1:0.51935)70.0:0.04982)100.0:0.09951,(GB_GCA_000999595.1:0.55481,GB_GCA_007376345.1:0.68699)96.0:0.09565)'94.0:o__GWA2-44-
7':0.06537)43.0:0.04591)66.0:0.0508)79.0:0.06968)'100.0:c__Microgenomatia':0.07267)46.0:0.04374)100.0:0.0806)96.0:0.0569,(GB_GCA_903889905.1:0.40715,GB_GCA_001873755.1:0.36515)'100.0:c__CG2-30-54-11;o__CG2-30-54-
11':0.43444)17.0:0.03796)'99.0:p__Patescibacteria':0.12863,((GB_GCA_016932385.1:0.45572,GB_GCA_002050035.1:0.47035)'100.0:p__4484-113;c__4484-113;o__4484-
113':0.48022,(GB_GCA_011368175.1:0.33476,GB_GCA_002069885.1:0.38442)'100.0:p__UBP15;c__

UBA6099;o__UBA6099':0.33896)75.0:0.09446)23.0:0.05265,(((((GB_GCA_003503675.1:0.30629,(GB_
GCA_016867265.1:0.4975,GB_GCA_017744355.1:0.28068)100.0:0.0615)'100.0:o__S15B-
MN24':0.13285,(GB_GCA_014377495.1:0.43851,(GB_GCA_014377055.1:0.46228,GB_GCA_00278340
5.1:0.52575)'98.0:o__UBA7694':0.06231)99.0:0.0837)'100.0:c__Sericytochromatia':0.0922,(((GB_GC
A_013285555.1:0.33453,GB_GCA_014379585.1:0.34049)'100.0:o__Gloeobacterales':0.0891,((RS_GC
F_000332275.1:0.22551,RS_GCF_000013225.1:0.23621)'100.0:o__PCC-
7336':0.10631,(((GB_GCA_012032135.1:0.29135,((GB_GCA_004292515.1:0.24901,(RS_GCF_0038464
45.1:0.19345,GB_GCA_012032745.1:0.25163)78.0:0.04497)42.0:0.02703,(GB_GCA_903897715.1:0.2
4601,((((GB_GCA_012032525.1:0.23588,GB_GCA_014534385.1:0.27154)71.0:0.01974,(((RS_GCF_00
1939115.1:0.13502,RS_GCF_014695385.1:0.09933)74.0:0.01702,RS_GCF_000775285.1:0.16966)72.0
:0.01518,RS_GCF_001650195.1:0.21892)75.0:0.01531)36.0:0.01028,RS_GCF_000317045.1:0.1577)43
.0:0.01016,((((((GB_GCA_010672865.1:0.1451,(((RS_GCF_000332195.1:0.26065,(((GB_GCA_0181417
85.1:0.18808,GB_GCA_011391125.1:0.16932)28.0:0.01951,(GB_GCA_011088145.1:0.3392,(GB_GCA
_016903655.1:0.15867,RS_GCF_000332035.1:0.20343)56.0:0.0228)10.0:0.01536)4.0:0.01347,(RS_G
CF_000017845.1:0.21104,(GB_GCA_903887375.1:0.26463,RS_GCF_000297435.1:0.23973)37.0:0.011
45)69.0:0.02601)11.0:0.01274)41.0:0.01096,GB_GCA_012031815.1:0.20209)81.0:0.01894,GB_GCA_
012031265.1:0.27004)46.0:0.0119)81.0:0.01585,GB_GCA_018610095.1:0.28394)100.0:0.04268,RS_G
CF_000211815.1:0.18616)28.0:0.01382,GB_GCA_012031635.1:0.24244)34.0:0.02105,(((RS_GCF_014
698505.1:0.1774,GB_GCA_905479905.1:0.22165)100.0:0.0296,RS_GCF_015207735.1:0.17328)98.0:0
.01465,(RS_GCF_003003775.1:0.23644,RS_GCF_014695915.1:0.1257)42.0:0.01761)23.0:0.01692)43.
0:0.01792,(GB_GCA_005518205.2:0.19957,((GB_GCA_015295835.1:0.15603,(RS_GCF_012295525.1:
0.17341,(GB_GCA_001314905.1:0.26757,RS_GCF_001870905.1:0.23438)65.0:0.02478)44.0:0.01446)
16.0:0.01013,RS_GCF_013179805.1:0.21885)32.0:0.01155)22.0:0.00982)'76.0:o__Cyanobacteriales':
0.02566)32.0:0.01394)50.0:0.0167)37.0:0.01929)56.0:0.03625,(RS_GCF_014696065.1:0.1891,GB_GC
A_002291895.1:0.4007)19.0:0.02722)31.0:0.03861,(RS_GCF_001870225.1:0.3527,(((RS_GCF_014279
975.1:0.158566,bin.275:0.120401):0.0303117,bin.183:0.108655):0.00178569,bin.51:0.114466):0.424
297)60.0:0.03856)94.0:0.05617)95.0:0.06973)'100.0:c__Cyanobacteriia':0.29149,((GB_GCA_9021682
45.1:0.51339,(GB_GCA_001784535.1:0.30596,(RS_GCF_904420445.1:0.23063,(GB_GCA_015152565.
1:0.46738,GB_GCA_002438405.1:0.40358)100.0:0.10361)100.0:0.05606)'100.0:o__Gastranaerophila
les':0.1548)100.0:0.09618,(GB_GCA_003153555.1:0.5719,(GB_GCA_001784585.1:0.56563,(GB_GCA_
001899385.1:0.74897,GB_GCA_018695135.1:0.62501)'65.0:o__Caenarcaniphilales':0.05751)100.0:0.
08824)100.0:0.0661)'100.0:c__Vampirovibrionia':0.07912)95.0:0.0436)'100.0:p__Cyanobacteria':0.1
0178,((GB_GCA_018823125.1:0.38128,((GB_GCA_011333685.1:0.25229,(GB_GCA_013204645.1:9.83
673e-06,bin.592:6.11352e-06):0.32232)'100.0:o__O2-12-FULL-45-
9':0.08652,((GB_GCA_001771535.1:0.36435,GB_GCA_009772965.1:0.34797)100.0:0.05823,(GB_GCA
_018813405.1:0.3189,(GB_GCA_013202975.1:5.35217e-06,bin.294:6.11352e-
06):0.350755)75.0:0.04219)'100.0:o__XYC2-FULL-46-14':0.06196)99.0:0.0651)'100.0:c__WOR-
1':0.16665,(GB_GCA_003864475.1:0.65809,((GB_GCA_018646105.1:0.50153,((GB_GCA_009691415.
1:0.38195,(GB_GCA_018402095.1:0.4023,GB_GCA_005791325.1:0.60087)100.0:0.08876)'100.0:o__S
XXA01':0.09624,((GB_GCA_013215865.1:0.42112,GB_GCA_018698675.1:0.47433)33.0:0.06634,(((GB
_GCA_018700055.1:0.41216,GB_GCA_902558105.1:0.50708)'84.0:o__UBA817':0.07212,GB_GCA_00
2433595.1:0.72684)40.0:0.04651,((GB_GCA_002716485.1:0.42714,GB_GCA_003265895.1:0.40312)7
3.0:0.08747,(GB_GCA_002719695.1:0.55198,GB_GCA_003265975.1:0.41178)33.0:0.04039)'99.0:o__
GCA-
2716485':0.09314)15.0:0.02967)11.0:0.03044)28.0:0.0509)'100.0:c__Marinamargulisbacteria':0.2162
2,(GB_GCA_003242895.1:0.43001,GB_GCA_903897765.1:0.71497)'100.0:c__Riflemargulisbacteria':0.
09565)44.0:0.04262)100.0:0.07237)'100.0:p__Margulisbacteria':0.08933)98.0:0.04351,(((((((((GB_G
CA_001028815.1:0.28795,((GB_GCA_007132635.1:0.29117,GB_GCA_900019385.1:0.35468)90.0:0.0

4508,(GB_GCA_012728115.1:0.21943,GB_GCA_003551965.1:0.28896)56.0:0.02809)68.0:0.03059)'10
0.0:o__DTU010':0.14935,(GB_GCA_012727955.1:0.29011,GB_GCA_012735435.1:0.36587)'100.0:o__
DTU087':0.06662)64.0:0.03525,(GB_GCA_012522085.1:0.37241,((GB_GCA_012840225.1:0.37471,(G
B_GCA_012522135.1:0.29962,GB_GCA_002919235.1:0.27361)85.0:0.03761)'100.0:o__DTU080':0.04
95,((GB_GCA_017577405.1:0.35591,(GB_GCA_014896295.1:0.40344,GB_GCA_014896305.1:0.41119
)60.0:0.04943)'69.0:o__Limnochordales':0.03995,GB_GCA_017578085.1:0.31431)98.0:0.0469)96.0:0.
03225)16.0:0.02205)13.0:0.02342,GB_GCA_012839765.1:0.39592)'97.0:c__Limnochordia':0.03967,((
GB_GCA_002427165.1:0.43817,GB_GCA_012842235.1:0.53436)56.0:0.04294,(GB_GCA_014896355.
1:0.37135,GB_GCA_002408345.1:0.36667)'95.0:c__UBA5301':0.0643)46.0:0.0297)97.0:0.03453,(((G
B_GCA_012689375.1:0.51196,(GB_GCA_012839815.1:0.22045,GB_GCA_012842565.1:0.27295)'98.0:
o__Ch115':0.04822)100.0:0.06873,(GB_GCA_900016865.1:0.43316,(GB_GCA_002070415.1:0.47142,
(GB_GCA_900761905.1:0.3273,GB_GCA_019136685.1:0.29842)100.0:0.07181)'100.0:o__DTUO25':0.
11263)77.0:0.04124)'97.0:c__SHA-
98':0.05636,(GB_GCA_002426645.1:0.42302,(GB_GCA_002399265.1:0.38703,(GB_GCA_012516115.
1:0.35306,(GB_GCA_012728785.1:0.4084,(GB_GCA_002385625.1:0.2509,GB_GCA_012512605.1:0.4
2386)'96.0:o__UBA10575':0.05994)94.0:0.04653)99.0:0.05944)'100.0:c__UBA4882':0.08989)67.0:0.0
337)83.0:0.02992)'100.0:p__Firmicutes_G':0.04173,((((GB_GCA_012522585.1:0.35054,GB_GCA_009
783675.1:0.41503)'100.0:o__JAAYKD01':0.2077,(RS_GCF_014931565.1:0.52389,(GB_GCA_01273550
5.1:0.30374,GB_GCA_018333655.1:0.49847)100.0:0.06839)95.0:0.05362)'94.0:p__Firmicutes_H;c__
UBA994':0.06001,(GB_GCA_014896485.1:0.52033,((((RS_GCF_001642725.1:0.40623,RS_GCF_01464
7315.1:0.40263)'100.0:o__Alicyclobacillales':0.05185,(RS_GCF_000376225.1:0.17508,RS_GCF_00224
3515.1:0.2163)'100.0:o__Tumebacillales':0.08792)75.0:0.03034,RS_GCF_000092905.1:0.29294)95.0:
0.036,(RS_GCF_001730225.1:0.36446,(((GB_GCA_002159075.1:0.24459,RS_GCF_900538055.1:0.647
51)3.0:0.0361,(RS_GCF_014646935.1:0.1863,(RS_GCF_000510645.1:0.17837,RS_GCF_003633725.1:0
.53933)89.0:0.10532)51.0:0.03032)9.0:0.03035,((RS_GCF_001375555.1:0.25972,((RS_GCF_90010268
5.1:0.27038,(RS_GCF_018326425.1:0.09602,RS_GCF_003001905.1:0.13863)79.0:0.0265)81.0:0.0232
9,RS_GCF_004343255.1:0.2494)83.0:0.07714)'81.0:o__Thermoactinomycetales':0.05679,((((RS_GCF_
014644735.1:0.1692,RS_GCF_016745835.1:0.18245)'100.0:o__Caldalkalibacillales':0.06852,(RS_GCF
_001049895.1:0.28895,((RS_GCF_904373265.1:0.26961,((RS_GCF_001590685.1:0.33726,((RS_GCF_0
12955605.1:0.23283,(((((((RS_GCF_016908515.1:0.25489,(RS_GCF_900110685.1:0.28868,RS_GCF_90
0166625.1:0.26263)52.0:0.01559)100.0:0.08118,RS_GCF_011058155.1:0.26673)61.0:0.02605,(RS_G
CF_001742425.1:0.14812,GB_GCA_016841765.1:0.13521)24.0:0.02545)'5.0:o__Bacillales_D':0.0126
5,((((RS_GCF_007293315.1:0.23678,(RS_GCF_002019605.1:0.14618,(RS_GCF_001866055.1:0.14665,
RS_GCF_019039105.1:0.09121)13.0:0.01164)41.0:0.01738)90.0:0.0229,(GB_GCA_007692495.1:0.36
152,RS_GCF_009765375.1:0.23889)94.0:0.02525)'100.0:o__Bacillales_H':0.06135,((RS_GCF_0076735
25.1:0.22171,GB_GCA_900543345.1:0.51506)32.0:0.01717,(RS_GCF_017303315.1:0.18337,RS_GCF_
003097615.1:0.18903)77.0:0.01508)'72.0:o__Bacillales_G':0.01963)57.0:0.01881,(RS_GCF_00226293
5.1:0.15245,RS_GCF_001375675.1:0.19055)'85.0:o__Bacillales_E':0.03865)47.0:0.01822)2.0:0.01643,
GB_GCA_016841285.1:0.16195)40.0:0.03394,(((RS_GCF_011008845.1:0.13726,RS_GCF_003852715.
1:0.18683)72.0:0.02012,(((RS_GCF_018588585.1:0.12681,RS_GCF_003400205.1:0.12811)61.0:0.015
67,(GB_GCA_002243605.1:0.23817,RS_GCF_007827555.1:0.12447)52.0:0.02423)6.0:0.00897,(RS_GC
F_002019635.1:0.20777,RS_GCF_001274935.1:0.13776)6.0:0.00862)9.0:0.01057)3.0:0.00635,(RS_GC
F_012524115.1:0.16329,RS_GCF_003944835.1:0.14674)28.0:0.01701)'59.0:o__Bacillales':0.01297)19
.0:0.02732,((RS_GCF_019039215.1:0.14261,RS_GCF_001439925.1:0.2087)79.0:0.01306,(((RS_GCF_0
03668575.1:0.10319,(RS_GCF_003315075.1:0.13316,(RS_GCF_004405125.1:0.233,(GB_GCA_015482
585.1:0.1431,RS_GCF_000171615.1:0.10573)28.0:0.0199)40.0:0.01595)46.0:0.01458)40.0:0.0116,RS
_GCF_003600835.1:0.17644)37.0:0.00968,RS_GCF_011008565.1:0.15123)87.0:0.01911)'53.0:o__Bac
illales_B':0.01443)7.0:0.01067)13.0:0.01821,RS_GCF_000383875.1:0.22598)48.0:0.03092)23.0:0.025

3,((((GB_GCA_007692455.1:0.37055,RS_GCF_002884575.1:0.53868)65.0:0.01854,(RS_GCF_0001936
35.1:0.63553,((RS_GCF_001998885.1:0.2505,RS_GCF_000633635.1:0.19206)97.0:0.02438,(RS_GCF_
000313915.1:0.26105,RS_GCF_902460355.1:0.47081)83.0:0.02736)100.0:0.04488)100.0:0.03573)10
0.0:0.1389,RS_GCF_014229245.1:0.26106)'100.0:o__Lactobacillales':0.04764,((RS_GCF_000701685.1
:0.41554,(RS_GCF_002901825.1:0.21759,(RS_GCF_003815035.1:0.23545,RS_GCF_000986795.1:0.28
35)92.0:0.03215)100.0:0.07551)'100.0:o__Staphylococcales':0.06314,((((((((GB_GCA_009784525.1:0.
42786,GB_GCA_002358935.1:0.36917)95.0:0.02537,(RS_GCF_015709225.1:0.64841,GB_GCA_01252
2345.1:0.46059)100.0:0.10102)'100.0:o__Acholeplasmatales':0.08736,((GB_GCA_016935515.1:0.380
94,GB_GCA_017541975.1:0.60347)99.0:0.07582,GB_GCA_003557505.1:0.52489)'99.0:o__Izemoplas
matales':0.07314)79.0:0.02901,(GB_GCA_018394315.1:0.39783,(GB_GCA_012838945.1:0.36533,GB
_GCA_001604495.1:0.52855)'100.0:o__ML615J-
28':0.11219)27.0:0.02586)99.0:0.05425,((((GB_GCA_012839015.1:0.5141,(((GB_GCA_905204225.1:0
.39369,GB_GCA_002437105.1:0.97705)64.0:0.04388,GB_GCA_900540365.1:0.67637)97.0:0.11916,G
B_GCA_017457045.1:0.40408)56.0:0.03645)62.0:0.0323,GB_GCA_000433015.1:0.50714)'98.0:o__RF
N20':0.08153,(GB_GCA_002359025.1:0.53891,GB_GCA_017614055.1:0.77364)'100.0:o__Erysipelotri
chales':0.05467)98.0:0.06786,(GB_GCA_015059245.1:0.82341,(GB_GCA_900551955.1:0.76075,((GB_
GCA_014238225.1:0.91045,((GB_GCA_014238055.1:0.57591,(GB_GCA_001641225.1:0.85324,RS_GC
F_000702705.1:1.06047)60.0:0.04784)75.0:0.04315,(GB_GCA_002135175.2:1.36621,GB_GCA_90031
6595.1:1.02395)72.0:0.07459)36.0:0.03816)68.0:0.03839,(RS_GCF_002930155.1:0.79694,RS_GCF_01
8831625.1:0.50585)98.0:0.10355)98.0:0.11394)'83.0:o__Mycoplasmatales':0.06985)23.0:0.04255)59
.0:0.08182)53.0:0.0507,GB_GCA_016841945.1:0.41552)31.0:0.04543,GB_GCA_009787345.1:0.34232
)53.0:0.04144,(GB_GCA_000798955.1:0.25794,(RS_GCF_005771635.1:0.2184,GB_GCA_003864555.1
:0.4505)98.0:0.08685)'100.0:o__Culicoidibacterales':0.11751)69.0:0.10204)37.0:0.04167)18.0:0.0287
7)1.0:0.02262)22.0:0.03544,RS_GCF_004336985.1:0.34358)67.0:0.08278)13.0:0.02869)20.0:0.03008,
(GB_GCA_017656255.1:0.16667,(RS_GCF_016811915.1:0.32028,(RS_GCF_001274715.1:0.21111,RS_
GCF_004522215.1:0.20257)'92.0:o__Aneurinibacillales':0.02751)69.0:0.02044)100.0:0.02842)22.0:0.
03113,(RS_GCF_001730235.1:0.28057,(GB_GCA_018969805.1:0.4537,(RS_GCF_005938195.1:0.1838
7,((RS_GCF_009910845.1:0.21788,(RS_GCF_015472005.1:0.16482,RS_GCF_018403685.1:0.14795)31
.0:0.02257)41.0:0.02067,(((GB_GCA_002383285.1:0.23264,RS_GCF_011440305.1:0.24994)69.0:0.01
986,(RS_GCF_011421635.1:0.16672,RS_GCF_001644605.1:0.1425)35.0:0.01642)23.0:0.01185,RS_GC
F_014212175.1:0.29931)86.0:0.01989)68.0:0.02711)93.0:0.03161)'100.0:o__Paenibacillales':0.074)1
4.0:0.02205)17.0:0.02945)10.0:0.02099)9.0:0.0405)17.0:0.03204)'35.0:c__Bacilli':0.06183)'45.0:p__F
irmicutes':0.06144)11.0:0.03069,(((((((RS_GCF_000020485.1:0.17875,GB_GCA_016278415.1:0.24911
)99.0:0.03722,((RS_GCF_000166415.1:0.32154,GB_GCA_007130465.1:0.36144)14.0:0.02637,(GB_GC
A_016278435.1:0.19671,(GB_GCA_003557445.1:0.25798,GB_GCA_018334565.1:0.31205)34.0:0.038
29)20.0:0.02665)75.0:0.04443)'100.0:o__Halanaerobiales':0.12278,(RS_GCF_900167185.1:0.2433,RS
_GCF_000328625.1:0.30392)'100.0:o__Halobacteroidales':0.09827)91.0:0.04077,(RS_GCF_00399113
5.1:0.23486,GB_GCA_003551665.1:0.50288)86.0:0.04182)'100.0:p__Firmicutes_F;c__Halanaerobiia':
0.12286,(((GB_GCA_016288255.1:0.47052,((GB_GCA_016842005.1:0.17367,GB_GCA_012728565.1:0
.25731)'100.0:c__Ch29;o__Ch29':0.16857,(((GB_GCA_017357865.1:0.33354,(RS_GCF_000427425.1:0
.28319,RS_GCF_008274215.1:0.33877)79.0:0.04135)'100.0:o__Thermoanaerobacterales':0.04436,RS
_GCF_000166355.1:0.42785)'98.0:c__Thermoanaerobacteria':0.04982,(((GB_GCA_001797185.1:0.37
967,(GB_GCA_002426575.1:0.28117,(RS_GCF_900141845.1:0.44208,RS_GCF_000430995.1:0.31164)
82.0:0.03298)'93.0:o__Clostridiales':0.07657)64.0:0.0362,((GB_GCA_012838085.1:0.27491,((RS_GCF
_001263355.1:0.4541,RS_GCF_017352215.1:0.39627)100.0:0.04199,(GB_GCA_002423395.1:0.42909
,GB_GCA_015058295.1:0.49687)100.0:0.08519)100.0:0.06834)'100.0:o__Eubacteriales':0.07307,(((((
GB_GCA_001282665.1:0.16933,GB_GCA_014360205.1:0.49408)95.0:0.03883,(RS_GCF_004103715.1
:0.20503,RS_GCF_900130025.1:0.15988)100.0:0.0362)99.0:0.06072,RS_GCF_000423145.1:0.69751)8

7.0:0.06421,((GB_GCA_002381185.1:0.36999,GB_GCA_007116605.1:0.39395)100.0:0.13263,((((RS_
GCF_016765695.1:0.16366,(RS_GCF_900107485.1:0.15534,RS_GCF_001855385.1:0.30313)100.0:0.0
5012)77.0:0.02633,(RS_GCF_000387765.1:0.13841,RS_GCF_900129995.1:0.17624)91.0:0.02512)67.0
:0.02001,(RS_GCF_000620125.1:0.1803,(RS_GCF_016820655.1:0.19429,GB_GCA_009711765.1:0.156
36)70.0:0.02899)56.0:0.03256)41.0:0.02901,(GB_GCA_013619125.1:0.43485,RS_GCF_001559865.1:
0.19751)16.0:0.05695)16.0:0.02538)16.0:0.0215)'99.0:o__Tissierellales':0.06735,(((RS_GCF_0169084
35.1:0.25892,(RS_GCF_900113635.1:0.36948,RS_GCF_009910325.1:0.3429)79.0:0.03853)100.0:0.07
097,(RS_GCF_018917235.1:0.36415,RS_GCF_001693775.2:0.55475)100.0:0.07941)99.0:0.02543,(((G
B_GCA_002315385.1:0.42931,GB_GCA_012519645.1:0.21794)100.0:0.12024,RS_GCF_014205875.1:
0.23595)76.0:0.02856,(GB_GCA_016938815.1:0.37997,(RS_GCF_900142245.1:0.19653,GB_GCA_002
869095.1:0.34359)34.0:0.02877)95.0:0.03581)99.0:0.04038)'99.0:o__Peptostreptococcales':0.03753)
100.0:0.04201)96.0:0.03348)26.0:0.01892,((((GB_GCA_012842605.1:0.26823,(GB_GCA_012518395.1
:0.33634,(GB_GCA_000526435.1:0.25434,GB_GCA_002386065.1:0.28329)78.0:0.02777)13.0:0.02275
)'100.0:o__Caldicoprobacterales':0.07691,(GB_GCA_012838995.1:0.25062,RS_GCF_000213255.1:0.3
06)'83.0:o__Mahellales':0.06374)100.0:0.03926,(GB_GCA_905210525.1:0.58037,((((GB_GCA_014384
795.1:0.32905,(RS_GCF_005845105.1:0.29184,RS_GCF_900604345.1:0.33529)94.0:0.05743)98.0:0.0
4296,((GB_GCA_017412025.1:0.61026,GB_GCA_002492415.1:0.42897)100.0:0.06731,GB_GCA_9005
42005.1:0.44151)93.0:0.041)28.0:0.02929,(((GB_GCA_018648645.1:0.37951,GB_GCA_017531505.1:
0.57513)100.0:0.06364,(GB_GCA_910585035.1:0.40082,GB_GCA_002435915.1:0.36269)65.0:0.0553
3)49.0:0.03359,((GB_GCA_002293365.1:0.36072,GB_GCA_017388175.1:0.52433)56.0:0.05596,(GB_
GCA_002329665.1:0.37491,GB_GCA_015056185.1:0.41033)14.0:0.03933)19.0:0.02625)99.0:0.04328
)68.0:0.0305,((GB_GCA_018385275.1:0.4281,GB_GCA_900550805.1:0.48173)87.0:0.04793,(((GB_GC
A_900557585.1:0.71612,(GB_GCA_012512255.1:0.41033,GB_GCA_015055705.1:0.50735)97.0:0.058
)28.0:0.02846,((GB_GCA_017457385.1:0.51726,(GB_GCA_017648525.1:0.6279,GB_GCA_905215695.
1:0.40959)100.0:0.04353)90.0:0.03001,(((GB_GCA_910583995.1:0.48841,GB_GCA_900769205.1:0.5
8739)68.0:0.02736,(GB_GCA_902797685.1:0.55327,GB_GCA_017533005.1:0.44762)83.0:0.03628)71
.0:0.03107,GB_GCA_017631635.1:0.47129)100.0:0.05952)72.0:0.0245)34.0:0.0378,GB_GCA_009786
825.1:0.55031)100.0:0.14129)63.0:0.04436)'99.0:o__Christensenellales':0.06666)99.0:0.04265)10.0:
0.04642,((((GB_GCA_014378955.1:0.29088,RS_GCF_002252565.1:0.16796)'89.0:o__SK-
Y3':0.07154,(GB_GCA_017620795.1:0.37897,((((((((GB_GCA_002315595.1:0.34633,GB_GCA_017435
965.1:0.41732)97.0:0.04796,(GB_GCA_004555655.1:0.59872,(GB_GCA_012521525.1:0.31601,GB_G
CA_002293625.1:0.47925)92.0:0.03817)93.0:0.02624)100.0:0.0333,GB_GCA_018366035.1:0.51383)
74.0:0.02964,GB_GCA_016303065.1:0.54906)100.0:0.07066,(((GB_GCA_017616855.1:0.46028,((GB_
GCA_902798755.1:0.79334,GB_GCA_002371625.1:0.67642)73.0:0.02244,(RS_GCF_904420005.1:0.3
33,GB_GCA_002306975.1:0.31438)56.0:0.03595)75.0:0.03929)79.0:0.02824,(GB_GCA_017405765.1:
0.37419,GB_GCA_014384765.1:0.36243)7.0:0.02726)9.0:0.02226,(((GB_GCA_017507855.1:0.44357,(
GB_GCA_009777355.1:0.48322,(GB_GCA_017453605.1:0.54788,GB_GCA_017618795.1:0.69684)95.
0:0.03738)100.0:0.05381)91.0:0.04235,GB_GCA_015067075.1:0.37439)47.0:0.03134,(((GB_GCA_015
067255.1:0.3318,GB_GCA_900760695.1:0.48149)28.0:0.03131,GB_GCA_017554835.1:0.3736)26.0:0.
0252,(GB_GCA_905207625.1:0.52572,(GB_GCA_900555065.1:0.37436,GB_GCA_016281405.1:0.402
44)70.0:0.04831)93.0:0.0535)27.0:0.02791)19.0:0.02172)23.0:0.0236)'100.0:o__Oscillospirales':0.08
551,(GB_GCA_017625695.1:0.3634,GB_GCA_015068525.1:0.39482)'100.0:o__UMGS1840':0.09132)3
3.0:0.02795,((GB_GCA_009780345.1:0.29021,GB_GCA_017546145.1:0.32086)100.0:0.09485,(GB_GC
A_002314595.1:0.31698,(GB_GCA_900551345.1:0.25077,GB_GCA_009784335.1:0.51438)44.0:0.051
97)61.0:0.04592)'56.0:o__Monoglobales':0.03834)3.0:0.01638,((((GB_GCA_015068925.1:0.3489,GB_
GCA_017622395.1:0.44359)41.0:0.03342,(GB_GCA_017549205.1:0.43496,(GB_GCA_018713805.1:0.
32977,(GB_GCA_905233855.1:0.23856,GB_GCA_015068545.1:0.42901)100.0:0.05301)100.0:0.04248
)'77.0:o__UBA1381':0.02914)9.0:0.01697,(GB_GCA_017503945.1:0.46668,((GB_GCA_012521455.1:0

.27883,(GB_GCA_017451505.1:0.39153,GB_GCA_900766735.1:0.35979)86.0:0.03161)87.0:0.02851,
GB_GCA_900552775.1:0.35856)'100.0:o__UMGS1883':0.05624)33.0:0.02266)11.0:0.01697,GB_GCA_
902784805.1:0.45784)25.0:0.01879)24.0:0.02495)64.0:0.03481)75.0:0.04702,((((GB_GCA_01505654
5.1:0.3347,(GB_GCA_904384205.1:0.50539,GB_GCA_017539905.1:0.3975)18.0:0.02942)28.0:0.0312
9,GB_GCA_017536445.1:0.54345)100.0:0.06865,GB_GCA_017450465.1:0.34674)'100.0:o__TANB77':
0.12099,((((((GB_GCA_016292215.1:0.84245,GB_GCA_007135995.1:0.43816)100.0:0.04756,(GB_GC
A_012511175.1:0.73651,RS_GCF_000025225.2:0.47235)68.0:0.03203)'100.0:o__Saccharofermentan
ales':0.16751,GB_GCA_002070475.1:0.5493)17.0:0.03312,(GB_GCA_017559445.1:0.5091,(GB_GCA_
002308775.1:0.34295,(GB_GCA_017461545.1:0.27325,(GB_GCA_015069135.1:0.24568,GB_GCA_01
7522515.1:0.31152)26.0:0.02382)25.0:0.03305)100.0:0.14424)'74.0:o__UBA1212':0.05416)16.0:0.02
825,((GB_GCA_012744235.1:0.37633,(GB_GCA_009783805.1:0.51242,GB_GCA_009777515.1:0.3588
)59.0:0.07061)10.0:0.04492,GB_GCA_003535395.1:0.41645)11.0:0.02605)36.0:0.03543,((GB_GCA_0
03251775.1:0.27568,GB_GCA_012728015.1:0.35592)100.0:0.05308,(GB_GCA_017961515.1:0.37835,
(((RS_GCF_001262605.1:0.18622,RS_GCF_000015865.1:0.23826)84.0:0.02853,(((GB_GCA_01284039
5.1:0.16643,GB_GCA_013314855.1:0.22043)65.0:0.03475,GB_GCA_012840085.1:0.16345)53.0:0.022
07,(GB_GCA_012519745.1:0.26699,RS_GCF_002051585.1:0.31983)95.0:0.02508)22.0:0.01628)83.0:
0.03835,(GB_GCA_012839865.1:0.24117,GB_GCA_002402315.1:0.30702)35.0:0.03452)37.0:0.02317
)18.0:0.02082)'36.0:o__Acetivibrionales':0.04493)51.0:0.04033)72.0:0.03831)95.0:0.03261,((((GB_GC
A_910587735.1:0.39929,(GB_GCA_002372875.1:0.42625,(((GB_GCA_009787945.1:0.55504,GB_GCA
_009784685.1:0.30433)53.0:0.02989,(GB_GCA_009780015.1:0.35663,GB_GCA_009779755.1:0.5403
2)44.0:0.02681)98.0:0.03804,GB_GCA_009787355.1:0.48294)100.0:0.03616)98.0:0.03266)100.0:0.06
701,((GB_GCA_012799415.1:0.24106,RS_GCF_001466305.1:0.19504)54.0:0.03898,GB_GCA_001994
995.1:0.46814)98.0:0.0427)22.0:0.02068,((GB_GCA_017520425.1:0.60966,GB_GCA_017442735.1:0.
60329)87.0:0.03949,((GB_GCA_002381865.1:0.30728,RS_GCF_004339095.1:0.26131)99.0:0.03747,R
S_GCF_002964605.1:0.26234)74.0:0.03182)50.0:0.04368)94.0:0.04323,GB_GCA_001797255.1:0.441
9)'100.0:o__Lachnospirales':0.10206)94.0:0.0425)19.0:0.02682)'24.0:c__Clostridia':0.03103)42.0:0.0
2114)62.0:0.0277)76.0:0.02533,((RS_GCF_008124715.1:0.18183,GB_GCA_012840135.1:0.18995)'10
0.0:o__Thermosediminibacterales':0.15098,(GB_GCA_002376045.1:0.25646,RS_GCF_017301615.1:0.
22409)'99.0:o__Koleobacterales':0.05168)'100.0:c__Thermosediminibacteria':0.05718)'83.0:p__Firm
icutes_A':0.05409,GB_GCA_007132905.1:0.44215)11.0:0.0259)3.0:0.02351,((RS_GCF_900111575.1:0
.3455,(GB_GCA_003559415.1:0.24153,(GB_GCA_009711825.1:0.22245,GB_GCA_002397155.1:0.346
23)70.0:0.03844)'100.0:o__UBA4975':0.21452)'100.0:c__Proteinivoracia':0.09081,(RS_GCF_0000200
05.1:0.44023,(GB_GCA_007121375.1:0.37732,(((GB_GCA_012512665.1:0.30886,GB_GCA_01283921
5.1:0.46872)100.0:0.0539,(GB_GCA_012512705.1:0.27848,(GB_GCA_003554395.1:0.51965,GB_GCA
_003550565.1:0.40649)100.0:0.06628)60.0:0.02996)'100.0:o__DTU022':0.06308,(GB_GCA_0071361
65.1:0.35635,GB_GCA_018333455.1:0.38674)70.0:0.03606)56.0:0.0266)'100.0:c__Dethiobacteria':0.
05179)99.0:0.0385)'67.0:p__Firmicutes_D':0.03853)2.0:0.02493,((GB_GCA_012518435.1:0.34155,GB
_GCA_012837285.1:0.42783)'100.0:p__DTU030;c__DTU030':0.1189,(((((RS_GCF_001263415.1:0.170
1,GB_GCA_002339745.1:0.23379)'100.0:c__Thermincolia;o__Thermincolales':0.09771,(((RS_GCF_00
0024205.1:0.26042,(RS_GCF_000215085.1:0.25436,RS_GCF_016907915.1:0.21949)100.0:0.03946)63
.0:0.02582,(GB_GCA_002365865.1:0.32039,((GB_GCA_016278505.1:0.26339,GB_GCA_000961575.1:
0.26084)100.0:0.05528,((GB_GCA_002402825.1:0.34031,RS_GCF_000429345.1:0.2634)100.0:0.0274
9,(RS_GCF_000376385.1:0.21831,GB_GCA_000961595.1:0.20949)100.0:0.04214)100.0:0.03028)78.0
:0.02184)32.0:0.02094)'100.0:o__Desulfotomaculales':0.03946,(GB_GCA_003604985.1:0.40669,(GB_
GCA_011048115.1:0.22446,(GB_GCA_017656185.1:0.20678,GB_GCA_001899445.1:0.27325)99.0:0.0
3981)98.0:0.04918)'100.0:o__Ammonifexales':0.07912)'100.0:c__Desulfotomaculia':0.05496)27.0:0.
02473,(GB_GCA_003054495.1:0.24388,RS_GCF_001950255.1:0.36823)53.0:0.05772)96.0:0.03018,(((
((RS_GCF_900129935.1:0.28596,GB_GCA_012521215.1:0.33702)'100.0:o__Desulfitobacteriales':0.17

621,RS_GCF_009720735.1:0.36282)'59.0:c__Desulfitobacteriia':0.03871,((GB_GCA_018434325.1:0.4
4542,(GB_GCA_012841935.1:0.4325,GB_GCA_018433015.1:0.24209)100.0:0.0493)'100.0:o__DRI-
13':0.08355,((RS_GCF_900176035.1:0.25315,GB_GCA_012720075.1:0.20326)96.0:0.037,(GB_GCA_0
09930175.1:0.36884,GB_GCA_910585405.1:0.36893)99.0:0.03783)'100.0:o__Peptococcales':0.08496
)'100.0:c__Peptococcia':0.04743)55.0:0.02555,((RS_GCF_002777255.1:0.23643,(RS_GCF_904420535.
1:0.42572,(GB_GCA_900760825.1:0.46514,GB_GCA_002398485.1:0.362)'100.0:o__UBA4068':0.0580
7)92.0:0.0378)'94.0:c__Dehalobacteriia':0.08323,(RS_GCF_001584725.1:0.49649,GB_GCA_00241876
5.1:0.40725)'100.0:c__TC1;o__TC1':0.1447)21.0:0.03061)13.0:0.02065,(((GB_GCA_013178045.1:0.19
113,GB_GCA_012519455.1:0.29891)'100.0:o__Ch66':0.05754,(GB_GCA_012522705.1:0.45422,(GB_G
CA_012729505.1:0.30185,GB_GCA_012799365.1:0.31234)82.0:0.03532)'100.0:o__Syntrophomonad
ales':0.18578)'57.0:c__Syntrophomonadia':0.04482,((((GB_GCA_012838615.1:0.22958,GB_GCA_012
840125.1:0.26681)'100.0:o__DULZ01':0.04438,GB_GCA_018333865.1:0.3581)73.0:0.02632,(((GB_GC
A_012523575.1:0.35038,GB_GCA_002352935.1:0.33529)37.0:0.03575,RS_GCF_002207765.1:0.1905
8)57.0:0.02668,(GB_GCA_017656305.1:0.25331,GB_GCA_001515945.1:0.37106)75.0:0.04229)53.0:0
.02634)47.0:0.02123,((RS_GCF_900176005.1:0.30011,(GB_GCA_014360065.1:0.31862,GB_GCA_014
360105.1:0.33313)73.0:0.04238)'62.0:o__Moorellales':0.03538,GB_GCA_003160265.1:0.5482)64.0:0
.04372)'60.0:c__Moorellia':0.02867)34.0:0.02426)22.0:0.02274)'92.0:p__Firmicutes_B':0.03723,(GB_
GCA_012840065.1:0.32465,(GB_GCA_002411145.1:0.24772,(((RS_GCF_000384135.1:0.24945,GB_GC
A_017554485.1:0.5383)'100.0:o__Acidaminococcales':0.08246,(GB_GCA_018058445.1:0.37427,((GB
_GCA_900155405.1:0.26545,GB_GCA_002297935.1:0.44991)100.0:0.09924,(RS_GCF_013393365.1:0
.34786,RS_GCF_000214495.1:0.42226)55.0:0.03584)'100.0:o__Veillonellales':0.12049)41.0:0.02806)
61.0:0.03328,(((((RS_GCF_900141835.1:0.18607,(GB_GCA_012523975.1:0.1445,(RS_GCF_900104455
.1:0.13628,(GB_GCA_017442105.1:0.2532,(GB_GCA_018052945.1:0.15929,(RS_GCF_000469545.1:0.
52575,RS_GCF_900086705.1:0.10767)99.0:0.04711)'99.0:o__Selenomonadales':0.04809)85.0:0.0293
1)47.0:0.03899)12.0:0.01296)26.0:0.02007,((GB_GCA_009929485.1:0.2383,GB_GCA_900550105.1:0.
19805)38.0:0.02497,GB_GCA_017861495.1:0.20063)43.0:0.02026)28.0:0.02229,((RS_GCF_90047637
5.1:0.14038,GB_GCA_017889625.1:0.21096)56.0:0.02108,(RS_GCF_004339805.1:0.17655,RS_GCF_0
03966895.1:0.16826)19.0:0.00782)'28.0:o__Sporomusales':0.0135)18.0:0.02209,RS_GCF_000219125
.1:0.20281)78.0:0.03218,GB_GCA_012798135.1:0.30482)82.0:0.04107)78.0:0.05467)'100.0:c__Negat
ivicutes':0.08709)'15.0:p__Firmicutes_C':0.03332)8.0:0.02091)13.0:0.02096)26.0:0.02905)21.0:0.024
44)13.0:0.02608,(GB_GCA_016190305.1:0.50733,((GB_GCA_003242505.1:0.21695,RS_GCF_0178743
15.1:0.25054)'100.0:c__Symbiobacteriia;o__Symbiobacteriales':0.16259,((GB_GCA_002375895.1:0.3
4073,(GB_GCA_012837735.1:0.30773,GB_GCA_002375925.1:0.23559)'100.0:o__UBA3575':0.07287)'
68.0:c__UBA3569':0.04383,(((GB_GCA_902809825.1:0.24772,GB_GCA_000237975.1:0.33905)'100.0:
c__Sulfobacillia;o__Sulfobacillales':0.28692,((GB_GCA_007133365.1:0.33467,GB_GCA_003563755.1:
0.34682)'100.0:c__SLMV01;o__SLMV01':0.27673,((GB_GCA_018729555.1:0.37848,GB_GCA_014896
375.1:0.22618)'100.0:c__RBS10-35;o__RBS10-
35':0.15926,(GB_GCA_017577485.1:0.29557,RS_GCF_000183545.2:0.33172)'100.0:c__Thermaeroba
cteria;o__Thermaerobacterales':0.07087)69.0:0.03681)73.0:0.0346)35.0:0.03378,((((GB_GCA_01489
6535.1:0.27906,GB_GCA_002391545.1:0.46577)'100.0:o__D8A-
2':0.23837,GB_GCA_007130145.1:0.42269)84.0:0.04423,(GB_GCA_012840015.1:0.3694,GB_GCA_01
8434225.1:0.3444)88.0:0.0699)81.0:0.04499,(((GB_GCA_014360055.1:0.35628,GB_GCA_002376005.
1:0.36646)22.0:0.04581,GB_GCA_012842575.1:0.41372)19.0:0.03151,(GB_GCA_902812875.1:0.4508
5,(GB_GCA_002291985.1:0.43994,GB_GCA_003530255.1:0.35499)99.0:0.07131)55.0:0.04547)19.0:0
.02716)'37.0:c__DTU015':0.02906)17.0:0.01683)23.0:0.02296)59.0:0.03302)'74.0:p__Firmicutes_E':0
.0303)45.0:0.06121,(((GB_GCA_005889495.1:0.62554,(GB_GCA_005880875.1:0.48611,(GB_GCA_013
044295.1:0.47128,GB_GCA_003162415.1:0.50177)'100.0:o__UBA8260':0.10287)100.0:0.06496)'100.
0:p__Dormibacterota;c__Dormibacteria':0.18727,(((GB_GCA_005881435.1:0.26564,GB_GCA_005881

885.1:0.42654)'100.0:o__P2-
11E':0.25394,(GB_GCA_013816405.1:0.41185,(((GB_GCA_903851225.1:0.0680805,bin.643:0.030697
):0.122454,bin.235:0.0969726):0.437335,GB_GCA_016191345.1:0.45024)'100.0:o__Limnocylindrales
':0.11818)100.0:0.15128)'100.0:c__Limnocylindria':0.09802,((((GB_GCA_011388535.1:0.40595,((GB_
GCA_903932805.1:0.5341,GB_GCA_012031225.1:0.33672)100.0:0.08429,GB_GCA_000526415.1:0.3
4537)'100.0:o__Chloroflexales':0.17616)100.0:0.06921,((GB_GCA_013390565.1:0.44792,(GB_GCA_0
03243865.1:0.3376,GB_GCA_015478675.1:0.32396)100.0:0.08055)'100.0:o__54-
19':0.06992,(GB_GCA_016781845.1:0.52978,(GB_GCA_902806415.1:0.38231,(GB_GCA_903864575.
1:0.56024,(GB_GCA_003577355.1:0.31895,GB_GCA_007133615.1:0.43433)100.0:0.05497)100.0:0.05
806)'100.0:o__Thermomicrobiales':0.09269)99.0:0.04822)100.0:0.03983)'100.0:c__Chloroflexia':0.05
711,(((((GB_GCA_002436065.1:0.33017,(GB_GCA_009692845.1:0.46914,(GB_GCA_016210245.1:0.2
2467,GB_GCA_019247515.1:0.44752)100.0:0.06955)100.0:0.07607)'100.0:c__UBA6077;o__UBA6077
':0.10156,(GB_GCA_016210125.1:0.42319,(((GB_GCA_011051445.1:0.24467,GB_GCA_004297775.1:
0.44691)75.0:0.0396,(GB_GCA_016190465.1:0.27144,GB_GCA_902810745.1:0.29193)91.0:0.05302)
46.0:0.02866,(GB_GCA_011334565.1:0.2696,GB_GCA_016189905.1:0.28044)100.0:0.06321)'80.0:o_
_FW602-bin22':0.03531)'100.0:c__FW602-
bin22':0.04952)74.0:0.02216,(((GB_GCA_016199325.1:0.31334,GB_GCA_011053995.1:0.37597)43.0:
0.05861,(GB_GCA_016190625.1:0.3078,GB_GCA_004297765.1:0.29929)44.0:0.05447)53.0:0.03056,(
(((GB_GCA_016190505.1:0.4261,(GB_GCA_016210205.1:0.34677,GB_GCA_016210485.1:0.34773)81.
0:0.05646)51.0:0.06019,(GB_GCA_016190815.1:0.33689,((((GB_GCA_003229915.1:0.30867,(GB_GC
A_018725455.1:0.171068,bin.164:0.214832):0.174832)'96.0:o__SZUA-
161':0.05931,GB_GCA_004376075.1:0.24024)18.0:0.03195,(((((((((GB_GCA_011192005.1:0.267721,
bin.356:0.206336):0.0242651,bin.366:0.114806):0.0606739,((GB_GCA_011191985.1:0.20141,GB_GC
A_002690375.1:0.23703)17.0:0.02434,((GB_GCA_018830745.1:0.24744,(((GB_GCA_011191875.1:0.1
2674,(GB_GCA_016783245.1:0.11003,(GB_GCA_004376105.1:0.08422,(GB_GCA_002418895.1:0.097
8924,bin.418:0.0422902):0.171038)67.0:0.02756)8.0:0.0128)9.0:0.01185,(GB_GCA_011192075.1:0.1
2947,GB_GCA_002688105.1:0.15714)19.0:0.01449)24.0:0.0104355,bin.672:0.193014):0.00937454)4.
0:0.01188,((GB_GCA_004375675.1:0.125579,bin.571:0.12202):0.0798009,GB_GCA_004376435.1:0.1
7968)17.0:0.02022)3.0:0.01454)28.0:0.02101)18.0:0.01655,((GB_GCA_011171075.1:0.0689333,bin.3
37:0.0590082):0.205477,GB_GCA_016202555.1:0.23395)90.0:0.02794)12.0:0.01709,(GB_GCA_0161
90925.1:0.22397,GB_GCA_016202545.1:0.21514)25.0:0.0422)10.0:0.02467,(GB_GCA_016210585.1:0
.27101,GB_GCA_016926695.1:0.42097)71.0:0.03496)39.0:0.02727,((GB_GCA_002382615.1:0.46803,
(GB_GCA_012719115.1:0.31037,(GB_GCA_003563865.1:0.41366,(RS_GCF_001889305.1:0.43011,GB
_GCA_009784845.1:0.43247)56.0:0.02972)26.0:0.02836)27.0:0.02928)71.0:0.03748,GB_GCA_00359
9745.1:0.33957)33.0:0.0277)'100.0:o__Dehalococcoidales':0.07238,(GB_GCA_018648325.1:0.37271,
GB_GCA_002347295.1:0.39649)32.0:0.07172)14.0:0.02728,(((GB_GCA_002352365.1:0.26052,(GB_G
CA_001795035.1:0.231112,bin.256:0.219978):0.107918)81.0:0.03473,GB_GCA_002367275.1:0.5891
9)57.0:0.05045,(((GB_GCA_011170325.1:0.20784,GB_GCA_003484395.1:0.38005)100.0:0.06766,GB_
GCA_003552125.1:0.70985)'100.0:o__GIF9':0.06696,((GB_GCA_016844565.1:0.30847,GB_GCA_0162
10565.1:0.34212)64.0:0.04608,GB_GCA_016189825.1:0.39621)32.0:0.0315)28.0:0.02201)3.0:0.0177
4)7.0:0.02766,(GB_GCA_002011475.1:0.29069,(GB_GCA_018825785.1:0.33924,GB_GCA_015661045
.1:0.32938)'98.0:o__JACVQG01':0.05651)34.0:0.03011)15.0:0.02615)66.0:0.03802,(GB_GCA_016210
705.1:0.29261,(GB_GCA_016190665.1:0.28001,(GB_GCA_016183425.1:0.28993,GB_GCA_01621042
5.1:0.31089)23.0:0.03706)13.0:0.02859)23.0:0.03199)18.0:0.02435)14.0:0.02378)16.0:0.03046,(GB_
GCA_016199375.1:0.45342,((GB_GCA_016200725.1:0.3967,(GB_GCA_009692825.1:0.37759,GB_GC
A_016190545.1:0.38388)94.0:0.08544)'90.0:o__SHYB01':0.05471,((GB_GCA_003228115.1:0.42746,((
(GB_GCA_016210665.1:0.33111,(((GB_GCA_009838465.1:0.38352,(GB_GCA_002717725.1:0.43434,(
(((GB_GCA_002722455.1:0.51637,GB_GCA_002719675.1:0.43609)66.0:0.06594,(GB_GCA_00939219

5.1:0.3869,GB_GCA_002715665.1:0.77311)42.0:0.09312)55.0:0.06443,GB_GCA_003228095.2:0.4565
)42.0:0.04993,(GB_GCA_002723415.1:0.46376,GB_GCA_002708065.1:0.46732)70.0:0.05561)27.0:0.
04118)64.0:0.04853)16.0:0.02893,(GB_GCA_016872515.1:0.29594,GB_GCA_016872355.1:0.32292)7
8.0:0.03566)'80.0:o__SAR202':0.08785,(((GB_GCA_012270805.1:0.52501,(GB_GCA_014239735.1:0.8
9605,(GB_GCA_002725925.1:0.166675,bin.216:0.0939413):0.232985)53.0:0.05758)64.0:0.07467,GB
_GCA_009693115.1:0.33249)70.0:0.05968,((GB_GCA_013204555.1:6.59058e-06,bin.490:6.11352e-
06):0.380558,bin.220:0.26478):0.0513556)'99.0:o__UBA1151':0.20985)94.0:0.04194)30.0:0.04287,(((
GB_GCA_002725515.1:0.64054,GB_GCA_016190445.1:0.28626)95.0:0.06617,(GB_GCA_002712585.
1:0.51788,(GB_GCA_012270525.1:0.35559,(GB_GCA_002723125.1:0.54674,(GB_GCA_002328185.1:
0.130388,bin.363:0.070688):0.315232)100.0:0.10346)80.0:0.04317)'100.0:o__UBA3495':0.1107)28.0
:0.02324,(GB_GCA_016202425.1:0.27045,((GB_GCA_003228195.2:0.32109,GB_GCA_009392515.1:0.
58481)73.0:0.08552,(GB_GCA_016210375.1:0.25453,GB_GCA_009692985.1:0.18189)36.0:0.03307)3
7.0:0.04298)'91.0:o__UBA6952':0.10133)44.0:0.0278)4.0:0.0217,(((GB_GCA_016179845.1:0.34486,((
GB_GCA_009692665.1:0.3924,GB_GCA_002311595.1:0.3724)92.0:0.04925,(GB_GCA_016210285.1:0
.24806,GB_GCA_002923335.1:0.36237)56.0:0.03178)90.0:0.04692)'97.0:o__UBA1127':0.04983,GB_
GCA_002717565.1:0.62036)85.0:0.03299,GB_GCA_015661155.1:0.40259)8.0:0.02072)7.0:0.02033)1
00.0:0.05266,((GB_GCA_009392005.1:0.43672,(GB_GCA_016210295.1:0.33533,GB_GCA_012271225
.1:0.44714)95.0:0.06575)'100.0:o__UBA6926':0.08687,GB_GCA_012270635.1:0.44777)35.0:0.04561)
20.0:0.02246)53.0:0.03085)20.0:0.02998)25.0:0.03152,(((((GB_GCA_015478665.1:0.34594,(GB_GCA
_009377745.1:0.38367,GB_GCA_003228685.1:0.34332)83.0:0.04846)'99.0:o__DSTF01':0.05829,(GB_
GCA_009693105.1:0.36943,GB_GCA_011057325.1:0.30578)65.0:0.0472)27.0:0.02708,(GB_GCA_009
377665.1:0.37971,(GB_GCA_016191435.1:0.60089,(GB_GCA_009838735.1:0.60151,GB_GCA_00270
7835.1:0.78134)91.0:0.04986)87.0:0.04103)35.0:0.03091)76.0:0.04086,(GB_GCA_009377715.1:0.313
99,(GB_GCA_001303545.1:0.23297,(GB_GCA_011047455.1:0.30715,GB_GCA_016178045.1:0.22219)
100.0:0.06962)69.0:0.03628)'100.0:o__SM23-28-
2':0.0612)99.0:0.08039,(GB_GCA_009693005.1:0.37378,(GB_GCA_009692945.1:0.3775,GB_GCA_01
6210175.1:0.25325)15.0:0.02507)'100.0:o__SHYD01':0.08385)30.0:0.02614)99.0:0.05516)'99.0:c__D
ehalococcoidia':0.04938)64.0:0.0217,(GB_GCA_016208345.1:0.27293,GB_GCA_011358165.1:0.2862
8)'100.0:c__DTOL01;o__DTOL01':0.10897)49.0:0.03076,(((((((GB_GCA_012689325.1:0.33278,GB_GC
A_011332765.1:0.40539)63.0:0.03564,(GB_GCA_014360825.1:0.23444,GB_GCA_002327925.1:0.396
47)'100.0:o__UBA2200':0.06286)36.0:0.02625,GB_GCA_011047295.1:0.35437)99.0:0.0425,((((GB_G
CA_014360855.1:0.26356,GB_GCA_011334325.1:0.23131)88.0:0.04967,(GB_GCA_011333425.1:0.30
231,GB_GCA_011364775.1:0.37144)63.0:0.04007)62.0:0.03068,(((GB_GCA_005223035.1:0.2786,GB_
GCA_016190785.1:0.43569)'100.0:o__UBA7937':0.09398,(GB_GCA_004377365.1:0.18092,(GB_GCA_
014360845.1:0.20983,GB_GCA_016875995.1:0.17703)41.0:0.04529)63.0:0.0356)18.0:0.03234,GB_G
CA_016929795.1:0.39099)8.0:0.02572)12.0:0.02317,(((((GB_GCA_902812335.1:0.27063,(((GB_GCA_
010092775.1:0.43215,((((GB_GCA_012031655.1:0.41538,GB_GCA_010092855.1:0.35062)100.0:0.04
266,GB_GCA_003695215.1:0.3739)67.0:0.02927,(GB_GCA_013178165.1:0.25941,GB_GCA_0167890
05.1:0.62499)59.0:0.02853)78.0:0.04515,GB_GCA_016927535.1:0.31581)100.0:0.05049)'100.0:o__A
ggregatilineales':0.06075,((GB_GCA_002455215.1:0.38877,GB_GCA_002346435.1:0.47427)100.0:0.0
787,((GB_GCA_016789245.1:0.34157,(GB_GCA_016200785.1:0.21161,GB_GCA_016875735.1:0.3076
7)63.0:0.03389)96.0:0.05768,(GB_GCA_016932475.1:0.4063,(GB_GCA_016927115.1:0.39424,((((GB_
GCA_013140605.1:0.373226,bin.439:0.158455):0.160524,GB_GCA_001899005.1:0.48063)99.0:0.041
27,((GB_GCA_016929095.1:0.3324,(GB_GCA_018648225.1:0.33853,(GB_GCA_013359445.1:0.28021,
GB_GCA_018662365.1:0.36198)91.0:0.03554)32.0:0.01575)47.0:0.0177,GB_GCA_011051635.1:0.230
87)69.0:0.0214)70.0:0.03129,GB_GCA_015484985.1:0.45667)100.0:0.05652)100.0:0.04187)75.0:0.03
701)75.0:0.03291)'100.0:o__Anaerolineales':0.07545)92.0:0.0299,(GB_GCA_013139935.1:0.3499,GB
_GCA_017993775.1:0.49376)'100.0:o__Promineofilales':0.0576)100.0:0.04001)44.0:0.02501,((GB_G

CA_016931085.1:0.33949,(GB_GCA_011048055.1:0.37478,GB_GCA_011373615.1:0.49662)56.0:0.04
154)'52.0:o__Thermoflexales':0.02962,(GB_GCA_015484715.1:0.22426,(GB_GCA_016926915.1:0.44
721,(GB_GCA_011192125.1:0.19948,GB_GCA_007123205.1:0.34364)100.0:0.05103)'100.0:o__B4-
G1':0.04955)85.0:0.04027)13.0:0.01803)98.0:0.04119,GB_GCA_016929775.1:0.42555)50.0:0.03125,(
GB_GCA_003130875.1:0.26392,(GB_GCA_002084875.1:0.45082,GB_GCA_011370265.1:0.29304)100
.0:0.08237)'100.0:o__4572-
78':0.10322)23.0:0.02663,(GB_GCA_013178015.1:0.4711,(GB_GCA_002366755.1:0.28003,(GB_GCA_
013152435.1:0.24309,(((GB_GCA_016719785.1:0.35329,(GB_GCA_014879765.1:0.33006,GB_GCA_0
02842085.1:0.34839)46.0:0.03801)57.0:0.03396,(GB_GCA_016713335.1:0.62305,GB_GCA_00369490
5.1:0.33555)63.0:0.03246)45.0:0.02718,(GB_GCA_015486235.1:0.34521,GB_GCA_013154135.1:0.37
131)91.0:0.04485)57.0:0.02353)31.0:0.02684)98.0:0.03947)21.0:0.02413)7.0:0.01722)16.0:0.03016)
21.0:0.01842,((GB_GCA_013151955.1:0.32359,GB_GCA_015487975.1:0.34306)'100.0:o__Ardenticat
enales':0.07078,(GB_GCA_009692745.1:0.38569,(GB_GCA_018263655.1:0.31336,GB_GCA_0167030
25.1:0.55448)72.0:0.04301)'91.0:o__UCB3':0.03999)78.0:0.0452)27.0:0.02193,(GB_GCA_016235055.
1:0.44797,(GB_GCA_016209205.1:0.29994,GB_GCA_016223105.1:0.30503)'100.0:o__UBA4142':0.17
518)98.0:0.06791)100.0:0.05854,GB_GCA_018823415.1:0.42486)'93.0:c__Anaerolineae':0.05272)92.
0:0.03094)96.0:0.04057,(((GB_GCA_012270555.1:0.43951,(GB_GCA_009840625.1:0.61295,GB_GCA_
002433065.1:0.55403)'100.0:o__UBA11872':0.15913)'100.0:c__UBA11872':0.11343,(GB_GCA_01105
3965.1:0.30299,GB_GCA_016199265.1:0.37652)'97.0:c__UBA2235':0.0555)92.0:0.06282,((GB_GCA_
013815535.1:0.39306,RS_GCF_016587435.1:0.50246)'100.0:c__Ktedonobacteria;o__Ktedonobactera
les':0.1724,(GB_GCA_002404055.1:0.48128,GB_GCA_002413265.1:0.52925)33.0:0.05618)32.0:0.041
43)21.0:0.02696)90.0:0.04317)'99.0:p__Chloroflexota':0.05601)99.0:0.08928,(((((GB_GCA_01251226
5.1:0.45912,(GB_GCA_016199525.1:0.22275,GB_GCA_005798285.1:0.37685)'100.0:o__SYMT01':0.1
8625)'88.0:c__SYMT01':0.06302,(GB_GCA_011334905.1:0.4021,(((GB_GCA_011053695.1:0.34959,(G
B_GCA_002254605.1:0.52301,(GB_GCA_003695835.1:0.44109,GB_GCA_002162075.1:0.64213)100.0
:0.1794)'100.0:c__Fimbriimonadia;o__Fimbriimonadales':0.09157)100.0:0.07155,((GB_GCA_903881
535.1:0.77992,GB_GCA_019239955.1:0.40626)'100.0:c__Armatimonadia;o__Armatimonadales':0.08
735,(GB_GCA_903859705.1:0.41564,GB_GCA_903951425.1:0.45129)'100.0:c__Chthonomonadetes;
o__Chthonomonadales':0.09682)96.0:0.05336)100.0:0.05119,((GB_GCA_019136435.1:0.47746,(GB_
GCA_011370285.1:0.32508,GB_GCA_011338025.1:0.42867)'100.0:o__UBA5829':0.06124)100.0:0.04
068,GB_GCA_017993055.1:0.38156)'99.0:c__UBA5829':0.05028)100.0:0.04937)50.0:0.02447)100.0:
0.07886,((GB_GCA_903928345.1:0.48362,(GB_GCA_011773575.1:0.42354,GB_GCA_011370625.1:0.
36653)'100.0:o__WVXJ01':0.08771)'100.0:c__CAIYQO01':0.10219,((GB_GCA_002305165.1:0.62665,(
GB_GCA_016869045.1:0.47841,(GB_GCA_003566775.1:0.41742,((GB_GCA_013314775.1:0.33341,(G
B_GCA_013178155.1:0.30619,GB_GCA_012518355.1:0.3896)100.0:0.06997)62.0:0.04232,(GB_GCA_
012523355.1:0.41259,GB_GCA_003170595.1:0.49459)49.0:0.07243)62.0:0.04296)100.0:0.08285)'10
0.0:o__UBA5377':0.13524)'100.0:c__UBA5377':0.07681,((GB_GCA_012961305.1:0.28553,GB_GCA_0
16183835.1:0.52233)'100.0:c__UBA10988;o__UBA10988':0.19341,((GB_GCA_011380295.1:0.31352,
GB_GCA_011335125.1:0.36454)'100.0:c__HRBIN17;o__HRBIN17':0.22653,(GB_GCA_002779825.1:0.
453,(GB_GCA_004172935.1:0.71729,GB_GCA_001872605.1:0.43305)63.0:0.05676)'100.0:c__Abditib
acteria':0.07332)88.0:0.07276)43.0:0.03094)79.0:0.03624)42.0:0.03172)'60.0:p__Armatimonadota':0
.05491,((GB_GCA_016868955.1:0.29278,GB_GCA_011053555.1:0.23673)100.0:0.11587,((GB_GCA_0
16210785.1:0.24104,GB_GCA_011057335.1:0.20272)100.0:0.06489,(GB_GCA_005882445.1:0.35455,
(GB_GCA_005888595.1:0.3302,GB_GCA_011047475.1:0.31976)100.0:0.04618)67.0:0.03561)57.0:0.0
3108)'100.0:p__CSP1-3;c__CSP1-3;o__CSP1-
3':0.22474)37.0:0.02586,(((GB_GCA_003244105.1:0.5376,(GB_GCA_004298675.1:0.26219,GB_GCA_
003134035.1:0.38116)'100.0:o__Baltobacterales':0.15446)'100.0:c__Eremiobacteria':0.25904,(GB_G
CA_011343225.1:0.67042,((GB_GCA_016183885.1:0.47448,(GB_GCA_016183865.1:0.37339,GB_GCA

_013286945.1:0.47422)86.0:0.06324)65.0:0.06399,(GB_GCA_902791745.1:0.64472,(GB_GCA_01686
8975.1:0.41591,(GB_GCA_018266105.1:0.44689,GB_GCA_017444045.1:0.52579)'100.0:o__Xenobial
es':0.12953)100.0:0.07161)'56.0:c__Xenobia':0.04866)89.0:0.05115)90.0:0.04711)'94.0:p__Eremioba
cterota':0.05097,GB_GCA_002410925.1:0.50655)32.0:0.03004)43.0:0.0266)15.0:0.02872)9.0:0.0332
5,((GB_GCA_016199255.1:0.42982,((RS_GCF_014201675.1:0.27968,RS_GCF_014647535.1:0.34832)1
00.0:0.14999,(GB_GCA_902806425.1:0.4327,(GB_GCA_013298185.1:0.3766,RS_GCF_003966215.1:0.
51393)100.0:0.12557)97.0:0.05949)'100.0:o__Deinococcales':0.14862)'100.0:p__Deinococcota;c__D
einococci':0.28424,(((((GB_GCA_902615855.1:1.02808,((GB_GCA_002898115.1:0.43933,((GB_GCA_0
16870535.1:0.256837,bin.66:0.175687):0.111223,GB_GCA_013043085.1:0.42063)57.0:0.02768)82.0:
0.02973,(((GB_GCA_015488325.1:0.16776,GB_GCA_002238785.1:0.41452)75.0:0.04682,GB_GCA_01
3812505.1:0.35257)100.0:0.06953,((GB_GCA_016650365.1:0.32006,GB_GCA_009841105.1:0.4513)3
0.0:0.03212,GB_GCA_013152275.1:0.28754)55.0:0.02634)99.0:0.0462)'100.0:o__UBA5794':0.08545)
100.0:0.16393,((((((GB_GCA_003156595.1:0.36258,(GB_GCA_002861595.1:0.20681,((GB_GCA_0138
16725.1:0.22882,GB_GCA_902805655.1:0.26609)20.0:0.02853,GB_GCA_019243975.1:0.16772)11.0:
0.02355)13.0:0.01898)9.0:0.0145,(GB_GCA_005884125.1:0.20302,(((GB_GCA_003453695.1:0.25488,
GB_GCA_009701115.1:0.68222)100.0:0.06975,((GB_GCA_004298555.1:0.41354,GB_GCA_01304446
5.1:0.40087)98.0:0.06586,GB_GCA_003166375.1:0.3442)46.0:0.03033)100.0:0.0426,GB_GCA_00324
4275.1:0.25028)68.0:0.03169)18.0:0.01714)83.0:0.04244,(GB_GCA_005888395.1:0.16829,GB_GCA_
019243595.1:0.21311)100.0:0.03676)86.0:0.03113,((GB_GCA_004366205.1:0.16936,GB_GCA_01678
1105.1:0.2584)100.0:0.04306,((GB_GCA_002724355.1:0.51672,((GB_GCA_013694495.1:0.29534,GB_
GCA_016185275.1:0.27425)96.0:0.02752,((RS_GCF_009650215.1:0.23233,((GB_GCA_016462055.1:0
.00601984,bin.145:0.00700328):0.31351,GB_GCA_016794585.1:0.20714)41.0:0.02347)68.0:0.02455,
(((GB_GCA_013003325.1:0.51579,((GB_GCA_905479685.1:0.33517,(GB_GCA_009843735.1:0.36127,
GB_GCA_002299135.1:0.43045)49.0:0.02721)28.0:0.02384,(GB_GCA_009835395.1:0.40748,(GB_GC
A_003485885.1:0.33665,GB_GCA_002713605.1:0.51376)99.0:0.05339)60.0:0.03785)41.0:0.02137)19
.0:0.02698,GB_GCA_903852755.1:0.49747)12.0:0.01847,(GB_GCA_016870475.1:0.30615,GB_GCA_0
09699605.1:0.25391)36.0:0.04404)41.0:0.02489)24.0:0.02241)30.0:0.02173)70.0:0.03212,((((((RS_G
CF_000348785.1:0.258444,bin.451:0.229062):0.00190269,bin.158:0.273089):0.00540349,bin.611:0.2
71308):0.00333659,bin.260:0.243453):0.00314923,bin.381:0.259244):0.222104,GB_GCA_016716005
.1:0.18162)64.0:0.03961)72.0:0.02621)85.0:0.03789)'100.0:o__Acidimicrobiales':0.10441,(((GB_GCA
_017882905.1:0.3569,(GB_GCA_017882925.1:0.23046,((GB_GCA_016870595.1:0.213227,bin.313:0.2
5905):0.0761632,GB_GCA_016870395.1:0.24776)40.0:0.01976)87.0:0.02348)98.0:0.03844,GB_GCA_
018057765.1:0.65008)60.0:0.04005,(GB_GCA_009886115.1:0.26188,(GB_GCA_018003035.1:0.3837,
GB_GCA_016179865.1:0.23571)81.0:0.05077)63.0:0.03922)'86.0:o__IMCC26256':0.05771)100.0:0.1
1046,(GB_GCA_002352645.1:0.5345,GB_GCA_003577105.1:0.41493)79.0:0.06651)86.0:0.04363)'100
.0:c__Acidimicrobiia':0.05656,(((GB_GCA_003565155.1:0.57797,(RS_GCF_003073135.1:0.33526,GB_
GCA_009379795.1:0.32088)'100.0:o__Euzebyales':0.10671)100.0:0.09535,((((((GB_GCA_002430405.
1:0.177283,bin.546:0.113253):0.214717,(GB_GCA_018969715.1:0.391836,bin.149:0.289494):0.1106
34)99.0:0.03952,(((GB_GCA_004297305.1:0.17865,GB_GCA_903825575.1:0.2204)48.0:0.03456,(GB_
GCA_009699025.1:0.23946,((GB_GCA_009700485.1:0.219112,bin.403:0.204953):0.00737091,bin.84:
0.115348):0.137407)22.0:0.0169)90.0:0.02201,(GB_GCA_002699445.1:0.34406,(GB_GCA_00345265
5.1:0.113409,bin.347:0.122705):0.164461)90.0:0.0249)40.0:0.01715)100.0:0.04189,((((GB_GCA_002
284855.1:0.185528,bin.171:0.105239):0.0573453,bin.494:0.176163):0.0424747,bin.244:0.177668):0.
321642,GB_GCA_003529305.1:0.27413)47.0:0.03875)'97.0:o__Nanopelagicales':0.03955,(((GB_GCA
_013698395.1:0.14819,GB_GCA_013696235.1:0.22519)63.0:0.05027,(((RS_GCF_000015025.1:0.2830
9,(GB_GCA_009379955.1:0.2844,RS_GCF_900099965.1:0.42321)'85.0:o__Streptosporangiales':0.026
81)46.0:0.01664,((((GB_GCA_903970495.1:0.28172,RS_GCF_000374165.1:0.30192)67.0:0.03128,GB_
GCA_016650445.1:0.22162)56.0:0.0227,(GB_GCA_017882835.1:0.16627,(GB_GCA_019239875.1:0.2

5253,GB_GCA_017882785.1:0.20049)52.0:0.03742)49.0:0.02156)83.0:0.03312,(((RS_GCF_00042144
5.1:0.28915,(RS_GCF_004382795.1:0.34936,((RS_GCF_003350445.1:0.32779,RS_GCF_004011835.1:0
.37856)100.0:0.04181,RS_GCF_009707545.1:0.30194)100.0:0.0623)39.0:0.02858)41.0:0.0251,((GB_
GCA_003244095.1:0.17872,GB_GCA_902805565.1:0.19789)46.0:0.02519,(GB_GCA_003244255.1:0.2
2117,RS_GCF_004571075.1:0.31982)61.0:0.01976)27.0:0.01918)17.0:0.01912,(RS_GCF_003002955.1
:0.55667,((RS_GCF_000585375.1:0.16027,GB_GCA_003243955.1:0.30114)57.0:0.02263,GB_GCA_01
0993775.1:0.23261)69.0:0.02815)100.0:0.05276)82.0:0.0482)'76.0:o__Mycobacteriales':0.03474)81.
0:0.02339,((RS_GCF_000384115.1:0.21328,RS_GCF_003634695.1:0.2267)51.0:0.01909,(RS_GCF_001
543895.1:0.12497,(RS_GCF_018139695.1:0.25858,RS_GCF_003143855.1:0.40818)100.0:0.03038)'10
0.0:o__Streptomycetales':0.03757)56.0:0.01931)26.0:0.01481)24.0:0.01568,(RS_GCF_900106035.1:0
.29235,(((RS_GCF_004361855.1:0.18324,GB_GCA_009787035.1:0.66354)90.0:0.03696,RS_GCF_0134
08415.1:0.35102)90.0:0.03821,RS_GCF_016907715.1:0.21994)'90.0:o__Propionibacteriales':0.038)6
4.0:0.02973)60.0:0.0206)77.0:0.02843,((GB_GCA_014377795.1:0.20221,GB_GCA_012729695.1:0.39
806)89.0:0.04035,((GB_GCA_002748415.1:0.28838,((RS_GCF_001584615.1:0.47618,(((RS_GCF_0044
02375.1:0.212612,bin.662:0.131939):0.0552202,bin.30:0.253996):0.266058,RS_GCF_001281315.1:0.
4093)69.0:0.03129)77.0:0.04442,((RS_GCF_003130585.1:0.39893,RS_GCF_003315615.1:0.6527)48.0
:0.04155,(((RS_GCF_001907245.1:0.55673,RS_GCF_009733845.1:0.25876)99.0:0.0374,RS_GCF_0098
29685.1:0.35542)86.0:0.02878,RS_GCF_000759715.1:0.39783)74.0:0.02786)67.0:0.03379)85.0:0.035
39)13.0:0.01903,((RS_GCF_003002055.1:0.27459,RS_GCF_003751265.1:0.24656)77.0:0.03126,RS_G
CF_002198675.1:0.19113)61.0:0.02668)25.0:0.01591)'87.0:o__Actinomycetales':0.07994)100.0:0.25
484)'95.0:c__Actinomycetia':0.06657,((GB_GCA_902812355.1:0.19174,GB_GCA_013817045.1:0.277
09)'100.0:o__CADDZG01':0.22384,((GB_GCA_005885015.1:0.32902,(GB_GCA_016210805.1:0.19658,
GB_GCA_016201135.1:0.30257)95.0:0.04465)'100.0:o__UBA4738':0.20359,GB_GCA_005883175.1:0.
41747)84.0:0.04896)'100.0:c__UBA4738':0.05797)87.0:0.03598)100.0:0.12095,(GB_GCA_902806095
.1:0.66127,((GB_GCA_016235065.1:0.45377,(GB_GCA_012514635.1:0.47739,GB_GCA_903927755.1:
0.52489)40.0:0.03911)85.0:0.04792,((GB_GCA_017882555.1:0.57021,(GB_GCA_903939845.1:0.4725
7,(GB_GCA_016463365.1:0.45935,(GB_GCA_002898855.1:0.38006,GB_GCA_005788585.1:0.37627)4
1.0:0.02792)99.0:0.04088)'100.0:o__Solirubrobacterales':0.1551)47.0:0.03888,((GB_GCA_013817555
.1:0.58107,GB_GCA_009885805.1:0.42226)85.0:0.05425,GB_GCA_007122215.1:0.53794)'97.0:o__G
aiellales':0.05361)100.0:0.0726)'100.0:c__Thermoleophilia':0.10137)100.0:0.0921)99.0:0.05091,((((((
GB_GCA_002371495.1:0.46116,(GB_GCA_009779015.1:0.40745,GB_GCA_017515355.1:0.34606)88.
0:0.03324)80.0:0.02988,(GB_GCA_900314665.1:0.27321,GB_GCA_013335845.1:0.2652)89.0:0.03595
)47.0:0.0223,(((GB_GCA_900768265.1:0.34192,RS_GCF_001494635.1:0.36086)100.0:0.07724,GB_GC
A_900555105.1:0.34214)100.0:0.11819,GB_GCA_900758075.1:0.33297)51.0:0.03423)'100.0:o__Cori
obacteriales':0.11693,((GB_GCA_016649875.1:0.25657,GB_GCA_009777935.1:0.33084)69.0:0.0351,
GB_GCA_009781115.1:0.34611)'100.0:o__WRKU01':0.11401)33.0:0.03731,((((GB_GCA_014859305.1
:0.20695,GB_GCA_005774595.1:0.25502)34.0:0.02882,(GB_GCA_016938395.1:0.3068,GB_GCA_903
831865.1:0.3018)42.0:0.02153)99.0:0.04038,(GB_GCA_002367305.1:0.21159,((GB_GCA_016841065.
1:0.19948,GB_GCA_002293965.1:0.31308)50.0:0.02202,GB_GCA_018333995.1:0.24625)99.0:0.0255
9)52.0:0.02178)98.0:0.0421,(GB_GCA_016213085.1:0.17911,(GB_GCA_009779675.1:0.26095,GB_GC
A_013334745.1:0.18406)97.0:0.02792)71.0:0.02924)'98.0:o__OPB41':0.05447)'100.0:c__Coriobacter
iia':0.28616,(((GB_GCA_015711695.1:0.25147,GB_GCA_018894655.1:0.32321)'100.0:o__Fen-
727':0.23101,(GB_GCA_015711725.1:0.28034,GB_GCA_015711955.1:0.29382)'100.0:o__Geothermin
colales':0.15737)'100.0:c__Geothermincolia':0.08072,((RS_GCF_013281975.1:0.39541,(GB_GCA_018
830725.1:0.49254,(GB_GCA_018894445.1:0.0219057,bin.63:0.0224628):0.594634)63.0:0.06726)'100
.0:c__Humimicrobiia':0.12244,(GB_GCA_003599235.1:0.46789,(GB_GCA_902810715.1:0.39429,((GB
_GCA_011046085.1:0.31174,GB_GCA_016208465.1:0.30743)'92.0:o__UBA3085':0.05397,((GB_GCA_
003491635.1:0.23096,GB_GCA_002339355.1:0.20453)'96.0:o__Subteraquimicrobiales':0.05779,GB_

GCA_015711795.1:0.38285)45.0:0:0.03512)62.0:0:0.03435)74.0:0:0.04222)'98.0:c__Aquicultoria':0.05601)
97.0:0:0.05109)93.0:0:0.04475)77.0:0:0.03575)'100.0:p__Actinobacteriota':0.06527)27.0:0:0.03893)3.0:0:0.05
651)4.0:0:0.03716)11.0:0:0.03055,((((GB_GCA_018056825.1:0.34829,RS_GCF_015775515.1:0.42289)'10
0.0:c__Atribacteria;o__Atribacterales':0.38239,(GB_GCA_012520575.1:0.44368,(GB_GCA_01881966
5.1:0.0575476,bin.538:0.0599603):0.284292)'100.0:c__JS1;o__SB-
45':0.20794)'94.0:p__Atribacterota':0.07241,((GB_GCA_003648815.1:0.22384,GB_GCA_005223095.
1:0.34923)97.0:0:0.05755,((GB_GCA_011372105.1:0.29322,bin.395:0.255538):0.0512698,GB_GCA_00
0402295.1:0.31707)46.0:0:0.03864)'100.0:p__Aerophobota;c__Aerophobia;o__Aerophobales':0.32878
)60.0:0:0.06455,((((((GB_GCA_016934855.1:0.30844,(GB_GCA_003557485.1:0.40812,GB_GCA_003553
445.1:0.35881)79.0:0:0.0527)'100.0:c__SLGR01;o__SLGR01':0.33005,(GB_GCA_003650255.1:0.54513,
GB_GCA_013791675.1:0.75644)'100.0:c__CG03':0.14724)'100.0:p__CG03':0.10071,(GB_GCA_90032
1865.1:0.80351,(((GB_GCA_002069855.1:0.33306,GB_GCA_002780205.1:0.25725)'100.0:c__UBA891
9;o__UBA8919':0.29685,(GB_GCA_002412545.1:0.45669,(((GB_GCA_016207685.1:0.77512,(GB_GCA
_016204975.1:0.49424,((GB_GCA_001871115.1:0.64046,(GB_GCA_012523635.1:0.36844,GB_GCA_9
10576915.1:0.59623)100.0:0:0.09156)'100.0:o__Elusimicrobiales':0.14437,(GB_GCA_016205045.1:0.3
6018,((GB_GCA_016194625.1:0.51871,(GB_GCA_016212845.1:0.32445,GB_GCA_016212885.1:0.363
44)86.0:0:0.03491)'100.0:o__UBA1565':0.08651,GB_GCA_001800185.1:0.44463)44.0:0:0.03084)56.0:0:0.0
5038)73.0:0:0.06382)90.0:0:0.0523)100.0:0:0.10685,(GB_GCA_001800085.1:0.55793,((GB_GCA_01619678
5.1:0.37435,GB_GCA_001800075.1:0.37371)'100.0:o__2-01-FULL-59-
12':0.09528,(GB_GCA_002176905.1:0.51419,GB_GCA_016706425.1:0.53746)'100.0:o__F11':0.08701
)52.0:0:0.03268)88.0:0:0.0482)'100.0:c__Elusimicrobia':0.07279,((GB_GCA_011329045.1:0.67798,(GB_G
CA_018830385.1:0.39131,(GB_GCA_001871125.1:0.31119,GB_GCA_001800495.1:0.46358)73.0:0:0.04
906)'100.0:o__CG1-02-37-
114':0.06737)63.0:0:0.08356,((GB_GCA_018433585.1:0.46936,(GB_GCA_016929635.1:0.26034,(GB_G
CA_001800315.1:0.28485,(GB_GCA_016930715.1:0.2323,GB_GCA_903880015.1:0.27867)31.0:0:0.034
46)46.0:0:0.03297)44.0:0:0.03849)'100.0:o__Endomicrobiales':0.09453,(GB_GCA_011338245.1:0.45793,
GB_GCA_002841695.1:0.54336)'99.0:o__PHAN01':0.09451)99.0:0:0.05178)'59.0:c__Endomicrobia':0.0
4292)60.0:0:0.03643)45.0:0:0.03178)'75.0:p__Elusimicrobiota':0.05981,(GB_GCA_009619095.1:0.39897,
GB_GCA_003153935.1:0.45641)'100.0:p__UBA6262;c__UBA6262':0.06207)42.0:0:0.02663)54.0:0:0.0287
)74.0:0:0.08205,((GB_GCA_003152075.1:0.53772,GB_GCA_013335425.1:0.56115)'70.0:p__FCPU426':0
.09655,((GB_GCA_903915125.1:0.29812,GB_GCA_001778355.1:0.33025)'100.0:p__Firestonebacteria
;c__D2-FULL-39-29;o__D2-FULL-39-
29':0.26091,(GB_GCA_903837445.1:0.30601,(GB_GCA_018000835.1:0.33074,(GB_GCA_003159495.
1:0.29564,(GB_GCA_011049465.1:0.29102,GB_GCA_016937715.1:0.28369)84.0:0:0.05687)81.0:0:0.0453
1)68.0:0:0.05679)'100.0:p__Goldbacteria;c__PGYV01;o__PGYV01':0.34821)56.0:0:0.05696)94.0:0:0.05291)
38.0:0:0.02709,((((((GB_GCA_005239945.1:0.31544,GB_GCA_016208205.1:0.40999)'100.0:c__CG2-30-
40-21;o__CG2-30-40-
21':0.07402,GB_GCA_003498085.1:0.6148)50.0:0:0.04126,GB_GCA_018830565.1:0.47613)55.0:0:0.0450
5,GB_GCA_003485015.1:0.47148)'100.0:p__UBA9089':0.09418,(GB_GCA_018903055.1:0.42086,(GB
_GCA_001871015.1:0.2254,GB_GCA_011389435.1:0.23173)'100.0:p__Desantisbacteria;c__UBA1551
;o__UBA1551':0.29807)64.0:0:0.05756)11.0:0:0.02581,GB_GCA_016209155.1:0.55224)27.0:0:0.03753)16.
0:0:0.03914,(((((((GB_GCA_003551625.1:0.3494,GB_GCA_003561965.1:0.35175)'100.0:p__PUNC01;c_
_PUNC01;o__PUNC01':0.2582,(GB_GCA_012729785.1:0.48394,GB_GCA_016933315.1:0.59044)30.0:
0.0639)41.0:0:0.04034,(GB_GCA_016931415.1:0.41949,(GB_GCA_013203085.1:5.84488e-
06,bin.280:6.11352e-
06):0.383044)'100.0:p__JABMQX01;c__JABMQX01;o__JABMQX01':0.22463)42.0:0:0.03831,(GB_GCA_
018435605.1:0.5658,(GB_GCA_016178055.1:0.52213,(GB_GCA_016929035.1:0.4767,GB_GCA_0169
32965.1:0.60192)'100.0:p__JAFGBW01;c__JAFGBW01':0.14921)60.0:0:0.04494)63.0:0:0.0469)73.0:0:0.039

09,((((GB_GCA_002392285.1:0.74669,((((((GB_GCA_002922725.1:0.3968,GB_GCA_010500615.1:0.44
848)100.0:0.05043,(GB_GCA_014529615.1:0.4041,(GB_GCA_003566375.1:0.32548,(RS_GCF_900104
925.1:0.290915,bin.295:0.159047):0.272525)100.0:0.055)100.0:0.04464)88.0:0.0245,((RS_GCF_0142
30055.1:0.34324,GB_GCA_016933595.1:0.38433)97.0:0.07152,((GB_GCA_018668635.1:0.37019,GB_
GCA_002716045.1:0.37735)100.0:0.07249,(GB_GCA_013215165.1:0.35839,GB_GCA_002699365.1:0.
43961)71.0:0.03324)71.0:0.02523)100.0:0.03781)98.0:0.03006,((GB_GCA_002313355.1:0.31164,(GB
_GCA_002376875.1:0.27048,GB_GCA_001872735.1:0.39074)68.0:0.05063)40.0:0.03574,(GB_GCA_0
13285985.1:0.37066,RS_GCF_014230085.1:0.27856)38.0:0.03835)13.0:0.02586)19.0:0.03641,(GB_G
CA_900545715.1:0.52169,(GB_GCA_903850885.1:0.0796884,bin.332:0.0723447):0.496152)67.0:0.05
359)65.0:0.04313,(GB_GCA_001831055.1:0.35213,GB_GCA_001831095.1:0.3585)88.0:0.07884)84.0:
0.06316)'100.0:o__Opitutales':0.2734,((GB_GCA_016198745.1:0.50391,(((GB_GCA_016871715.1:0.2
7029,((((GB_GCA_016795425.1:0.27541,GB_GCA_005789375.1:0.36312)92.0:0.03954,(GB_GCA_016
871495.1:0.20234,GB_GCA_005805305.1:0.22044)89.0:0.05234)8.0:0.02007,(GB_GCA_018610125.1:
0.38496,(GB_GCA_002348345.1:0.33541,(GB_GCA_002715965.1:0.32305,(GB_GCA_003972865.1:0.
38479,GB_GCA_011523245.1:0.42238)78.0:0.02994)78.0:0.03268)49.0:0.02239)20.0:0.03161)1.0:0.0
183,(((GB_GCA_011368065.1:0.27393,(GB_GCA_016935555.1:0.19643,GB_GCA_011337995.1:0.220
11)53.0:0.04605)18.0:0.0245,(GB_GCA_016124235.1:0.27115,(((GB_GCA_009695285.1:0.13956,GB_
GCA_016219875.1:0.16806)65.0:0.04184,(GB_GCA_016713365.1:0.16282,GB_GCA_003452185.1:0.2
4917)6.0:0.02541)5.0:0.02013,(((GB_GCA_016195385.1:0.17641,((GB_GCA_016199935.1:0.21362,(G
B_GCA_016871675.1:0.20806,GB_GCA_009773355.1:0.19182)85.0:0.04051)48.0:0.02583,(GB_GCA_
016235585.1:0.15428,(GB_GCA_009691645.1:0.18481,GB_GCA_014193395.1:0.1836)34.0:0.02161)2
5.0:0.02535)52.0:0.02582)27.0:0.02207,((GB_GCA_003219675.1:0.1608,GB_GCA_009695265.1:0.18
148)90.0:0.02617,(GB_GCA_009695275.1:0.18648,GB_GCA_008933045.1:0.23803)43.0:0.0366)38.0:
0.02245)2.0:0.01672,((GB_GCA_903927905.1:0.18368,GB_GCA_013820875.1:0.17444)97.0:0.03733,
((GB_GCA_016200005.1:0.14766,RS_GCF_000172555.1:0.15729)47.0:0.01783,((GB_GCA_002385705
.1:0.16696,GB_GCA_002298785.1:0.1971)67.0:0.02217,((GB_GCA_903870815.1:0.29188,(GB_GCA_9
03873415.1:0.14566,GB_GCA_017853785.1:0.15406)52.0:0.02524)53.0:0.01337,GB_GCA_01136760
5.1:0.2306)19.0:0.02426)28.0:0.02549)7.0:0.01718)4.0:0.01531)0.0:0.01648)0.0:0.016)1.0:0.01356)1
.0:0.01895,(GB_GCA_018006935.1:0.29029,(GB_GCA_017880715.1:0.18074,GB_GCA_002305615.1:
0.41593)7.0:0.035)11.0:0.04308)0.0:0.01405)1.0:0.0233)2.0:0.02963,((GB_GCA_011333365.1:0.14,G
B_GCA_011358515.1:0.33198)73.0:0.07299,GB_GCA_002070715.1:0.2215)38.0:0.03737)'100.0:o__P
edosphaerales':0.19415,(GB_GCA_903870275.1:0.36559,(GB_GCA_003152315.1:0.194421,bin.408:0.
21254):0.253369)'99.0:o__Palsa-
1439':0.06712)84.0:0.04576)51.0:0.03928,(((GB_GCA_010025475.1:0.42131,GB_GCA_012031345.1:
0.35608)100.0:0.09335,((GB_GCA_903889845.1:0.25855,RS_GCF_000019665.1:0.62483)100.0:0.065
04,GB_GCA_003054695.1:0.35453)100.0:0.06607)'100.0:o__Methylacidiphilales':0.10783,(((GB_GCA
_019243005.1:0.32317,((GB_GCA_009885995.1:0.235257,bin.670:0.159689):0.143983,GB_GCA_003
217875.1:0.44165)35.0:0.02079)86.0:0.02931,(((GB_GCA_017853515.1:0.0773125,bin.309:0.079106
5):0.0142878,bin.507:0.0833995):0.28688,(GB_GCA_903831635.1:0.34731,(GB_GCA_003176035.1:0
.28368,(GB_GCA_015655545.1:0.1841,(GB_GCA_018970255.1:0.263883,bin.119:0.165347):0.064076
5)70.0:0.02618)100.0:0.03595)100.0:0.0463)100.0:0.05088)'100.0:o__Chthoniobacterales':0.05634,((
(GB_GCA_007125835.1:0.39784,RS_GCF_014904755.1:0.40559)99.0:0.04585,GB_GCA_902589825.1
:0.43519)100.0:0.03527,(GB_GCA_903954065.1:0.37871,(GB_GCA_000739655.1:0.37606,GB_GCA_0
07123195.1:0.32909)94.0:0.04414)79.0:0.02412)'100.0:o__Verrucomicrobiales':0.13064)100.0:0.109
11)100.0:0.06977)100.0:0.06622)'100.0:c__Verrucomicrobiae':0.09035,((((GB_GCA_017424105.1:0.5
1921,((GB_GCA_903861045.1:0.30694,(GB_GCA_903935195.1:0.22817,bin.39:0.202305):0.1442)82.
0:0.03731,GB_GCA_017962605.1:0.48348)100.0:0.10923)'100.0:o__RFP12':0.15449,(((((GB_GCA_00
7134465.1:0.40102,GB_GCA_001803315.1:0.36892)39.0:0.06292,(GB_GCA_003565095.1:0.39188,((

GB_GCA_016783565.1:0.31779,(GB_GCA_018645275.1:0.109164,bin.178:0.173477):0.189596)66.0:
0.04525,(GB_GCA_018648805.1:0.119801,bin.508:0.114311):0.239809)36.0:0.03026)19.0:0.03566)4.
0:0.02334,(GB_GCA_016873655.1:0.36684,(GB_GCA_016934225.1:0.29571,GB_GCA_001804885.1:0
.28081)45.0:0.05193)15.0:0.03764)16.0:0.02355,(GB_GCA_011367825.1:0.41063,(GB_GCA_0168736
35.1:0.26764,GB_GCA_016929365.1:0.2684)92.0:0.0514)39.0:0.04961)'78.0:o__UBA8416':0.04232,(
GB_GCA_004525935.1:0.41395,((GB_GCA_016929905.1:0.36455,((GB_GCA_018400335.1:0.32516,G
B_GCA_018898455.1:0.26774)100.0:0.04181,(GB_GCA_012514555.1:0.31165,GB_GCA_016199105.1
:0.28184)100.0:0.05212)'100.0:o__JAAZAB01':0.1236)53.0:0.04001,(GB_GCA_003567575.1:0.47427,(
GB_GCA_903828035.1:0.38227,(GB_GCA_903824675.1:0.33026,(GB_GCA_903925685.1:0.31878,GB
_GCA_903958395.1:0.34072)100.0:0.05699)62.0:0.04032)'94.0:o__CAIKKV01':0.04313)40.0:0.033)34
.0:0.01874)77.0:0.03)67.0:0.05559)100.0:0.07387,((((GB_GCA_007132345.1:0.37328,(GB_GCA_0071
25875.1:0.42681,(GB_GCA_012103575.1:0.30854,GB_GCA_018814445.1:0.27327)86.0:0.04869)'92.0
:o__SLAD01':0.03413)37.0:0.0322,(((GB_GCA_003563855.1:0.32993,GB_GCA_016873715.1:0.33119)
'100.0:o__PWTM01':0.10222,(((GB_GCA_001804865.1:0.24403,GB_GCA_018001225.1:0.2815)82.0:0
.05074,(GB_GCA_903871765.1:0.30589,GB_GCA_903906425.1:0.35625)60.0:0.04476)45.0:0.03113,
GB_GCA_002304915.1:0.52411)'88.0:o__LD1-
PB3':0.04958)49.0:0.03394,GB_GCA_012961745.1:0.50739)31.0:0.02262)28.0:0.02565,((RS_GCF_00
1017655.1:0.37084,(RS_GCF_900890425.1:0.295237,bin.671:0.27852):0.126913)'100.0:o__Kiritimati
ellales':0.09562,(GB_GCA_009929845.1:0.2524,GB_GCA_009930475.1:0.36212)'100.0:o__RZYC01':0.
09271)75.0:0.04901)26.0:0.02776,(GB_GCA_003566475.1:0.43806,GB_GCA_003565035.1:0.49273)'8
8.0:o__SS1-B-03-
39':0.09109)79.0:0.04525)'100.0:c__Kiritimatiellae':0.14369,(((GB_GCA_001803205.1:0.43609,((GB_
GCA_903872565.1:0.42122,GB_GCA_016930955.1:0.40656)78.0:0.04267,(GB_GCA_017623265.1:0.5
1781,(GB_GCA_001803225.1:0.28063,(GB_GCA_016936495.1:0.29152,(GB_GCA_015061545.1:0.429
761,bin.144:0.226847):0.0948793)100.0:0.06425)51.0:0.02838)100.0:0.05699)72.0:0.04215)'100.0:o
__Victivallales':0.13293,(GB_GCA_002340265.1:0.49654,(GB_GCA_903921725.1:0.40674,(((GB_GCA
_018676675.1:0.25401,GB_GCA_001803235.1:0.27848)60.0:0.04454,(GB_GCA_019136035.1:0.3518
4,GB_GCA_002070945.1:0.4644)89.0:0.06414)82.0:0.04914,((GB_GCA_003552565.1:0.46881,GB_GC
A_002068325.1:0.32034)100.0:0.0661,GB_GCA_013151865.1:0.29635)92.0:0.0424)59.0:0.03202)'10
0.0:o__UBA1407':0.09273)44.0:0.04692)69.0:0.04843,(RS_GCF_000170755.1:0.56835,(GB_GCA_013
215875.1:0.42711,GB_GCA_003477185.1:0.45658)68.0:0.08476)85.0:0.07444)'100.0:c__Lentisphaeri
a':0.19116)35.0:0.04292)'100.0:p__Verrucomicrobiota':0.10952,((GB_GCA_017649705.1:0.44215,GB
_GCA_012514975.1:0.35478)'100.0:p__UBA3054;c__UBA3054;o__UBA3054':0.2538,(RS_GCF_00333
9615.1:0.81716,(GB_GCA_011064725.1:0.5425,((GB_GCA_010031885.1:0.67858,(GB_GCA_0168665
75.1:0.38021,(GB_GCA_902609415.1:0.54504,GB_GCA_016866545.1:0.40074)86.0:0.0532)82.0:0.05
581)'86.0:o__2-12-FULL-49-
11':0.06834,((((((GB_GCA_002401865.1:0.48899,GB_GCA_018062315.1:0.33797)52.0:0.05665,RS_G
CF_015356785.1:0.32867)86.0:0.04956,(GB_GCA_002402025.1:0.35058,GB_GCA_016213235.1:0.36
049)28.0:0.0548)38.0:0.04835,(GB_GCA_018240065.1:0.37522,GB_GCA_903867585.1:0.51162)93.0:
0.03919)28.0:0.02662,GB_GCA_011065175.1:0.62403)30.0:0.04011,((((GB_GCA_009885615.1:0.486
79,(GB_GCA_018239785.1:0.44797,(GB_GCA_017116025.1:0.35208,GB_GCA_018694295.1:0.34053)
51.0:0.04214)6.0:0.03312)0.0:0.03379,(((GB_GCA_007124415.1:0.39717,((GB_GCA_011065005.1:0.2
7921,GB_GCA_013821265.1:0.37101)36.0:0.02599,(GB_GCA_013287645.1:0.38825,RS_GCF_000750
955.1:0.36876)73.0:0.0354)81.0:0.02856)88.0:0.03074,GB_GCA_002709385.1:0.39256)7.0:0.01899,
GB_GCA_018239765.1:0.51025)1.0:0.02339)9.0:0.02371,(GB_GCA_018239815.1:0.42383,RS_GCF_0
02817655.1:0.73677)98.0:0.09434)7.0:0.02233,(GB_GCA_014239035.1:0.42086,GB_GCA_009937555
.1:0.43665)24.0:0.07223)47.0:0.04243)'32.0:o__Chlamydiales':0.05473)64.0:0.05154)86.0:0.06299)'1
00.0:p__Chlamydiota;c__Chlamydiia':0.2925)56.0:0.05817)63.0:0.04426)80.0:0.0615,((((GB_GCA_01

1333865.1:0.4322,(GB_GCA_011329245.1:0.47708,(GB_GCA_003644395.1:0.27723,GB_GCA_01692
8555.1:0.39741)62.0:0.05031)98.0:0.06523)'100.0:o__B48-
G9':0.18436,(GB_GCA_002791075.1:0.45696,GB_GCA_002069365.1:0.66857)86.0:0.08717)'100.0:p_
_Ratteibacteria;c__UBA8468':0.18392,((GB_GCA_016783345.1:0.54289,(GB_GCA_013151555.1:0.25
267,GB_GCA_003574845.1:0.38548)'100.0:p__NPL-UPA2;c__NPL-UPA2;o__NPL-
UPA2':0.14044)54.0:0.06544,(GB_GCA_018812485.1:0.49295,(GB_GCA_013152825.1:0.48423,GB_G
CA_018812925.1:0.53492)'100.0:p__CAIJMQ01;c__CAIJMQ01':0.15412)54.0:0.06531)51.0:0.04377)1
7.0:0.03704,(((GB_GCA_003645805.1:0.57546,(GB_GCA_016190205.1:0.5725,((GB_GCA_016928875.
1:0.39951,GB_GCA_018823795.1:0.37054)'100.0:o__JAHJGZ01':0.10728,(GB_GCA_016206925.1:0.4
934,(GB_GCA_016206875.1:0.44834,(((GB_GCA_016206995.1:0.35096,GB_GCA_016206815.1:0.380
85)100.0:0.06217,(GB_GCA_016179745.1:0.36218,GB_GCA_018883425.1:0.42674)100.0:0.0737)100
.0:0.05146,GB_GCA_016209715.1:0.36933)'100.0:o__Omnitrophales':0.054)69.0:0.03025)97.0:0.045
32)'100.0:c__Omnitrophia':0.0931)97.0:0.04972)80.0:0.02897,((((((GB_GCA_015233235.1:0.354012,
bin.281:0.216861):0.0586038,bin.565:0.271687):0.00115335,bin.644:0.260538):0.314131,(GB_GCA_
002085265.1:0.50211,GB_GCA_018399975.1:0.46058)64.0:0.08899)25.0:0.05681,((((((GB_GCA_0188
23915.1:0.353453,bin.658:0.335827):0.156417,GB_GCA_003644445.1:0.45096)'100.0:o__4484-
171':0.18426,GB_GCA_015494585.1:0.67518)46.0:0.06129,(((GB_GCA_001805615.1:0.30182,GB_GC
A_002753745.1:0.53281)'100.0:o__UBA10015':0.18308,GB_GCA_016867135.1:0.32944)82.0:0.0634
4,(((((GB_GCA_018813085.1:0.38198,GB_GCA_016214325.1:0.46126)97.0:0.05181,GB_GCA_002340
085.1:0.4145)18.0:0.02701,(GB_GCA_004299495.1:0.42634,GB_GCA_018400015.1:0.46885)42.0:0.0
4147)23.0:0.03781,GB_GCA_018830005.1:0.51728)'54.0:o__GIF10':0.0285,(GB_GCA_002796125.1:0
.42035,(GB_GCA_003451585.1:0.44723,(GB_GCA_013203265.1:5.2153e-06,bin.343:6.11352e-
06):0.341785)'99.0:o__UBA1572':0.05705)68.0:0.03938)47.0:0.03168)93.0:0.05171)79.0:0.06583,((G
B_GCA_018399295.1:0.28401,GB_GCA_003645745.1:0.37681)'100.0:o__B26-
G9':0.18528,GB_GCA_013626165.1:0.59237)90.0:0.07258)11.0:0.02894,(((GB_GCA_002783585.1:0.4
1818,(GB_GCA_001805885.1:0.42136,(GB_GCA_016928375.1:0.37082,((GB_GCA_013202935.1:9.97
589e-06,bin.468:6.11352e-
06):0.188361,bin.126:0.19746):0.138519)'100.0:o__JABMRG01':0.11265)39.0:0.03938)55.0:0.03886,
(((GB_GCA_018823705.1:0.49624,((GB_GCA_013202515.1:5.01572e-06,bin.93:6.11352e-
06):0.328705,(((GB_GCA_001804045.1:0.23167,bin.346:0.168236):0.00953433,bin.423:0.212881):0.
017961,bin.409:0.167963):0.171535)'100.0:o__JABMRN01':0.09767)56.0:0.04827,(GB_GCA_002780
005.1:0.34605,(GB_GCA_013203355.1:5.62317e-06,bin.702:6.11352e-
06):0.368514)37.0:0.05187)14.0:0.02936,(((GB_GCA_013202205.1:5.68909e-06,bin.291:6.11352e-
06):0.372834,(GB_GCA_016783555.1:0.188938,bin.182:0.18184):0.181872)'81.0:o__JABMRJ01':0.05
218,GB_GCA_016931215.1:0.49126)43.0:0.04593)60.0:0.0531)9.0:0.03084,(GB_GCA_016932635.1:0
.495,((GB_GCA_011334725.1:0.39805,GB_GCA_016867055.1:0.40409)21.0:0.06031,(((GB_GCA_0189
02915.1:0.153325,bin.477:0.132138):0.00712343,bin.352:0.140542):0.297131,GB_GCA_016206785.
1:0.4587)21.0:0.04742)0.0:0.02804)2.0:0.03016)3.0:0.02959)13.0:0.03411)2.0:0.02471,((GB_GCA_01
6930485.1:0.43145,GB_GCA_016177145.1:0.46088)61.0:0.08139,((GB_GCA_016196505.1:0.45288,G
B_GCA_015231685.1:0.5178)'100.0:o__2-02-FULL-51-
18':0.15519,(((GB_GCA_016206755.1:0.36628,GB_GCA_016196465.1:0.39571)'100.0:o__1-14-0-20-
64-
10':0.14362,(GB_GCA_016206665.1:0.39586,GB_GCA_016206565.1:0.55173)'100.0:o__UBA9649':0.
09806)96.0:0.06117,GB_GCA_016929445.1:0.59586)20.0:0.04499)38.0:0.04903)14.0:0.04332)'93.0:c
__Koll11':0.05236)'96.0:p__Omnitrophota':0.05075,GB_GCA_001730085.1:0.74831)27.0:0.03463)25.
0:0.0389)5.0:0.03854,(((((GB_GCA_011334435.1:0.20326,GB_GCA_014730235.1:0.24254)'100.0:c__
DTPJ01;o__DTPJ01':0.17299,(GB_GCA_003645735.1:0.32273,(GB_GCA_016866785.1:0.49305,(GB_G
CA_002709695.1:0.38855,(GB_GCA_003635265.1:0.22584,GB_GCA_012267415.1:0.40811)97.0:0.04

497)'100.0:o__WGA-4E':0.16761)87.0:0.04587)'100.0:c__WGA-
4E':0.08113)'100.0:p__Poribacteria':0.12952,((GB_GCA_003598085.1:0.175103,bin.86:0.200965):0.2
99187,(GB_GCA_011338105.1:0.40418,(GB_GCA_002746185.1:0.377,((GB_GCA_018005585.1:0.247
98,((GB_GCA_007116095.1:0.2408,GB_GCA_002070275.1:0.47117)23.0:0.02025,((GB_GCA_0161826
45.1:0.21673,GB_GCA_016125095.1:0.30005)41.0:0.03472,GB_GCA_903837785.1:0.21656)17.0:0.01
952)87.0:0.03636)88.0:0.03837,GB_GCA_016934125.1:0.29523)80.0:0.03783)100.0:0.08476)'100.0:p
__Hydrogenedentota;c__Hydrogenedentia;o__Hydrogenedentiales':0.25955)70.0:0.09631)16.0:0.03
148,((GB_GCA_003598175.1:0.57137,(GB_GCA_015075175.1:0.52004,GB_GCA_013360775.1:0.5501
2)95.0:0.08156)'100.0:p__OLB16;c__OLB16':0.14366,(((GB_GCA_012513815.1:0.43312,GB_GCA_017
161655.1:0.35646)'100.0:o__Sumerlaeales':0.18466,((GB_GCA_007132505.1:0.6108,GB_GCA_01692
8695.1:0.54201)7.0:0.05152,((GB_GCA_002070185.1:0.49117,((GB_GCA_003501375.1:0.309593,bin.
579:0.196393):0.327237,(GB_GCA_017161705.1:0.37213,GB_GCA_016928995.1:0.48308)'98.0:o__J
ADFCW01':0.08183)21.0:0.03574)18.0:0.03319,(GB_GCA_017161665.1:0.48286,GB_GCA_002068205
.1:0.45346)33.0:0.09281)23.0:0.03275)35.0:0.03808)'100.0:p__Sumerlaeota;c__Sumerlaeia':0.14409
,GB_GCA_002428325.1:0.58598)25.0:0.04834)34.0:0.05363)19.0:0.02482,((((GB_GCA_014728225.1:
0.7446,(GB_GCA_003647715.1:0.60841,GB_GCA_011773985.1:0.54243)'100.0:c__B26-
G2':0.11718)'100.0:p__RBG-13-66-
14':0.1137,((((((GB_GCA_005239795.1:0.49606,GB_GCA_016206515.1:0.57679)'100.0:c__SBBH01;o_
_SBBH01':0.25815,((((GB_GCA_002711105.1:0.76976,(GB_GCA_002402755.1:0.63274,(GB_GCA_016
209605.1:0.37456,(GB_GCA_016209535.1:0.34147,GB_GCA_002320775.1:0.4279)100.0:0.07526)'10
0.0:o__UBA8108':0.13094)86.0:0.06357)'100.0:c__UBA8108':0.09112,(GB_GCA_003694875.1:0.5358
7,(GB_GCA_016200955.1:0.35831,GB_GCA_016765095.1:0.51791)'100.0:o__J058':0.23911)'84.0:c__
J058':0.08999)35.0:0.03501,GB_GCA_016209495.1:0.54452)46.0:0.0272,((((GB_GCA_011525755.1:0.
5049,GB_GCA_016795345.1:0.54108)'100.0:c__PLA2;o__PLA2':0.14006,(GB_GCA_016178505.1:0.48
596,(GB_GCA_016935485.1:0.48474,GB_GCA_016209595.1:0.55001)49.0:0.06855)56.0:0.06659)27.
0:0.047,((GB_GCA_015485455.1:0.5207,(GB_GCA_017853435.1:0.45982,GB_GCA_013213915.1:0.42
555)100.0:0.11673)'100.0:c__GCA-002687715;o__GCA-
002687715':0.16932,((GB_GCA_016795255.1:0.5237,(GB_GCA_003644265.1:0.56617,GB_GCA_0168
72815.1:0.55845)78.0:0.08083)'100.0:c__B15-G4;o__B15-
G4':0.09782,((GB_GCA_011321845.1:0.441,GB_GCA_016716265.1:0.48294)'100.0:o__UBA2386':0.1
4754,((GB_GCA_016793245.1:0.49248,GB_GCA_016200225.1:0.56863)99.0:0.07462,(GB_GCA_0168
72715.1:0.71473,(((GB_GCA_004295085.1:0.302865,bin.406:0.316196):0.0141082,bin.362:0.30561):
0.00427936,bin.524:0.338423):0.354577)98.0:0.05037)'85.0:o__UBA1135':0.04273)'100.0:c__UBA11
35':0.05299)100.0:0.05505)95.0:0.0499)39.0:0.03261,((GB_GCA_013360495.1:0.44618,GB_GCA_019
186845.1:0.43659)'100.0:o__UBA2392':0.29278,(GB_GCA_007127275.1:0.57663,GB_GCA_01620958
5.1:0.57317)54.0:0.07066)'92.0:c__UBA8742':0.0998)8.0:0.02504)45.0:0.02779)21.0:0.02899,(((((GB
_GCA_014584395.1:0.39323,((RS_GCF_013694095.1:0.524023,bin.382:0.394669):0.029296,bin.140:
0.433245):0.101451)'100.0:o__Gemmatales':0.14064,GB_GCA_007748055.1:0.49204)31.0:0.04143,((
(RS_GCF_007747995.1:0.2551,GB_GCA_016235885.1:0.29132)59.0:0.03706,(GB_GCA_017795105.1:
0.420504,bin.212:0.314077):0.107456)'100.0:o__Planctomycetales':0.15313,((((GB_GCA_017447865
.1:0.66228,(GB_GCA_016177915.1:0.2608,GB_GCA_015488215.1:0.29546)65.0:0.0773)35.0:0.02274,
(((GB_GCA_003389325.1:0.2162,GB_GCA_003576845.1:0.2607)23.0:0.02258,(GB_GCA_016793185.1
:0.23146,(GB_GCA_016191115.1:0.18881,GB_GCA_018242585.1:0.26954)83.0:0.01945)93.0:0.03409
)30.0:0.02405,(((RS_GCF_001642955.1:0.472705,bin.112:0.328233):0.259965,((GB_GCA_014237955.
1:0.33464,RS_GCF_007859935.1:0.39672)48.0:0.011876,bin.20:0.257309):0.010774)72.0:0.0111758,
bin.375:0.269364):0.0142842)33.0:0.01764)39.0:0.03711,((((((GB_GCA_903949225.1:0.17332,bin.5
66:0.12718):0.00659469,bin.657:0.129444):0.000839685,bin.655:0.118784):0.00890742,bin.333:0.1
36469):0.00580891,bin.390:0.123002):0.00637728,bin.76:0.12177):0.0206992,bin.29:0.166647):0.33

5723)85.0:0.04056,GB_GCA_009664345.1:0.36132)'100.0:o__Pirellulales':0.11037)100.0:0.07859)25.
0:0.03347,RS_GCF_011064595.1:0.59728)'100.0:c__Planctomycetia':0.1599,((GB_GCA_003170085.1:
0.45247,GB_GCA_012729015.1:0.41734)'100.0:o__FEN-
1346':0.14049,((((GB_GCA_002685935.1:0.3386,GB_GCA_002686995.1:0.40929)100.0:0.06503,((GB
_GCA_016873335.1:0.75705,GB_GCA_009827205.1:0.59541)56.0:0.03212,GB_GCA_900696685.1:0.
3877)100.0:0.08219)'100.0:o__Phycisphaerales':0.15992,(GB_GCA_009781185.1:0.56052,GB_GCA_0
11358525.1:0.53224)86.0:0.0561)100.0:0.0741,((GB_GCA_011682385.1:0.45581,(GB_GCA_0169345
15.1:0.45012,(RS_GCF_002117005.1:0.43132,((GB_GCA_016873215.1:0.350877,bin.237:0.224):0.07
98528,((GB_GCA_001824635.1:0.28093,GB_GCA_003142595.1:0.37301)39.0:0.03534,(((GB_GCA_01
2744475.1:0.232199,bin.397:0.175198):0.0179298,bin.640:0.228799):0.0133294,bin.83:0.224489):0.
125122)50.0:0.02915)66.0:0.04076)100.0:0.14823)'100.0:o__Sedimentisphaerales':0.09254)98.0:0.0
5392,(((GB_GCA_003171335.1:0.232525,bin.536:0.226534):0.154655,(GB_GCA_016782885.1:0.2801
59,bin.461:0.216716):0.192941)'100.0:o__SM23-
33':0.15504,(GB_GCA_012729815.1:0.46447,(GB_GCA_900696525.1:0.52457,(GB_GCA_003576905.
1:0.51655,((GB_GCA_016860685.1:0.54628,GB_GCA_012729395.1:0.42563)43.0:0.02548,GB_GCA_0
11053305.1:0.35671)95.0:0.03436)99.0:0.04378)'100.0:o__UBA1845':0.0689)98.0:0.0412)35.0:0.029
12)46.0:0.03584)100.0:0.0773)'100.0:c__Phycisphaerae':0.06736)100.0:0.09139)13.0:0.0324,(((((GB_
GCA_001302825.1:0.40818,GB_GCA_007131925.1:0.50693)'91.0:c__DG-
23':0.10584,((GB_GCA_013152475.1:0.3899,GB_GCA_903901045.1:0.49362)96.0:0.08217,((GB_GCA
_001828545.1:0.40881,(GB_GCA_016198835.1:0.25691,(GB_GCA_018648405.1:0.47156,GB_GCA_0
00987375.1:0.41355)94.0:0.04513)100.0:0.05587)'100.0:o__Brocadiales':0.13312,GB_GCA_0127288
35.1:0.70862)50.0:0.03331)'80.0:c__Brocadiae':0.03773)55.0:0.03868,((((GB_GCA_016220095.1:0.48
342,(GB_GCA_016207825.1:0.35807,GB_GCA_016235865.1:0.40726)100.0:0.12994)'99.0:o__JACQXL
01':0.08031,(GB_GCA_009773755.1:0.54153,(GB_GCA_016192525.1:0.41606,GB_GCA_016197205.1
:0.39363)45.0:0.04666)'95.0:o__WSZJ01':0.05117)82.0:0.03423,((GB_GCA_001828445.1:0.39002,(GB
_GCA_016206485.1:0.97238,GB_GCA_016200125.1:0.4132)56.0:0.03609)'97.0:o__MHYJ01':0.07274,
GB_GCA_011327745.1:0.44259)97.0:0.07495)'100.0:c__MHYJ01':0.13402,((((GB_GCA_016872875.1:
0.24879,bin.340:0.248666):0.0109685,bin.272:0.303733):0.277812,((GB_GCA_013202185.1:6.57593
e-06,bin.53:6.11352e-
06):0.430953,(GB_GCA_003553185.1:0.235337,bin.318:0.246627):0.245183)86.0:0.05508)62.0:0.05
378,(GB_GCA_016179685.1:0.247692,bin.8:0.291611):0.279598)'99.0:c__PUPC01':0.06535)6.0:0.023
51)15.0:0.02397,GB_GCA_003644325.1:0.57638)26.0:0.02065,((GB_GCA_003694635.1:0.61676,(GB_
GCA_009002475.1:0.37487,GB_GCA_017993505.1:0.46343)'100.0:o__SRT547':0.20205)'97.0:c__J13
2':0.12569,(GB_GCA_011371585.1:0.66593,GB_GCA_004376375.1:0.51043)70.0:0.08917)27.0:0.052
35)26.0:0.03557)62.0:0.03991,(((GB_GCA_016927125.1:0.35767,GB_GCA_016936955.1:0.41143)'10
0.0:o__SZUA-
567':0.22321,(GB_GCA_016930595.1:0.65623,GB_GCA_016200135.1:0.57053)100.0:0.09059)'99.0:c
__SZUA-
567':0.06892,(GB_GCA_013816415.1:0.84533,(GB_GCA_002746535.1:0.67564,GB_GCA_015231345.
1:0.75971)78.0:0.09169)'99.0:c__UBA11346':0.10683)69.0:0.0566)18.0:0.03814,GB_GCA_00364427
5.1:0.63851)'100.0:p__Planctomycetota':0.12147)4.0:0.04694,(((((((GB_GCA_903862805.1:0.37556,(
GB_GCA_014728015.1:0.33328,(GB_GCA_014729375.1:0.35286,GB_GCA_903888815.1:0.39541)91.
0:0.036)100.0:0.06695)61.0:0.05738,(RS_GCF_000474745.1:0.39058,GB_GCA_009779925.1:0.46337
)100.0:0.15163)'100.0:c__Chitinivibrionia;o__Chitinivibrionales':0.21113,((GB_GCA_001800515.1:0.4
162,(GB_GCA_016788315.1:0.35933,GB_GCA_001789205.1:0.29722)70.0:0.05679)'100.0:c__OXYB2-
FULL-49-7;o__OXYB2-FULL-49-
7':0.2134,(GB_GCA_016715065.1:0.52774,((GB_GCA_902796785.1:0.45213,GB_GCA_001462235.1:0
.6073)'100.0:o__Fibrobacterales':0.11457,(GB_GCA_015232855.1:0.3886,GB_GCA_900299345.1:0.3

559)'100.0:o__UBA11236':0.13525)96.0:0.06139)'100.0:c__Fibrobacteria':0.1611)68.0:0.05182)'100.0:p__Fibrobacterota':0.12273,((GB_GCA_016927185.1:0.75666,GB_GCA_017997825.1:0.62761)24.0:0.0779,((GB_GCA_003564155.1:0.41847,((GB_GCA_002742885.1:0.3289,((GB_GCA_014382915.1:0.175013,bin.287:0.169707):0.0136586,bin.262:0.145248):0.154468)99.0:0.04222,(GB_GCA_009782695.1:0.49171,(GB_GCA_002424605.1:0.37241,GB_GCA_016838885.1:0.35204)100.0:0.05837)97.0:0.03775)96.0:0.04743)'100.0:o__Cloacimonadales':0.13212,(GB_GCA_003647765.1:0.34157,(GB_GCA_001577135.1:0.37587,(GB_GCA_016932035.1:0.090574,bin.679:0.0977806):0.227066)100.0:0.12331)'100.0:o__JGIOTU-
2':0.10003)'100.0:p__Cloacimonadota;c__Cloacimonadia':0.20311)35.0:0.06099)26.0:0.03548,(((((GB_GCA_018263585.1:0.56621,GB_GCA_016703065.1:0.69416)74.0:0.05776,(((GB_GCA_002127415.1:0.222895,bin.11:0.206164):0.0351975,bin.407:0.243987):0.299948,GB_GCA_018812585.1:0.5317)61.0:0.05312)32.0:0.02982,(GB_GCA_016783645.1:0.28582,(GB_GCA_013202315.1:7.77863e-
06,bin.685:6.11352e-06):0.254882)'100.0:c__JABMRZ01;o__JABMRZ01':0.23165)'90.0:p__AABM5-
125-
24':0.06236,((GB_GCA_903910645.1:0.48349,GB_GCA_016784425.1:0.4328)'100.0:o__CAIWAD01':0.15637,GB_GCA_016938275.1:0.71348)'82.0:p__Delongbacteria;c__UBA4055':0.08422)75.0:0.05582,((((GB_GCA_002049825.1:0.26251,GB_GCA_003695285.1:0.2683)'100.0:o__RBG-13-44-
9':0.15123,(GB_GCA_003696775.1:0.31211,GB_GCA_016928315.1:0.32678)'100.0:o__Calditrichales':0.16645)'100.0:p__Calditrichota;c__Calditrichia':0.10522,((GB_GCA_016783505.1:0.39146,(GB_GCA_003644825.1:0.34072,GB_GCA_001304035.1:0.4101)'100.0:p__SM23-31;c__SM23-
31':0.08463)71.0:0.06849,(((GB_GCA_004356825.1:0.36671,(GB_GCA_011047125.1:0.34136,GB_GCA_008501765.1:0.46265)'100.0:o__DRLW01':0.05537)74.0:0.04164,(((GB_GCA_014728085.1:0.30399,(GB_GCA_002327255.1:0.32043,GB_GCA_011682565.1:0.28294)79.0:0.03027)'100.0:o__UBA2214':0.13973,(GB_GCA_014359355.1:0.30969,GB_GCA_011389025.1:0.3368)'97.0:o__JACIZP01':0.0521)100.0:0.04371,(GB_GCA_016927835.1:0.35333,(GB_GCA_016783825.1:0.26953,GB_GCA_016931935.1:0.31715)100.0:0.06725)'100.0:o__AABM5-25-
91':0.07828)43.0:0.02153)'100.0:p__KSB1;c__UBA2214':0.03984,(((GB_GCA_011040895.1:0.26104,GB_GCA_002011685.1:0.18677)100.0:0.09229,(GB_GCA_002085035.1:0.26251,GB_GCA_013151735.1:0.23831)64.0:0.0421)'100.0:p__JdFR-76;c__JdFR-76;o__JdFR-
76':0.11405,(GB_GCA_011363065.1:0.30892,((GB_GCA_016931965.1:0.27294,GB_GCA_003645915.1:0.39413)38.0:0.02937,(GB_GCA_013202295.1:5.5426e-06,bin.322:6.11352e-
06):0.363234)57.0:0.03778)'100.0:p__QNDG01;c__QNDG01;o__QNDG01':0.16273)48.0:0.02835)87.0:0.04054)74.0:0.0309)48.0:0.0272,((GB_GCA_004402895.1:0.54107,((((GB_GCA_016784265.1:0.58112,(((GB_GCA_014381485.1:0.30135,(GB_GCA_002471865.1:0.28593,GB_GCA_002329125.1:0.37013)54.0:0.03335)100.0:0.1281,(GB_GCA_902576865.1:0.71135,(((GB_GCA_001577055.1:0.175658,bin.522:0.154694):0.000420454,bin.120:0.154723):0.151641,((GB_GCA_002713885.1:0.49763,GB_GCA_902615915.1:0.51944)37.0:0.02887,((GB_GCA_013203145.1:8.69415e-06,bin.159:6.11352e-
06):0.284881,((GB_GCA_013152835.1:0.31649,GB_GCA_018812885.1:0.27398)26.0:0.02184,GB_GCA_002346675.1:0.38673)17.0:0.02264)30.0:0.03289)26.0:0.03139)100.0:0.05764)100.0:0.09701)'100.0:o__Marinisomatales':0.09002,(GB_GCA_004124295.1:0.47,(((((GB_GCA_002731795.1:0.39137,(GB_GCA_002711465.1:0.37711,GB_GCA_002719095.1:0.45201)62.0:0.05832)28.0:0.03003,GB_GCA_002705605.1:0.4717)33.0:0.04392,(GB_GCA_002170735.1:0.39615,GB_GCA_000405805.1:0.32814)15.0:0.03563)61.0:0.06498,GB_GCA_004124405.1:0.30722)64.0:0.0576,((GB_GCA_002703375.1:0.37823,GB_GCA_014381925.1:0.35584)34.0:0.04698,(GB_GCA_014381725.1:0.219464,bin.70:0.293281):0.053096)18.0:0.02665)100.0:0.15388)'100.0:o__SCGC-AAA003-
L08':0.07487)'100.0:c__Marinisomatia':0.05585)33.0:0.03331,((GB_GCA_011049095.1:0.35802,GB_GCA_002452555.1:0.38745)'100.0:c__AB16;o__AB16':0.18699,(((((GB_GCA_013202385.1:9.43283e-
06,bin.509:6.11352e-

06):0.0601759,bin.360:0.0279593):0.0410757,bin.62:0.0583045):0.0103413,bin.81:0.0558328):0.025
9254,bin.398:0.0848984):0.480662)31.0:0.0497)40.0:0.0245,(GB_GCA_017656425.1:0.36067,(GB_G
CA_016934035.1:0.39677,GB_GCA_011046365.1:0.40533)99.0:0.06626)'100.0:c__UBA2242;o__UBA
2242':0.16434)64.0:0.03112,GB_GCA_018263365.1:0.42179)94.0:0.0463)'100.0:p__Marinisomatota'
:0.07935,((GB_GCA_008501735.1:0.52434,GB_GCA_013359335.1:0.50204)'76.0:p__CLD3;c__CLD3':0
.06785,(((GB_GCA_017303995.1:0.52212,((RS_GCF_002240205.1:0.207336,bin.97:0.202287):0.2028
04,RS_GCF_000020525.1:0.2741)'100.0:c__Chlorobia;o__Chlorobiales':0.2268)83.0:0.04489,((((GB_G
CA_006226965.1:0.28735,(GB_GCA_007131535.1:0.32318,(GB_GCA_001314545.1:0.24614,GB_GCA
_007125355.1:0.30875)90.0:0.03221)91.0:0.02561)96.0:0.03427,GB_GCA_002720745.1:0.59067)'10
0.0:o__Balneolales':0.18923,(GB_GCA_017303715.1:0.32378,((GB_GCA_014879465.1:0.34704,(((GB
_GCA_003285105.1:0.13827,GB_GCA_011682235.1:0.13583)59.0:0.03588,((RS_GCF_002894645.1:0.
4816,GB_GCA_009839485.1:0.31112)23.0:0.02309,(GB_GCA_002238805.1:0.48611,RS_GCF_017908
595.1:0.23958)23.0:0.03388)28.0:0.01724)92.0:0.03057,(GB_GCA_013003255.1:0.229412,bin.449:0.
236013):0.0907983)98.0:0.03218)78.0:0.03345,GB_GCA_013003465.1:0.27233)100.0:0.05133)'100.0
:o__Rhodothermales':0.1436)'100.0:c__Rhodothermia':0.07226,(((GB_GCA_009924095.1:0.34824,(G
B_GCA_016718825.1:0.31635,GB_GCA_013298365.1:0.40541)95.0:0.05976)'100.0:o__J057':0.09624
,((GB_GCA_001769655.1:0.46363,(GB_GCA_002325615.1:0.46829,((((GB_GCA_902628365.1:0.4191
81,bin.302:0.2358):0.0544289,RS_GCF_014323545.1:0.29295)59.0:0.04493,GB_GCA_011524675.1:0.
31798)28.0:0.03734,((((GB_GCA_004293915.1:0.39238,RS_GCF_000775915.1:0.25378)89.0:0.03435,
(RS_GCF_017571495.1:0.42762,((GB_GCA_004293265.1:0.45894,RS_GCF_002843425.1:0.34133)100
.0:0.04003,((GB_GCA_902805615.1:0.26287,(RS_GCF_000218895.1:0.257082,bin.530:0.254138):0.0
854377)49.0:0.02214,RS_GCF_900112255.1:0.26156)50.0:0.02114)35.0:0.01847)71.0:0.01788)82.0:0
.02223,(GB_GCA_002729595.1:0.3999,RS_GCF_000422585.1:0.42145)100.0:0.04536)46.0:0.01591,R
S_GCF_900167975.1:0.47156)31.0:0.02785)28.0:0.025)'100.0:o__Cytophagales':0.0564)99.0:0.03684
,(((GB_GCA_013288555.1:0.34527,(GB_GCA_016776505.1:0.41422,GB_GCA_016124515.1:0.36908)5
2.0:0.03633)'100.0:o__CAILMK01':0.09418,((((GB_GCA_015487695.1:0.31984,(GB_GCA_003022985.
1:0.35127,GB_GCA_007695095.1:0.31961)69.0:0.04342)82.0:0.05114,(GB_GCA_018401955.1:0.3203
2,GB_GCA_016706865.1:0.40603)30.0:0.02836)36.0:0.02775,((((GB_GCA_013001465.1:0.31468,GB_
GCA_016777395.1:0.37284)61.0:0.04625,(GB_GCA_903861145.1:0.42471,GB_GCA_016787225.1:0.4
9376)42.0:0.03582)65.0:0.03937,GB_GCA_017984135.1:0.48701)34.0:0.02477,((GB_GCA_01437966
5.1:0.40423,bin.630:0.243659):0.2383,GB_GCA_016711295.1:0.42701)48.0:0.02991)7.0:0.0161)19.0:
0.02173,(GB_GCA_013695715.1:0.39763,(GB_GCA_903871715.1:0.34141,((RS_GCF_018255755.1:0.
232662,bin.227:0.214123):0.0167933,bin.279:0.249473):0.184075)86.0:0.04054)100.0:0.04601)'100.
0:o__Chitinophagales':0.06112)21.0:0.01971,(((((((GB_GCA_002737705.1:0.159093,bin.96:0.082274)
:0.285103,bin.544:0.258729):0.0911536,(((GB_GCA_002428905.1:0.189636,bin.396:0.141416):0.139
342,bin.517:0.301295):0.010087,bin.317:0.220793):0.103145)100.0:0.0523,GB_GCA_008933805.1:0.
2939)77.0:0.0288,((GB_GCA_009693575.1:0.3082,GB_GCA_903897855.1:0.38093)96.0:0.05164,GB_
GCA_013298895.1:0.37437)75.0:0.03022)'99.0:o__NS11-
12g':0.05215,GB_GCA_005799025.1:0.65113)53.0:0.03029,((GB_GCA_016763665.1:0.33104,(GB_GC
A_903958995.1:0.2059,(RS_GCF_008033235.1:0.244435,bin.659:0.191767):0.0320253)'100.0:o__Sp
hingobacteriales':0.07882)72.0:0.02924,(((GB_GCA_016788165.1:0.35216,GB_GCA_016720655.1:0.3
2703)63.0:0.02943,GB_GCA_903911135.1:0.26927)'100.0:o__AKYH767-
A':0.04906,(((GB_GCA_016193485.1:0.42572,GB_GCA_903863935.1:0.35425)8.0:0.05993,((((((GB_G
CA_013286385.1:0.26227,GB_GCA_017303975.1:0.22045)49.0:0.03141,(GB_GCA_013823565.1:0.39
442,GB_GCA_003151815.1:0.23366)28.0:0.02092)61.0:0.0207,GB_GCA_903873905.1:0.32089)95.0:0
.02059,GB_GCA_016213405.1:0.32877)7.0:0.02637,(GB_GCA_903842515.1:0.34373,GB_GCA_01437
6525.1:0.35682)51.0:0.02358)4.0:0.02467,(GB_GCA_013696965.1:0.20341,GB_GCA_005773695.1:0.
24578)49.0:0.04413)6.0:0.02068)'25.0:o__AKYH767':0.02658,(((GB_GCA_018812245.1:0.33857,((GB

_GCA_007122745.1:0.49109,(GB_GCA_001768555.1:0.35683,(GB_GCA_017998135.1:0.34041,GB_G
CA_003565735.1:0.26214)23.0:0.03939)14.0:0.03654)17.0:0.02474,((((((GB_GCA_016744655.1:0.27
905,GB_GCA_015489405.1:0.34544)2.0:0.02783,(GB_GCA_905233445.1:0.422618,bin.586:0.211263)
:0.0629324)1.0:0.02424,(((((GB_GCA_002389705.1:0.29151,GB_GCA_002426025.1:0.26199)66.0:0.0
2941,(GB_GCA_013390605.1:0.23543,GB_GCA_014860575.1:0.2447)39.0:0.03013)79.0:0.02615,(GB
_GCA_903923925.1:0.26034,GB_GCA_018001695.1:0.28619)17.0:0.02931)13.0:0.01819,((GB_GCA_9
03903985.1:0.188185,bin.48:0.166038):0.164475,((GB_GCA_903847905.1:0.24319,GB_GCA_903862
465.1:0.29013)35.0:0.02496,(GB_GCA_903899825.1:0.23434,GB_GCA_903922465.1:0.26715)71.0:0.
03964)79.0:0.02714)38.0:0.0238)10.0:0.02103,(((GB_GCA_017991835.1:0.24285,(GB_GCA_0184002
95.1:0.231957,bin.184:0.182531):0.0452526)54.0:0.02079,GB_GCA_002840985.1:0.25061)57.0:0.02
063,((GB_GCA_002254335.1:0.149701,bin.444:0.149734):0.132219,GB_GCA_013151015.1:0.23746)
16.0:0.02131)37.0:0.01891)19.0:0.0247)0.0:0.01924,(GB_GCA_003648395.1:0.38048,GB_GCA_0028
69405.1:0.2969)18.0:0.03227)4.0:0.01537,(GB_GCA_007119395.1:0.35539,(GB_GCA_003250395.1:0
.2947,GB_GCA_009777825.1:0.31649)64.0:0.03091)4.0:0.02627)17.0:0.01377,((GB_GCA_012518765
.1:0.47876,GB_GCA_017524785.1:0.60735)76.0:0.02539,GB_GCA_016937255.1:0.42613)43.0:0.0276
7)49.0:0.02385)26.0:0.02419)54.0:0.02757,(((GB_GCA_002084355.1:0.31303,GB_GCA_002428105.1:
0.31454)21.0:0.0467,((GB_GCA_013153865.1:0.29941,GB_GCA_016937695.1:0.25034)41.0:0.03267,
(GB_GCA_016936595.1:0.41281,GB_GCA_017651345.1:0.39225)93.0:0.05501)16.0:0.02397)22.0:0.0
3375,(((GB_GCA_009786075.1:0.44123,(GB_GCA_002454955.1:0.26021,GB_GCA_016936665.1:0.29
535)43.0:0.03804)24.0:0.02519,((GB_GCA_017551905.1:0.33321,GB_GCA_002869335.1:0.31905)98.
0:0.04575,((((GB_GCA_002408065.1:0.30653,(GB_GCA_017964045.1:0.64872,((((((GB_GCA_9005475
55.1:0.14668,GB_GCA_015061035.1:0.24845)34.0:0.01826,(GB_GCA_900545915.1:0.12179,GB_GCA
_017467745.1:0.14045)41.0:0.02301)24.0:0.01538,(GB_GCA_017481745.1:0.23492,GB_GCA_001701
065.1:0.39698)33.0:0.03001)72.0:0.06259,(GB_GCA_017618715.1:0.42425,GB_GCA_902800365.1:0.
55745)76.0:0.03344)39.0:0.01957,((GB_GCA_002471225.1:0.18658,GB_GCA_009787405.1:0.18878)
39.0:0.02732,((GB_GCA_000010645.1:0.43597,GB_GCA_015478855.1:0.3512)47.0:0.02079,(GB_GCA
_900541275.1:0.68484,GB_GCA_001657575.1:0.2904)25.0:0.02905)25.0:0.02689)6.0:0.01444)64.0:0
.02076,GB_GCA_017446025.1:0.34685)56.0:0.02492)100.0:0.08469)99.0:0.04093,(GB_GCA_900766
825.1:0.40565,RS_GCF_018271995.1:0.31007)100.0:0.04408)99.0:0.0384,RS_GCF_001310955.1:0.28
443)62.0:0.01932,((GB_GCA_013203525.1:5.88425e-06,bin.676:6.11352e-
06):0.197714,bin.700:0.135879):0.187911)81.0:0.01859)51.0:0.01975)24.0:0.01872,(((GB_GCA_0184
01015.1:0.30636,GB_GCA_001769295.1:0.33161)61.0:0.0351,((((GB_GCA_002383425.1:0.31166,GB_
GCA_002748015.1:0.55213)89.0:0.03701,(RS_GCF_900096565.1:0.25405,((RS_GCF_000798815.1:0.2
1569,GB_GCA_902363575.1:0.59195)53.0:0.02988,GB_GCA_002439805.1:0.65171)98.0:0.03524)80.
0:0.03145)99.0:0.0408,((GB_GCA_012031335.1:0.23537,GB_GCA_012517825.1:0.25464)28.0:0.0355
7,(GB_GCA_002380425.1:0.32277,GB_GCA_011368345.1:0.29868)54.0:0.03123)52.0:0.03447)42.0:0
.03577,((GB_GCA_016935595.1:0.30416,((GB_GCA_017882345.1:0.390622,bin.410:0.208259):0.082
508,(GB_GCA_003648635.1:0.32691,GB_GCA_016783865.1:0.27084)88.0:0.02897)54.0:0.02344)68.
0:0.02089,(GB_GCA_018334675.1:0.3518,GB_GCA_007135095.1:0.36355)79.0:0.02483)71.0:0.01786
)20.0:0.01873)8.0:0.0153,(((GB_GCA_001769535.1:0.25227,GB_GCA_001768975.1:0.301)22.0:0.030
04,(GB_GCA_003648455.1:0.24853,(GB_GCA_018692315.1:0.24414,GB_GCA_016209245.1:0.28735)
25.0:0.03398)13.0:0.02476)3.0:0.02247,((GB_GCA_016212695.1:0.25185,(GB_GCA_011327585.1:0.3
6005,GB_GCA_002771395.1:0.35277)40.0:0.03023)5.0:0.02868,GB_GCA_001603595.1:0.43373)3.0:0
.02142)2.0:0.01936)1.0:0.01288)1.0:0.01812)100.0:0.06897)'55.0:o__Bacteroidales':0.03357,((GB_G
CA_016124845.1:0.29554,(GB_GCA_018401705.1:0.28313,GB_GCA_014879445.1:0.40224)65.0:0.03
628)27.0:0.0317,((((GB_GCA_013214975.1:0.32332,GB_GCA_018669945.1:0.29048)66.0:0.02499,GB
_GCA_016764395.1:0.37924)66.0:0.0342,GB_GCA_002790175.1:0.37339)39.0:0.02152,((((((GB_GCA
_002430755.1:0.341248,bin.585:0.305427):0.00222513,bin.316:0.36168):0.142727,GB_GCA_017798

125.1:0.26804)95.0:0.03733,((GB_GCA_016744755.1:0.28006,((GB_GCA_015489265.1:0.32621,RS_G
CF_002369955.1:0.40298)27.0:0.03558,((((RS_GCF_018861125.1:0.166362,bin.568:0.131312):0.0712
601,bin.193:0.183057):0.00865458,bin.694:0.207161):0.0401226,bin.539:0.172326):0.11968)28.0:0.
02465)6.0:0.01635,((GB_GCA_001880285.1:0.21717,GB_GCA_016025055.1:0.52926)72.0:0.02055,R
S_GCF_014252275.1:0.97277)89.0:0.06076)92.0:0.04854)92.0:0.03133,((GB_GCA_002685115.1:0.33
876,(GB_GCA_002706465.1:0.43054,GB_GCA_902533035.1:0.44624)91.0:0.07671)79.0:0.03956,(GB
_GCA_018608925.1:0.27808,GB_GCA_905181585.1:0.38618)93.0:0.06842)72.0:0.03845)96.0:0.0454
9,((GB_GCA_002720635.1:0.39588,((GB_GCA_009925805.1:0.70337,GB_GCA_002716065.1:0.36685)
98.0:0.03705,((GB_GCA_002336675.1:0.40014,(GB_GCA_013001705.1:0.33088,(RS_GCF_007995015
.1:0.32691,GB_GCA_007125175.1:0.3716)38.0:0.02247)34.0:0.02072)53.0:0.02372,(GB_GCA_01671
3875.1:0.48433,GB_GCA_002351955.1:0.26419)56.0:0.05557)44.0:0.03017)80.0:0.02518)28.0:0.019
4,(GB_GCA_905181875.1:0.348548,bin.462:0.294326):0.103272)27.0:0.01754)48.0:0.02011)45.0:0.0
2541)'35.0:o__Flavobacteriales':0.02635)14.0:0.02427)66.0:0.02868)61.0:0.03057)83.0:0.03225)39.0
:0.02292)69.0:0.04141)87.0:0.07635)96.0:0.06988,(GB_GCA_013153515.1:0.64155,(GB_GCA_01549
0695.1:0.47791,GB_GCA_011373465.1:0.6252)94.0:0.10188)'97.0:o__SpSt-
205':0.0994)'100.0:c__Bacteroidia':0.17571)100.0:0.06888)82.0:0.03818,((((GB_GCA_011370935.1:0.
32499,(((GB_GCA_003327435.1:0.28288,GB_GCA_003235555.1:0.40685)100.0:0.04621,(GB_GCA_01
8335975.1:0.43241,GB_GCA_016707525.1:0.42764)36.0:0.02476)36.0:0.02362,(GB_GCA_016193365
.1:0.32518,GB_GCA_903958625.1:0.35972)100.0:0.03633)'98.0:c__UBA10030;o__UBA10030':0.0479
4)84.0:0.04103,(GB_GCA_007128495.1:0.34875,RS_GCF_900070205.1:0.36394)40.0:0.03935)79.0:0.
03888,((GB_GCA_018828505.1:0.37605,(GB_GCA_016707285.1:0.6067,(GB_GCA_003599415.1:0.22
678,GB_GCA_016177735.1:0.3051)99.0:0.03242)'100.0:o__Ignavibacteriales':0.12526)100.0:0.09033
,(GB_GCA_017303675.1:0.39726,GB_GCA_016709315.1:0.43527)'100.0:o__SJA-
28':0.17286)'74.0:c__Ignavibacteria':0.04637)80.0:0.03714,((GB_GCA_013152135.1:0.30522,(GB_GC
A_002699105.1:0.36877,GB_GCA_003246455.1:0.30339)100.0:0.07931)'100.0:c__SZUA-
365;o__SZUA-
365':0.12305,(GB_GCA_903900335.1:0.55502,(((((GB_GCA_016788785.1:0.40949,(GB_GCA_0110551
95.1:0.43748,((GB_GCA_015709655.1:0.296294,bin.189:0.191141):0.197936,(GB_GCA_016197185.1
:0.223819,bin.487:0.27046):0.0974612)46.0:0.03134)75.0:0.02261)75.0:0.02759,GB_GCA_00373167
5.1:0.4754)89.0:0.03773,(GB_GCA_002483085.1:0.22508,GB_GCA_002405405.1:0.34049)100.0:0.08
845)85.0:0.04215,(GB_GCA_015490805.1:0.34887,GB_GCA_011373555.1:0.42563)100.0:0.09827)'10
0.0:o__Kapabacteriales':0.10251,GB_GCA_016788565.1:0.43931)100.0:0.07091)'100.0:c__Kapabacte
ria':0.04369)63.0:0.02568)100.0:0.0454)'100.0:p__Bacteroidota':0.07272)77.0:0.04534)40.0:0.02271
)55.0:0.03114)94.0:0.07176)16.0:0.02904,(((GB_GCA_016935765.1:0.484,(GB_GCA_003645115.1:0.4
6633,GB_GCA_003645085.1:0.44136)'100.0:o__UBA6098':0.08576)59.0:0.05385,(GB_GCA_0113715
45.1:0.44646,GB_GCA_002085385.1:0.55739)68.0:0.07101)'100.0:p__UBP14;c__UBA6098':0.17988,(
((GB_GCA_002256535.1:0.66769,(GB_GCA_016208615.1:0.55144,GB_GCA_002366725.1:0.4999)94.
0:0.08117)'100.0:c__UBA3073':0.0984,(GB_GCA_016929715.1:0.94727,(((GB_GCA_011682715.1:0.6
0038,GB_GCA_002441125.1:0.67134)'91.0:c__32-
111':0.08303,GB_GCA_004375965.1:0.54628)68.0:0.05208,(GB_GCA_015486685.1:0.39954,(GB_GC
A_002215665.1:0.58401,GB_GCA_011370805.1:0.47998)'100.0:o__UBA3072':0.07443)'100.0:c__UB
A3072':0.12143)66.0:0.03254)61.0:0.03099)89.0:0.0506,((((GB_GCA_011332965.1:0.56579,(GB_GCA
_015490465.1:0.64629,GB_GCA_015494735.1:0.48265)100.0:0.09573)'87.0:o__LBFQ01':0.06352,((G
B_GCA_015489295.1:0.43083,((GB_GCA_003645975.1:0.32215,(GB_GCA_015489415.1:0.31793,GB_
GCA_011042705.1:0.29541)52.0:0.04496)87.0:0.04961,GB_GCA_002421425.1:0.5988)'73.0:o__UBA
1063':0.03918)59.0:0.04004,GB_GCA_015490255.1:0.57505)77.0:0.04628)100.0:0.06901,GB_GCA_0
02011615.1:0.57437)'100.0:c__Hydrothermia':0.12772,((GB_GCA_003645615.1:0.45209,GB_GCA_00
2049785.1:0.53178)'100.0:o__SM23-

42':0.11212,(GB_GCA_016934635.1:0.74491,((GB_GCA_011363735.1:0.39359,GB_GCA_011358805.1:0.34891)100.0:0.14287,(GB_GCA_016935295.1:0.63503,(GB_GCA_011389195.1:0.26158,GB_GCA_903865935.1:0.41894)99.0:0.07378)100.0:0.06673)'100.0:o__UBA2258':0.07799)99.0:0.06301)'100.0:c__WOR-3':0.108)99.0:0.05152)'98.0:p__WOR-3':0.08414)14.0:0.04462)15.0:0.02795,(((((GB_GCA_000403035.1:0.58904,(((GB_GCA_012961925.1:0.21965,GB_GCA_002049985.1:0.28569)'100.0:c__MVCY01;o__MVCY01':0.13057,(GB_GCA_002059205.1:0.29916,GB_GCA_012961755.1:0.36372)'100.0:c__4484-107':0.07328)81.0:0.04616,(GB_GCA_002070295.1:0.58985,(GB_GCA_018660835.1:0.68013,(GB_GCA_009694805.1:0.31475,(GB_GCA_012271065.1:0.30192,GB_GCA_018650745.1:0.36405)'100.0:o__UBA2968':0.15595)88.0:0.07105)'100.0:c__UBA2968':0.0596)25.0:0.04295)27.0:0.03591)'98.0:p__Latescibacterota':0.04755,(GB_GCA_016209865.1:0.20389,GB_GCA_009841265.1:0.36644)'100.0:p__JAAXHH01;c__JAAXHH01;o__JAAXHH01':0.25672)56.0:0.04735,(((GB_GCA_004376575.1:0.47742,GB_GCA_001303705.1:0.37167)'99.0:p__TA06;c__DG-26':0.10961,(GB_GCA_002347565.1:0.22624,(GB_GCA_002403115.1:0.25626,GB_GCA_002412795.1:0.30284)34.0:0.03969)'100.0:p__Edwardsbacteria;c__AC1;o__AC1':0.31385)66.0:0.06785,(GB_GCA_001304015.1:0.4372,((GB_GCA_001302725.1:0.3805,((GB_GCA_001775395.1:0.35598,GB_GCA_002085375.1:0.37882)'100.0:o__UBA10806':0.12277,GB_GCA_002791595.1:0.50128)40.0:0.03611)100.0:0.05808,(((GB_GCA_001775355.1:0.2691,(GB_GCA_001304315.1:0.29194,GB_GCA_001304155.1:0.26011)68.0:0.03565)100.0:0.05358,(GB_GCA_011331825.1:0.31922,GB_GCA_000447245.1:0.23134)100.0:0.10037)'100.0:o__MSB-5A5':0.08792,(((GB_GCA_012797915.1:0.38066,(GB_GCA_002838945.1:0.36947,GB_GCA_016213305.1:0.48231)100.0:0.06948)'100.0:o__GN15':0.12373,(GB_GCA_903930455.1:0.45241,(GB_GCA_018820315.1:0.31824,GB_GCA_014730285.1:0.37281)'100.0:o__JAABVY01':0.09692)69.0:0.0418)98.0:0.05533,(GB_GCA_011046605.1:0.42499,GB_GCA_014729565.1:0.48395)'84.0:o__DSPP01':0.06932)81.0:0.0382)69.0:0.03261)'100.0:p__Zixibacteria;c__MSB-5A5':0.09838)17.0:0.02853)9.0:0.02897)6.0:0.02355,(GB_GCA_002084765.1:0.49504,GB_GCA_003697105.1:0.44824)'100.0:p__4572-55;c__4572-55':0.11875)19.0:0.01964,(((GB_GCA_001780825.1:0.52211,(GB_GCA_013696835.1:0.52668,(((GB_GCA_013361935.1:0.215903,bin.555:0.265986):0.199797,(GB_GCA_003157095.1:0.33679,GB_GCA_016177465.1:0.36238)100.0:0.07361)'100.0:o__Gemmatimonadales':0.10336,(((GB_GCA_011055965.1:0.33327,GB_GCA_902805735.1:0.34915)100.0:0.05775,(GB_GCA_003242735.1:0.25754,GB_GCA_009843625.1:0.51559)99.0:0.03496)'100.0:o__Longimicrobiales':0.04022,(GB_GCA_012270965.1:0.45573,GB_GCA_011774835.1:0.34178)'100.0:o__KS3-K002':0.05452)100.0:0.05464)100.0:0.10766)'100.0:c__Gemmatimonadetes':0.08048)'100.0:p__Gemmatimonadota':0.08508,(GB_GCA_002686955.1:0.59881,(GB_GCA_016928395.1:0.63528,GB_GCA_002436025.1:0.97233)'99.0:p__Fermentibacterota':0.10928)16.0:0.04758)22.0:0.03776,(((GB_GCA_011772205.1:0.43001,(GB_GCA_014730075.1:0.33446,GB_GCA_002085285.1:0.31552)'100.0:o__Krumholzibacteriales':0.16976)100.0:0.10076,(GB_GCA_016873475.1:0.57532,GB_GCA_903847545.1:0.66988)99.0:0.07406)'100.0:p__Krumholzibacteriota;c__Krumholzibacteria':0.07618,((((GB_GCA_014729455.1:0.38005,GB_GCA_016712505.1:0.38987)100.0:0.08371,(GB_GCA_018831195.1:0.41477,GB_GCA_016867695.1:0.42939)100.0:0.0783)'100.0:o__CAIMUX01':0.16819,(GB_GCA_013003415.1:0.51835,(GB_GCA_016928935.1:0.42165,(GB_GCA_005893165.1:0.54716,(GB_GCA_016235265.1:0.42086,GB_GCA_016223045.1:0.48928)'100.0:o__RBG-16-71-46':0.0637)99.0:0.05794)62.0:0.03085)89.0:0.03744)'98.0:p__Eisenbacteria;c__RBG-16-71-46':0.06118,(GB_GCA_016867725.1:0.50952,(GB_GCA_002686915.1:0.52333,GB_GCA_016930695.1:0.51369)82.0:0.05951)70.0:0.0433)34.0:0.0266)68.0:0.03704)46.0:0.05987)23.0:0.03377)48.0:0.05373)5.0:0.0309,((((GB_GCA_013151025.1:0.61852,(GB_GCA_002319925.1:0.75966,RS_GCF_017377855.1:0.66713)86.0:0.05665)'100.0:c__Holophagae':0.09854,((((((GB_GCA_012798145.1:0.44679,GB_G

CA_011353845.1:0.32026)'100.0:c__UBA4820;o__UBA4820':0.31987,((((GB_GCA_003105185.1:0.45
433,GB_GCA_003158745.1:0.45895)'100.0:o__Thermoanaerobaculales':0.12187,GB_GCA_00232730
5.1:0.63242)73.0:0:0.05813,(GB_GCA_016184115.1:0.48022,(GB_GCA_003222385.1:0.35976,GB_GCA
_017999215.1:0.43117)'100.0:o__UBA5066':0.08675)100.0:0:0.05703)19.0:0:0.03177,(GB_GCA_009837
885.1:0.40734,GB_GCA_016699405.1:0.41118)'100.0:o__UBA5704':0.19395)'100.0:c__Thermoanaer
obaculia':0.08632)15.0:0:0.04332,((GB_GCA_004356015.1:0.48552,((GB_GCA_003695625.1:0.56046,G
B_GCA_014764515.1:0.46127)62.0:0:0.03749,GB_GCA_003223995.1:0.34422)50.0:0:0.02856)83.0:0:0.048
6,(GB_GCA_016195325.1:0.41052,(GB_GCA_003222305.1:0.31852,GB_GCA_016202915.1:0.35163)'
100.0:o__Gp22-
AA2':0.09122)98.0:0:0.06771)'100.0:c__Mor1':0.05188)2.0:0:0.04225,((GB_GCA_903916735.1:0.57747,((
((GB_GCA_016210985.1:0.28842,GB_GCA_011058595.1:0.25882)'100.0:o__HR10':0.1058,(((GB_GCA
_011046985.1:0.20049,(GB_GCA_002483445.1:0.30286,GB_GCA_016705245.1:0.34209)'99.0:o__UB
A7656':0.04788)47.0:0:0.02601,(RS_GCF_018304765.1:0.44765,GB_GCA_016713405.1:0.2976)'98.0:o_
_Chloracidobacteriales':0.05938)87.0:0:0.02316,(GB_GCA_902825875.1:0.38199,GB_GCA_003222855.
1:0.5434)99.0:0:0.04501)100.0:0:0.05377)'100.0:c__Blastocatellia':0.12715,((GB_GCA_003222815.1:0.39
539,GB_GCA_009841055.1:0.40121)100.0:0:0.05518,((((GB_GCA_016191085.1:0.38675,(GB_GCA_004
299485.1:0.40085,(((GB_GCA_003225175.1:0.17547,RS_GCF_009765825.1:0.46683)44.0:0:0.0425,((GB
_GCA_003225655.1:0.15102,GB_GCA_016185395.1:0.16822)57.0:0:0.02554,(GB_GCA_003223515.1:0.
17128,GB_GCA_011046795.1:0.1848)26.0:0:0.02005)25.0:0:0.01938)26.0:0:0.01803,((GB_GCA_01328968
5.1:0.29675,GB_GCA_019236695.1:0.22393)24.0:0:0.01483,GB_GCA_903832295.1:0.26849)42.0:0:0.015
84)100.0:0:0.14949)'99.0:o__Acidobacteriales':0.06177)37.0:0:0.03061,(GB_GCA_019233375.1:0.62744,
(GB_GCA_016201185.1:0.21463,GB_GCA_009841225.1:0.5657)70.0:0:0.03593)'100.0:o__Bryobacteral
es':0.10609)87.0:0:0.03091,(GB_GCA_016210855.1:0.32975,GB_GCA_003153585.1:0.47085)'100.0:o_
_Acidoferrales':0.08743)31.0:0:0.03203,GB_GCA_003224635.1:0.42152)100.0:0:0.07226)'100.0:c__Acid
obacteriae':0.04807)99.0:0:0.03465,(((GB_GCA_016210925.1:0.32704,(GB_GCA_903847975.1:0.42337
,(GB_GCA_016179055.1:0.36567,GB_GCA_016934555.1:0.48223)68.0:0:0.0448)94.0:0:0.03797)69.0:0:0.0
36,(GB_GCA_016211105.1:0.33903,GB_GCA_016211085.1:0.37743)'100.0:o__RPQK01':0.08207)93.0
:0.03731,GB_GCA_016935015.1:0.5494)'100.0:c__UBA6911':0.0481)100.0:0:0.03415)90.0:0:0.03147,((G
B_GCA_004356105.1:0.26683,GB_GCA_009835985.1:0.56908)'100.0:o__Bin61':0.1812,(((GB_GCA_0
03509065.1:0.3529,GB_GCA_009861545.1:0.31715)98.0:0:0.03176,(GB_GCA_016190995.1:0.18883,(((
GB_GCA_016871275.1:0.127382,bin.283:0.101445):0.233138,(GB_GCA_013813355.1:0.2705,((RS_G
CF_001618865.1:0.31464,GB_GCA_017860085.1:0.21052)86.0:0:0.0361,(GB_GCA_005799825.1:0.107
731,bin.564:0.0830829):0.279709)48.0:0:0.03002)21.0:0:0.02556)17.0:0:0.02631,(GB_GCA_016210935.1:
0.22444,GB_GCA_003170135.1:0.21281)87.0:0:0.02892)10.0:0:0.01403)47.0:0:0.02996)'100.0:o__Vicinam
ibacterales':0.20715,(GB_GCA_017990495.1:0.42071,GB_GCA_016934575.1:0.37515)'100.0:o__Fen-
336':0.16907)86.0:0:0.04558)'94.0:c__Vicinamibacteria':0.0578)52.0:0:0.03707)1.0:0:0.03685,((GB_GCA_0
12103255.1:0.32237,GB_GCA_002731215.1:0.32726)'100.0:c__UBA890;o__UBA890':0.28932,(GB_G
CA_016932915.1:0.53573,GB_GCA_902505605.1:0.37896)'100.0:c__B3-
B38':0.07914)44.0:0:0.04475)7.0:0:0.03735,(GB_GCA_002898535.1:0.73419,((GB_GCA_011333655.1:0.4
1054,(GB_GCA_002010665.1:0.25058,((GB_GCA_011389315.1:0.30043,((GB_GCA_014360005.1:0.32
788,GB_GCA_001775755.1:0.28053)63.0:0:0.04911,(GB_GCA_016934895.1:0.27892,(GB_GCA_016933
515.1:0.32851,GB_GCA_003141555.1:0.29104)56.0:0:0.0353)28.0:0:0.02911)17.0:0:0.02644)94.0:0:0.0433,(
GB_GCA_011042725.1:0.30226,(GB_GCA_004377005.1:0.144162,bin.464:0.111442):0.154138)54.0:
0.03683)'100.0:o__Aminicenantales':0.11933)100.0:0:0.08771)98.0:0:0.07316,(GB_GCA_015486055.1:0.
56393,GB_GCA_018897435.1:0.77609)98.0:0:0.08584)'100.0:c__Aminicenantia':0.08982)83.0:0:0.07038
)32.0:0:0.03571)'100.0:p__Acidobacteriota':0.06543,((RS_GCF_014203025.1:0.62381,(GB_GCA_00240
2635.1:0.41925,((RS_GCF_008362905.1:0.2574,(GB_GCA_002338145.1:0.2931,RS_GCF_000183405.1
:0.28637)93.0:0:0.04672)63.0:0:0.0341,(RS_GCF_004087915.1:0.35892,RS_GCF_000487995.1:0.44472)5

3.0:0.03947)56.0:0.05557)'100.0:p__Deferribacterota;c__Deferribacteres;o__Deferribacterales':0.24
635)98.0:0.10235,((GB_GCA_003648675.1:0.30945,GB_GCA_015494645.1:0.38278)'100.0:p__Therm
osulfidibacterota;c__Thermosulfidibacteria;o__Thermosulfidibacterales':0.19751,((GB_GCA_015661
865.1:0.47471,RS_GCF_000191045.1:0.34675)'100.0:c__Desulfurobacteriia;o__Desulfurobacteriales'
:0.15365,(GB_GCA_015490675.1:0.45641,(GB_GCA_003972855.1:0.41722,(GB_GCA_002341805.1:0.
48734,GB_GCA_018771605.1:0.44766)86.0:0.05874)'100.0:o__Aquificales':0.14207)'100.0:c__Aquifi
cae':0.15075)'100.0:p__Aquificota':0.12676)49.0:0.05709)18.0:0.0518)0.0:0.02202,((((((GB_GCA_003
220475.1:0.49672,(GB_GCA_016177865.1:0.2326,GB_GCA_016188625.1:0.17007)84.0:0.03917)'100.
0:o__Rokubacteriales':0.23842,(GB_GCA_011359065.1:0.34898,(GB_GCA_016212365.1:0.22217,((G
B_GCA_016180705.1:0.24577,GB_GCA_001443495.1:0.28347)100.0:0.07376,(GB_GCA_016199025.1
:0.27764,(GB_GCA_016209735.1:0.34107,GB_GCA_016200385.1:0.27966)83.0:0.03631)91.0:0.02932
)85.0:0.03099)'83.0:o__Methylomirabilales':0.04647)100.0:0.10233)'100.0:p__Methylomirabilota;c_
_Methylomirabilia':0.07248,((GB_GCA_011330975.1:0.23691,GB_GCA_002748425.1:0.40534)'100.0:
p__Moduliflexota;c__Moduliflexia;o__Moduliflexales':0.17516,((GB_GCA_011338205.1:0.29451,GB_
GCA_016934595.1:0.37879)'100.0:o__DTGQ01':0.1602,(GB_GCA_003230535.1:0.39602,GB_GCA_01
6235675.1:0.39265)100.0:0.10543)'100.0:p__SZUA-182;c__SZUA-
182':0.0411)67.0:0.02566)83.0:0.03146,(((GB_GCA_001790645.1:0.30388,GB_GCA_003695725.1:0.4
2392)'100.0:p__Schekmanbacteria;c__GWA2-38-11;o__GWA2-38-
11':0.23546,(((((GB_GCA_016176845.1:0.26368,GB_GCA_001803565.1:0.28438)'100.0:p__Nitrospin
ota_B;c__2-12-FULL-45-22;o__2-12-FULL-45-
22':0.13087,(GB_GCA_003696125.1:0.30619,GB_GCA_000522425.1:0.46871)'100.0:p__Tectomicrobi
a;c__Entotheonellia':0.07527)87.0:0.03575,(GB_GCA_016193065.1:0.35359,GB_GCA_016192455.1:0
.42561)73.0:0.05788)48.0:0.02674,((GB_GCA_002377645.1:0.40089,GB_GCA_018669005.1:0.36878)
'100.0:o__UBA8248':0.23625,GB_GCA_016190025.1:0.39501)'81.0:p__UBA8248;c__UBA8248':0.102
98)81.0:0.03781,(((GB_GCA_016212295.1:0.42083,(GB_GCA_012961055.1:0.274813,bin.110:0.1637
02):0.345927)91.0:0.06386,((GB_GCA_018830405.1:0.46972,GB_GCA_001804915.1:0.26795)93.0:0.
07674,GB_GCA_016212345.1:0.33179)'79.0:c__UBA9942':0.05622)73.0:0.03695,((GB_GCA_0162122
75.1:0.48893,(GB_GCA_016207925.1:0.3157,(GB_GCA_015229165.1:0.33398,GB_GCA_016208975.1
:0.31292)56.0:0.03826)'100.0:o__UBA7883':0.18515)'99.0:c__UBA7883':0.06617,GB_GCA_01523181
5.1:0.71837)56.0:0.06831)'59.0:p__Nitrospinota':0.03554)62.0:0.03806)28.0:0.02421,((((GB_GCA_01
6217625.1:0.33979,GB_GCA_001803795.1:0.38152)'100.0:c__RBG-16-64-
22':0.09698,((GB_GCA_003454665.1:0.38992,GB_GCA_016212105.1:0.33359)'94.0:c__UBA9217':0.0
553,((GB_GCA_016212095.1:0.43917,((((GB_GCA_002451135.1:0.30541,GB_GCA_016208845.1:0.24
134)100.0:0.03448,GB_GCA_016207865.1:0.2319)100.0:0.06489,GB_GCA_016214385.1:0.19476)'10
0.0:o__UBA6902':0.07415,((((GB_GCA_002011815.1:0.19107,GB_GCA_002011795.1:0.42176)56.0:0.
03668,(GB_GCA_003453735.1:0.22743,(GB_GCA_003535475.1:0.27144,(GB_GCA_016212245.1:0.16
95,GB_GCA_001803645.1:0.34172)85.0:0.02934)85.0:0.02603)34.0:0.02433)75.0:0.03934,(RS_GCF_
000020985.1:0.41989,(GB_GCA_013349595.1:0.35391,GB_GCA_903822005.1:0.32174)72.0:0.03187)
100.0:0.06134)66.0:0.02488,((GB_GCA_002897855.1:0.32526,GB_GCA_015492515.1:0.31361)46.0:0
.03134,GB_GCA_015233465.1:0.4495)100.0:0.03804)'100.0:o__Thermodesulfovibrionales':0.06795)
100.0:0.05666)100.0:0.07872,GB_GCA_016212195.1:0.31696)'76.0:c__Thermodesulfovibrionia':0.04
511)36.0:0.02258)65.0:0.02657,((GB_GCA_016212085.1:0.60797,((((GB_GCA_002328825.1:0.51343,
(GB_GCA_005877815.1:0.34622,(GB_GCA_005877775.1:0.25179,GB_GCA_003233615.1:0.49101)10
0.0:0.08606)100.0:0.05323)'100.0:o__Nitrospirales':0.06488,GB_GCA_016198945.1:0.35141)87.0:0.0
3458,(GB_GCA_016201825.1:0.30795,GB_GCA_016201765.1:0.41559)84.0:0.07057)62.0:0.03079,((G
B_GCA_016201875.1:0.43484,(((GB_GCA_005239595.1:0.27405,GB_GCA_016200325.1:0.16522)53.
0:0.02873,GB_GCA_012960925.1:0.24364)86.0:0.03394,(GB_GCA_016195485.1:0.36394,GB_GCA_0
05239745.1:0.41049)73.0:0.04394)'100.0:o__SBBL01':0.14372)68.0:0.03364,GB_GCA_016234185.1:

0.3415)37.0:0:0.02445)'75.0:c__Nitrospiria':0.02562)64.0:0:0.04175,(GB_GCA_016214375.1:0.40553,GB
_GCA_001803815.1:0.3359)'95.0:c__9FT-COMBO-42-
15':0.05568)60.0:0.05308)'64.0:p__Nitrospirota':0.0552,(GB_GCA_001873295.1:0.61281,GB_GCA_0
13151235.1:0.38404)74.0:0:0.10007)49.0:0:0.03868)1.0:0:0.01611)18.0:0:0.02801,(GB_GCA_016929465.1:0.
70749,(GB_GCA_016188575.1:0.61762,((GB_GCA_016932295.1:0.51404,(GB_GCA_003558985.1:0.4
7053,GB_GCA_016927905.1:0.33004)'100.0:o__CSSED10-310':0.23144)'95.0:c__CSSED10-
310':0.0741,GB_GCA_016934675.1:0.57518)49.0:0:0.04333)'99.0:p__CSSED10-
310':0.06925)82.0:0:0.05449)2.0:0:0.01945,((((((GB_GCA_015222755.1:0.41873,((GB_GCA_002298925.1
:0.29897,(RS_GCF_017338855.1:0.23272,GB_GCA_903857795.1:0.20699)94.0:0:0.0304)'100.0:o__Geo
bacterales':0.12244,(GB_GCA_017552515.1:0.41636,(((GB_GCA_012974435.1:0.26936,(GB_GCA_00
2868925.1:0.21842,RS_GCF_002898515.1:0.22075)53.0:0:0.0213)70.0:0:0.01754,((GB_GCA_001751155.
1:0.31464,(GB_GCA_016744675.1:0.33894,(GB_GCA_012974445.1:0.23907,GB_GCA_002869605.1:0
.20684)85.0:0:0.03159)57.0:0:0.0204)76.0:0:0.01834,(GB_GCA_007134025.1:0.25995,((((RS_GCF_0004722
85.1:0.11718,(RS_GCF_001611275.1:0.13745,(GB_GCA_014859605.1:0.237,GB_GCA_014860175.1:0.
14751)52.0:0:0.02318)39.0:0:0.01674)21.0:0:0.02561,((RS_GCF_001278055.1:0.12885,RS_GCF_00973135
5.2:0.1485)79.0:0:0.02034,GB_GCA_001825565.1:0.14843)69.0:0:0.01667)5.0:0:0.00903,GB_GCA_002869
635.1:0.20241)8.0:0:0.01166,GB_GCA_002868845.1:0.14705)53.0:0:0.0168)75.0:0:0.01853)60.0:0:0.02119)6
5.0:0:0.01702,RS_GCF_000827125.1:0.21114)100.0:0:0.04103)'100.0:o__Desulfuromonadales':0.1016)'1
00.0:c__Desulfuromonadia':0.08852)90.0:0:0.03426,GB_GCA_016208525.1:0.36733)80.0:0:0.0399,(GB_
GCA_016183025.1:0.41319,(GB_GCA_015492305.1:0.32937,((GB_GCA_015492395.1:0.30345,((GB_
GCA_015492355.1:0.21176,GB_GCA_015492495.1:0.23895)93.0:0:0.03739,(GB_GCA_011375175.1:0.2
9635,(GB_GCA_015222595.1:0.31254,(GB_GCA_016234935.1:0.22855,GB_GCA_016182705.1:0.300
26)96.0:0:0.04378)100.0:0:0.05114)38.0:0:0.02248)'100.0:o__GWC2-55-
46':0.06387)73.0:0:0.02508,(GB_GCA_016214995.1:0.24197,GB_GCA_016234925.1:0.23645)'100.0:o_
_UBA9637':0.07459)100.0:0:0.08055)'100.0:c__GWC2-55-
46':0.0752)23.0:0:0.0254)36.0:0:0.02827,(((GB_GCA_011370045.1:0.52579,(GB_GCA_016933535.1:0.22
662,(GB_GCA_016929395.1:0.21358,GB_GCA_016931225.1:0.23627)56.0:0:0.04497)'100.0:c__Zymog
enia;o__Zymogeniales':0.24121)41.0:0:0.04579,(GB_GCA_903896745.1:0.5673,GB_GCA_016929755.1:
0.61653)'78.0:c__Desulfomonilia':0.07402)8.0:0:0.03396,((((((((GB_GCA_013374135.1:0.48995,GB_GC
A_018830135.1:0.2497)'100.0:o__Adiutricales':0.16442,(GB_GCA_018812395.1:0.40431,GB_GCA_01
6218705.1:0.43743)'100.0:o__Desulfarculales':0.08538)'98.0:c__Desulfarculia':0.0597,(GB_GCA_011
334855.1:0.32461,RS_GCF_000195295.1:0.29195)'100.0:c__Desulfobaccia;o__Desulfobaccales':0.20
463)71.0:0:0.04344,((GB_GCA_001304365.1:0.28627,RS_GCF_001577525.1:0.24018)'100.0:c__Desulfo
fervidia;o__Desulfofervidales':0.25048,((((GB_GCA_011048585.1:0.330017,bin.21:0.21882):0.055793
,(GB_GCA_002868955.1:0.27736,(GB_GCA_011051825.1:0.33472,GB_GCA_019163335.1:0.40733)10
0.0:0:0.0694)86.0:0:0.035)87.0:0:0.05945,GB_GCA_015488275.1:0.23835)'100.0:c__Desulfobulbia;o__Des
ulfobulbales':0.18603,(((GB_GCA_015491585.1:0.26636,(((GB_GCA_011042225.1:0.29461,GB_GCA_
002419025.1:0.32033)57.0:0:0.02827,GB_GCA_015493475.1:0.39434)68.0:0:0.03999,RS_GCF_01297923
5.1:0.2633)72.0:0:0.03756)51.0:0:0.02905,GB_GCA_015494155.1:0.32596)'100.0:c__Dissulfuribacteria;o
__Dissulfuribacterales':0.12444,(RS_GCF_011207455.1:0.24679,(RS_GCF_000421585.1:0.26862,RS_
GCF_000421605.1:0.56577)100.0:0:0.12469)'100.0:c__Thermodesulfobacteria;o__Thermodesulfobact
eriales':0.11148)100.0:0:0.06885)89.0:0:0.04921)42.0:0:0.0322)15.0:0:0.02546,(((GB_GCA_012962595.1:0.3
1978,GB_GCA_011046395.1:0.30733)75.0:0:0.08261,((GB_GCA_002899795.1:0.29897,(GB_GCA_0036
46715.1:0.27055,(RS_GCF_900114975.1:0.3247,(GB_GCA_001603845.1:0.36678,RS_GCF_900176285
.1:0.27531)100.0:0:0.07083)'100.0:o__Syntrophobacterales':0.08128)97.0:0:0.04819)'100.0:c__Syntroph
obacteria':0.07657,(GB_GCA_011334845.1:0.45543,((GB_GCA_003646935.1:0.183905,bin.560:0.210
749):0.213095,((((GB_GCA_016930835.1:0.231698,bin.267:0.184804):0.0367066,bin.269:0.196445):
0.00983826,bin.191:0.211558):0.0490071,GB_GCA_001874005.1:0.43497)98.0:0:0.07254)45.0:0:0.0540

2)'100.0:c__DSM-
4660;o__Desulfatiglandales':0.12646)11.0:0.02197)6.0:0.02158,((GB_GCA_011334425.1:0.43888,(GB
_GCA_015233055.1:0.40841,GB_GCA_016208165.1:0.38604)47.0:0.04369)63.0:0.0408,((GB_GCA_00
1751015.1:0.3342,(GB_GCA_012961955.1:0.23179,GB_GCA_013375375.1:0.27667)91.0:0.04125)'97.
0:o__C00003060':0.04381,(GB_GCA_018829095.1:0.43627,(GB_GCA_002753725.1:0.38575,(((((GB_
GCA_018816735.1:0.27046,((GB_GCA_002748835.1:0.30397,RS_GCF_007830435.1:0.36347)51.0:0.0
294,(RS_GCF_900101345.1:0.31263,(GB_GCA_002084385.1:0.30463,GB_GCA_011390695.1:0.53472
)86.0:0.04562)83.0:0.03054)95.0:0.0364)97.0:0.04173,(RS_GCF_000018405.1:0.36963,GB_GCA_013
151835.1:0.39375)99.0:0.03513)71.0:0.02045,(GB_GCA_015231595.1:0.37932,GB_GCA_013824585.
1:0.26281)45.0:0.02049)54.0:0.01796,(GB_GCA_903839535.1:0.34272,((((RS_GCF_017377395.1:0.14
616,GB_GCA_011389735.1:0.31249)46.0:0.04784,RS_GCF_003851005.1:0.25878)36.0:0.02588,((((((
GB_GCA_016735415.1:0.29669,(GB_GCA_011042305.1:0.26287,GB_GCA_016219005.1:0.29653)51.
0:0.04019)36.0:0.03103,(GB_GCA_905176665.1:0.21075,GB_GCA_016342285.1:0.20432)87.0:0.0492
8)25.0:0.02522,(GB_GCA_011390985.1:0.24753,GB_GCA_002402905.1:0.35103)25.0:0.02267)14.0:0
.01911,(GB_GCA_903231505.1:0.22934,GB_GCA_001303025.1:0.22134)66.0:0.04875)6.0:0.01492,(G
B_GCA_003973265.1:0.31377,GB_GCA_003647375.1:0.23206)8.0:0.04065)10.0:0.01616,GB_GCA_00
3599475.1:0.29375)17.0:0.02215,(GB_GCA_002084545.1:0.2246,(GB_GCA_018432925.1:0.21104,((((
GB_GCA_013791935.1:0.30536,GB_GCA_002771315.1:0.33465)11.0:0.02412,(GB_GCA_011391835.
1:0.22999,GB_GCA_003818505.1:0.22735)48.0:0.0386)17.0:0.0145,((GB_GCA_013792055.1:0.18828
5,bin.139:0.147452):0.0097918,bin.334:0.295667):0.0364229)12.0:0.01709,(RS_GCF_000472805.1:0.
26422,(GB_GCA_001751005.1:0.138155,bin.469:0.157528):0.0442447)14.0:0.01543)12.0:0.01428)15
.0:0.01803)10.0:0.01844)1.0:0.0145)0.0:0.01233,(GB_GCA_002347265.1:0.24809,GB_GCA_0113897
45.1:0.29469)39.0:0.02958)23.0:0.01814)32.0:0.02275)62.0:0.03818,GB_GCA_900659855.1:0.36156)
98.0:0.03005)60.0:0.0259)'100.0:o__Desulfobacterales':0.06567)'100.0:c__Desulfobacteria':0.12813)
24.0:0.02479)17.0:0.02106)90.0:0.04944,((GB_GCA_016935565.1:0.22774,(GB_GCA_018433345.1:0.
382962,bin.326:0.223478):0.0386376)95.0:0.03731,(GB_GCA_002327315.1:0.32708,(GB_GCA_0152
22855.1:0.18636,(GB_GCA_016930075.1:0.26245,((GB_GCA_003161855.1:0.1686,(GB_GCA_001873
675.1:0.19839,GB_GCA_003142295.1:0.21099)36.0:0.01508)28.0:0.01846,((((GB_GCA_016209105.1:
0.18724,GB_GCA_001873745.1:0.23057)36.0:0.02666,(GB_GCA_012522415.1:0.29201,GB_GCA_002
067815.1:0.23965)10.0:0.02685)0.0:0.01446,(GB_GCA_002403385.1:0.20981,GB_GCA_018056685.1:
0.44977)28.0:0.03064)4.0:0.02063,(GB_GCA_016929555.1:0.2125,(((GB_GCA_011333305.1:0.28043,
GB_GCA_011333345.1:0.29227)54.0:0.07833,(GB_GCA_002841865.1:0.22922,GB_GCA_903885795.
1:0.23472)52.0:0.02929)26.0:0.02997,(GB_GCA_002347815.1:0.2096,GB_GCA_002428445.1:0.22765
)31.0:0.03342)3.0:0.01849)1.0:0.01463)0.0:0.0195)0.0:0.01242)39.0:0.03863)2.0:0.01855)20.0:0.029
23)'100.0:c__Syntrophia;o__Syntrophales':0.24135)41.0:0.02623,((GB_GCA_017996625.1:0.44059,(
GB_GCA_013139225.1:0.334,(GB_GCA_011328795.1:0.27853,GB_GCA_016875025.1:0.41857)'100.0
:o__CAIYCZ01':0.12238)100.0:0.0537)'100.0:c__WTBG01':0.07233,(GB_GCA_001304105.1:0.39317,G
B_GCA_009773075.1:0.31959)72.0:0.04873)55.0:0.03838)3.0:0.01412,(((GB_GCA_011773445.1:0.30
422,(GB_GCA_011334915.1:0.1745,GB_GCA_011333355.1:0.23055)'100.0:o__BSN033':0.14753)100.
0:0.0616,(GB_GCA_003646745.1:0.20791,GB_GCA_003646705.1:0.30148)'100.0:o__B13-
G15':0.12075)'88.0:c__BSN033':0.05528,GB_GCA_011327825.1:0.50575)14.0:0.02931)14.0:0.02463)
2.0:0.02204)'6.0:p__Desulfobacterota':0.02876,(((GB_GCA_016202395.1:0.50588,(((GB_GCA_01135
8755.1:0.327842,bin.441:0.293987):0.157408,((GB_GCA_016180865.1:0.40289,(GB_GCA_00279632
5.1:0.39287,GB_GCA_016183065.1:0.54606)100.0:0.06945)'88.0:o__GCA-
002796325':0.0507,(GB_GCA_001798075.1:0.42892,((GB_GCA_016927275.1:0.261202,bin.214:0.234
162):0.105348,GB_GCA_016202335.1:0.41615)'100.0:o__2-02-FULL-44-
16':0.08323)100.0:0.0535)69.0:0.03939)100.0:0.07593,((GB_GCA_001798715.1:0.41139,(GB_GCA_0
16177765.1:0.32869,(GB_GCA_016699445.1:0.3334,GB_GCA_016180855.1:0.34671)100.0:0.06686)5

7.0:0.05002)'100.0:o__2-02-FULL-50-
16':0.10023,((GB_GCA_018812345.1:0.52741,(GB_GCA_005240075.1:0.32798,(GB_GCA_001797365.
1:0.25946,GB_GCA_001798135.1:0.32294)93.0:0.06313)100.0:0.07839)'100.0:o__UBA10199':0.1184
8,(GB_GCA_015233135.1:0.37687,(GB_GCA_011331325.1:0.26406,GB_GCA_016183235.1:0.38664)1
00.0:0.07549)'100.0:o__DSSB01':0.09247)85.0:0.03849)84.0:0.03838)'100.0:p__UBA10199;c__UBA1
0199':0.07541)40.0:0.03051,(((GB_GCA_016182335.1:0.58585,GB_GCA_001798105.1:0.54882)97.0:
0.06319,(GB_GCA_001798265.1:0.46226,GB_GCA_016205125.1:0.50103)98.0:0.07674)'84.0:p__Bdel
lovibrionota_E;c__O2-12-FULL-43-
9':0.03126,GB_GCA_016926495.1:0.72177)18.0:0.06305)16.0:0.03133,(((GB_GCA_002328485.1:0.29
608,GB_GCA_002067165.1:0.32394)'100.0:p__Desulfobacterota_G;c__Syntrophorhabdia;o__Syntro
phorhabdales':0.34468,(GB_GCA_011047235.1:0.49647,GB_GCA_004195025.1:0.6584)56.0:0.0799)3
3.0:0.06195,(GB_GCA_905181755.1:0.82046,((GB_GCA_004356685.1:0.33732,(GB_GCA_902826595.
1:0.26097,GB_GCA_001443445.1:0.27512)100.0:0.04584)'100.0:o__UBA2774':0.0494,(GB_GCA_007
571325.1:0.32393,GB_GCA_014075295.1:0.4881)'76.0:o__RKRQ01':0.07728)100.0:0.06702)'100.0:p
__Desulfobacterota_D;c__UBA1144':0.19506)17.0:0.04602)1.0:0.0304)0.0:0.03204,((((GB_GCA_0028
69005.1:0.30731,(GB_GCA_016214985.1:0.20872,RS_GCF_000526155.1:0.19431)37.0:0.06049)'100.
0:c__Deferrisomatia;o__Deferrisomatales':0.25468,(GB_GCA_015493505.1:0.45378,(GB_GCA_01548
6505.1:0.35853,GB_GCA_016933675.1:0.34255)'100.0:c__Anaeroferrophillalia;o__Anaeroferrophilla
les':0.2098)57.0:0.06053)'10.0:p__Desulfobacterota_C':0.03689,((GB_GCA_016219515.1:0.4377,GB_
GCA_011773515.1:0.41912)'100.0:p__Desulfobacterota_E;c__MBNT15':0.18132,(GB_GCA_0169371
35.1:0.2176,GB_GCA_003228585.1:0.2575)'100.0:p__BMS3Abin14;c__BMS3Abin14;o__BMS3Abin14
':0.28975)14.0:0.05449)5.0:0.03218,(((GB_GCA_016713535.1:0.53683,GB_GCA_012517325.1:0.5458
1)31.0:0.09013,((GB_GCA_903893315.1:0.56475,(((GB_GCA_011334465.1:0.54153,GB_GCA_009885
395.1:0.62737)21.0:0.04357,((GB_GCA_016189835.1:0.35656,(GB_GCA_005879795.1:0.35,GB_GCA_
016200545.1:0.31627)'60.0:o__UTPRO1':0.03585)35.0:0.0307,((GB_GCA_902826395.1:0.32495,(GB_
GCA_016210005.1:0.22983,(GB_GCA_017860575.1:0.18268,(GB_GCA_016200585.1:0.16892,GB_GC
A_002898795.1:0.33649)44.0:0.0317)93.0:0.04608)90.0:0.04319)'100.0:o__HRBIN30':0.10852,GB_G
CA_013140885.1:0.47019)19.0:0.0268)19.0:0.02745)28.0:0.04617,GB_GCA_001798595.1:0.43236)8
3.0:0.0491)'100.0:p__Desulfobacterota_B;c__Binatia':0.07693,GB_GCA_016190245.1:0.74291)4.0:0.
03072)1.0:0.03871,((((GB_GCA_006226375.1:0.61555,(GB_GCA_013152945.1:0.49967,GB_GCA_003
598065.1:0.49894)84.0:0.08271)5.0:0.03781,((((GB_GCA_007128675.1:0.47623,((GB_GCA_00587909
5.1:0.41619,RS_GCF_000013385.1:0.37291)93.0:0.04803,(RS_GCF_001263175.1:0.32573,(GB_GCA_
002408385.1:0.29551,(GB_GCA_018266075.1:0.28768,RS_GCF_003600865.1:0.36853)99.0:0.0378)9
9.0:0.03917)84.0:0.03155)'100.0:o__Myxococcales':0.07338)'100.0:c__Myxococcia':0.08849,(((GB_G
CA_002722845.1:0.76108,((GB_GCA_016218805.1:0.45118,((GB_GCA_002721815.1:0.35007,GB_GC
A_016000045.1:0.48547)77.0:0.05144,GB_GCA_016874845.1:0.3864)'96.0:c__UBA727;o__UBA727':
0.14124)48.0:0.04032,GB_GCA_016934455.1:0.37456)91.0:0.05785)62.0:0.04258,GB_GCA_0167134
25.1:0.61588)56.0:0.03678,(GB_GCA_016283195.1:0.64472,((GB_GCA_016931625.1:0.29098,GB_GC
A_001798605.1:0.30779)97.0:0.05594,(GB_GCA_018668695.1:0.33675,GB_GCA_005801425.1:0.396
46)51.0:0.0534)'100.0:o__XYA12-FULL-58-9':0.15325)'86.0:c__XYA12-FULL-58-
9':0.08951)75.0:0.02951)21.0:0.0265,(GB_GCA_016192705.1:0.53716,(GB_GCA_012798435.1:0.546
98,(GB_GCA_011373705.1:0.51476,GB_GCA_016218965.1:0.34061)'100.0:c__SpSt-1050;o__SpSt-
1050':0.15975)100.0:0.06271)19.0:0.03789)60.0:0.03833,(((((GB_GCA_003696955.1:0.59939,GB_GC
A_016711215.1:0.45715)47.0:0.04408,(GB_GCA_011390535.1:0.4837,GB_GCA_018821745.1:0.6565
9)'99.0:o__HGW-
17':0.09592)53.0:0.03416,((GB_GCA_016218785.1:0.37466,GB_GCA_003136095.1:0.49199)72.0:0.0
4012,((GB_GCA_016794505.1:0.37371,GB_GCA_009692555.1:0.29598)'100.0:o__Palsa-
1104':0.08617,(GB_GCA_009692535.1:0.29103,(GB_GCA_018262455.1:0.51761,GB_GCA_014238595

.1:0.56953)100.0:0.089)'100.0:o__Haliangiales':0.09121)75.0:0.03845)100.0:0.07037)100.0:0.05824,(
(GB_GCA_011389755.1:0.51322,(GB_GCA_012797735.1:0.47745,GB_GCA_011373655.1:0.63106)'70
.0:o__DRWM01':0.10427)53.0:0.04517,(GB_GCA_016190375.1:0.38796,((GB_GCA_007124745.1:0.5
5085,((GB_GCA_017303575.1:0.33504,GB_GCA_004283055.1:0.38536)95.0:0.02707,(GB_GCA_0169
29805.1:0.38586,(GB_GCA_016699535.1:0.35322,GB_GCA_016712745.1:0.2341)84.0:0.04109)88.0:0
.03832)90.0:0.04888)100.0:0.07782,(GB_GCA_001465015.1:0.49921,(GB_GCA_016709545.1:0.3235
4,GB_GCA_016712525.1:0.41844)100.0:0.1032)97.0:0.0408)'100.0:o__Polyangiales':0.06561)100.0:0
.06946)70.0:0.07224)'70.0:c__Polyangia':0.04764,((((GB_GCA_003559575.1:0.43899,(GB_GCA_9039
23815.1:0.40023,GB_GCA_016875215.1:0.42881)'100.0:o__CAIXZC01':0.2167)78.0:0.05479,(GB_GC
A_002728755.1:0.58683,(GB_GCA_015493765.1:0.52191,GB_GCA_903936705.1:0.53078)'100.0:o__
DTJE01':0.14156)59.0:0.0368)71.0:0.04042,((GB_GCA_018780435.1:0.54888,(GB_GCA_002841945.1:
0.48762,GB_GCA_013360285.1:0.44019)37.0:0.04522)39.0:0.03809,GB_GCA_009692465.1:0.78708)
83.0:0.04755)'100.0:c__UBA9042':0.07129,(GB_GCA_002309015.1:0.64108,(GB_GCA_017613855.1:
0.73151,((GB_GCA_016931535.1:0.48753,(GB_GCA_903912275.1:0.0133725,bin.666:0.0176085):0.5
88867)91.0:0.06433,(GB_GCA_002716585.1:0.42857,GB_GCA_007120915.1:0.72153)'100.0:o__Brad
ymonadales':0.06637)86.0:0.03533)100.0:0.08306)'94.0:c__Bradymonadia':0.04713)64.0:0.0395)25.
0:0.03331)3.0:0.0309)'38.0:p__Myxococcota':0.0336,(GB_GCA_001797815.1:0.31424,GB_GCA_0113
37885.1:0.45139)'100.0:p__RBG-13-61-14;c__RBG-13-61-14;o__RBG-13-61-
14':0.24696)0.0:0.02721,(((GB_GCA_003695505.1:0.68468,((GB_GCA_001311565.1:0.33488,(GB_GC
A_011389765.1:0.4058,((RS_GCF_900104215.1:0.39241,GB_GCA_014804915.1:0.81793)86.0:0.0396
9,((GB_GCA_003551155.1:0.38181,RS_GCF_900104045.1:0.36667)100.0:0.06469,(RS_GCF_0148737
65.1:0.3647,(RS_GCF_001748225.1:0.23433,GB_GCA_009929795.1:0.28575)95.0:0.03956)100.0:0.04
732)98.0:0.03465)49.0:0.0333)48.0:0.07777)'100.0:p__Desulfobacterota_I;c__Desulfovibrionia;o__D
esulfovibrionales':0.25105,(GB_GCA_002347965.1:0.75091,RS_GCF_000755505.1:0.81296)45.0:0.09
341)10.0:0.03421)13.0:0.03582,((((RS_GCF_001399755.1:0.39917,((((GB_GCA_002167745.1:0.43356
,((((RS_GCF_014202905.1:0.17514,((RS_GCF_000428465.1:0.18252,RS_GCF_900115065.1:0.1966)10
0.0:0.03854,((GB_GCA_019232825.1:0.25362,RS_GCF_003201855.1:0.15535)66.0:0.03275,(GB_GCA
_016699915.1:0.4238,(GB_GCA_003963245.1:0.51242,RS_GCF_014054945.1:0.32245)43.0:0.02955)
76.0:0.0254)93.0:0.06221)51.0:0.01987)59.0:0.02905,RS_GCF_000376945.1:0.22951)94.0:0.08102,((
((RS_GCF_000527135.1:0.31315,((((((((RS_GCF_006716905.1:0.160032,bin.87:0.0942452):0.012990
7,bin.25:0.112138):0.0703583,bin.10:0.165243):0.180764,bin.365:0.242259):0.00559792,bin.213:0.2
6374):0.00888309,bin.388:0.229047):0.140124,GB_GCA_016790635.1:0.25622)36.0:0.04026,GB_GC
A_005888955.1:0.35779)16.0:0.02015,(GB_GCA_001303445.1:0.18426,bin.531:0.145727):0.20299)1
3.0:0.02049)9.0:0.02658,(GB_GCA_014379045.1:0.31782,((((GB_GCA_014379915.1:0.1885,GB_GCA_
016791285.1:0.19589)80.0:0.03433,((GB_GCA_903913965.1:0.196782,bin.514:0.176287):0.0558881,
GB_GCA_009377585.1:0.24097)77.0:0.02566)5.0:0.01488,((GB_GCA_004525885.1:0.28331,((((GB_G
CA_003519965.1:0.21941,GB_GCA_018823025.1:0.17601)88.0:0.02752,RS_GCF_004346685.1:0.143
34)77.0:0.01913,(GB_GCA_018781585.1:0.19182,GB_GCA_013140765.1:0.27039)16.0:0.02608)9.0:0
.02965,(GB_GCA_013044025.1:0.19719,GB_GCA_004298605.1:0.25931)85.0:0.02632)9.0:0.02348)1
0.0:0.02407,GB_GCA_016183615.1:0.18086)5.0:0.02025)3.0:0.0183,((GB_GCA_009693245.1:0.2468
3,GB_GCA_001464895.1:0.22983)60.0:0.02422,GB_GCA_001304425.1:0.25099)72.0:0.02749)2.0:0.0
1827)2.0:0.01887)0.0:0.0118,(GB_GCA_903905585.1:0.33385,(RS_GCF_013003985.1:0.32561,GB_G
CA_902826775.1:0.239)55.0:0.03167)43.0:0.02876)1.0:0.02039,(GB_GCA_009923755.1:0.32397,GB_
GCA_016873905.1:0.34638)5.0:0.02109)0.0:0.01913)0.0:0.02355,(GB_GCA_018971485.1:0.28594,(((
GB_GCA_016778465.1:0.195292,bin.678:0.149072):0.20898,bin.196:0.176135):0.0189676,bin.543:0.
253361):0.12092)33.0:0.02884)21.0:0.04618)22.0:0.0345,(GB_GCA_902586195.1:0.72528,GB_GCA_
001460935.1:0.61298)28.0:0.07056)'98.0:o__Burkholderiales':0.12113,(((GB_GCA_014238985.1:0.61
349,((GB_GCA_018664045.1:0.47033,GB_GCA_002480165.1:0.67057)47.0:0.0813,(GB_GCA_002320

385.1:0.73731,(GB_GCA_002323935.1:0.68711,GB_GCA_002690495.1:0.73628)42.0:0:0.07193)39.0:0:0.
04745)54.0:0:0.04532)'99.0:o__UBA7916':0.12781,(GB_GCA_009927065.1:0.82624,GB_GCA_0017506
25.1:0.82754)2.0:0:0.0809)0.0:0:0.09957,(((GB_GCA_002352565.1:0.4756,GB_GCA_011620135.1:0.4929
1)'94.0:o__UBA2770':0.1073,((GB_GCA_007571105.1:0.50659,GB_GCA_007570905.1:0.45006)'96.0:
o__Porisulfidales':0.06382,(GB_GCA_002454015.1:0.55148,(GB_GCA_001750645.1:0.61161,GB_GCA
_014238345.1:0.50861)75.0:0:0.10595)9.0:0:0.03843)11.0:0:0.03597)8.0:0:0.0376,(((((GB_GCA_009937445.
1:0.112357,bin.399:0.0467091):0.362273,GB_GCA_002434915.1:0.43948)51.0:0:0.04839,(GB_GCA_01
8672815.1:0.55192,GB_GCA_009845905.1:0.50784)85.0:0:0.05225)35.0:0:0.06765,((GB_GCA_00217053
5.1:0.35673,GB_GCA_902597385.1:0.30622)100.0:0:0.25354,(((GB_GCA_015163845.1:0.74174,((GB_G
CA_902630715.1:0.54599,GB_GCA_003282105.1:0.5607)100.0:0:0.10222,(GB_GCA_004321845.1:0.74
933,(GB_GCA_902627055.1:0.132936,bin.654:0.113891):0.427994)100.0:0:0.13467)'100.0:o__SAR86':
0.06648)34.0:0:0.04947,(((RS_GCF_000300075.1:0.78391,RS_GCF_002776555.1:0.62694)66.0:0:0.05943,
RS_GCF_012562765.1:0.66683)28.0:0:0.10199,GB_GCA_013215905.1:0.5356)6.0:0:0.04855)1.0:0:0.03485,
(GB_GCA_003645455.1:0.50924,(GB_GCA_002718845.1:0.6436,GB_GCA_002705445.1:0.54677)'94.
0:o__GCA-
002705445':0.06209)52.0:0:0.0445)4.0:0:0.07503)0.0:0:0.08371)0.0:0:0.0297,(((((((GB_GCA_013373515.1:0.
40023,GB_GCA_013001575.1:0.35661)3.0:0:0.0386,GB_GCA_905479545.1:0.32984)10.0:0:0.04308,((GB
_GCA_012513365.1:0.48977,(RS_GCF_009362845.1:0.40539,RS_GCF_001263335.1:0.49949)100.0:0:0.
11446)'98.0:o__Cardiobacteriales':0.06189,((GB_GCA_018623305.1:0.30804,RS_GCF_011398355.1:0
.51402)'99.0:o__Thiomicrospirales':0.05316,GB_GCA_902512305.1:0.67714)74.0:0:0.03423)62.0:0:0.021
15)6.0:0:0.02787,(((RS_GCF_003097315.1:0.29971,RS_GCF_000297215.2:0.34806)62.0:0:0.05458,((((((G
B_GCA_002408105.1:0.31835,GB_GCA_019090315.1:0.27035)95.0:0:0.06778,((GB_GCA_903853385.1:
0.344596,bin.121:0.232575):0.121804,(GB_GCA_002704325.1:0.592517,bin.250:0.249109):0.098823
)47.0:0:0.02812)33.0:0:0.02088,((((RS_GCF_000377745.1:0.30582,((GB_GCA_018069525.1:0.265416,bin.
209:0.227449):0.0635445,GB_GCA_018662305.1:0.35937)98.0:0:0.02458)98.0:0:0.03222,(GB_GCA_016
713115.1:0.27804,RS_GCF_003688415.1:0.27428)28.0:0:0.03282)35.0:0:0.02843,(((GB_GCA_002415025
.1:0.21359,(RS_GCF_900120175.1:0.19421,RS_GCF_003752585.1:0.22565)75.0:0:0.02922)71.0:0:0.0249
9,RS_GCF_000153125.2:0.34475)28.0:0:0.01731,(GB_GCA_902705835.1:0.26794,GB_GCA_905478995.
1:0.34404)47.0:0:0.028)86.0:0:0.02264)3.0:0:0.01887,(GB_GCA_016705365.1:0.29504,GB_GCA_00622708
5.1:0.2542)62.0:0:0.05184)99.0:0:0.02852)37.0:0:0.03001,((RS_GCF_000397065.2:0.33849,((((RS_GCF_014
803385.1:0.33396,RS_GCF_009821115.1:0.36313)25.0:0:0.02038,(GB_GCA_015665455.1:0.26948,(RS_
GCF_000153185.1:0.38594,RS_GCF_000355675.1:0.31072)48.0:0:0.02648)28.0:0:0.01923)25.0:0:0.01615,(
(RS_GCF_000282455.1:0.44938,((RS_GCF_003864255.1:0.15432,RS_GCF_000428585.1:0.19327)100.
0:0.03079,RS_GCF_000710775.1:0.21782)97.0:0:0.0369)88.0:0:0.02301,GB_GCA_001593685.1:0.24707)
39.0:0:0.01968)19.0:0:0.01407,RS_GCF_013697085.1:0.44951)38.0:0:0.01751)41.0:0:0.01457,GB_GCA_9025
41775.1:0.52372)65.0:0:0.01548)39.0:0:0.02331,((RS_GCF_006757745.1:0.57422,(GB_GCA_018262975.
1:0.24221,RS_GCF_000300005.1:0.33345)87.0:0:0.04531)100.0:0:0.04157,(GB_GCA_019090565.1:0.311
75,(GB_GCA_016765265.1:0.23546,GB_GCA_000198515.1:0.316)87.0:0:0.02909)93.0:0:0.02518)91.0:0:0.
02685)37.0:0:0.02203,(GB_GCA_015163815.1:0.45553,GB_GCA_002450655.1:0.43061)86.0:0:0.09914)'4
5.0:o__Pseudomonadales':0.03381,((((RS_GCF_000374105.1:0.30779,GB_GCA_003696665.1:0.3511)
84.0:0:0.0236,RS_GCF_013179735.1:0.34898)88.0:0:0.01968,GB_GCA_002376965.1:0.28988)56.0:0:0.020
11,((GB_GCA_003585935.1:0.6022,RS_GCF_900115655.1:0.47038)95.0:0:0.05753,((((RS_GCF_0037516
25.1:0.21078,GB_GCA_017425245.1:0.29326)49.0:0:0.02353,((GB_GCA_013214945.1:0.24124,RS_GCF
_002282895.1:0.23843)71.0:0:0.02713,RS_GCF_002954545.1:0.35237)13.0:0:0.01153)41.0:0:0.0136,(RS_G
CF_005405585.1:0.22232,(RS_GCF_001574435.1:0.19929,RS_GCF_003596335.1:0.1567)95.0:0:0.0370
4)75.0:0:0.02922)47.0:0:0.01777,((RS_GCF_004551525.1:0.3183,(RS_GCF_000185305.1:0.28496,GB_GC
A_009668015.1:0.26517)36.0:0:0.04816)32.0:0:0.04196,RS_GCF_000243075.1:0.21066)17.0:0:0.01797)32.
0:0.05109)28.0:0:0.08438)'26.0:o__Enterobacterales':0.04004)10.0:0:0.02178)15.0:0:0.02504,(GB_GCA_0

16207405.1:0.56106,((((GB_GCA_903870015.1:0.42766,GB_GCA_905612315.1:0.50244)84.0:0.0497,
RS_GCF_000815225.1:0.63985)100.0:0.09584,(GB_GCA_002708265.1:0.49153,GB_GCA_013288135.
1:0.35267)84.0:0.04031)6.0:0.02454,((GB_GCA_009692385.1:0.37702,(GB_GCA_018061105.1:0.448
77,GB_GCA_013289085.1:0.37771)52.0:0.06566)14.0:0.03569,(((GB_GCA_001801465.1:0.39039,(GB
_GCA_014132615.1:0.49759,(GB_GCA_002469885.1:0.33483,GB_GCA_018061255.1:0.40006)71.0:0.
05823)47.0:0.02709)44.0:0.02513,((GB_GCA_005877695.1:0.5132,GB_GCA_016218405.1:0.39071)9
0.0:0.04495,((GB_GCA_003450655.1:0.5698,GB_GCA_002429595.1:0.36353)53.0:0.02804,((GB_GCA
_018402195.1:0.46898,GB_GCA_003354005.1:0.56204)71.0:0.12496,GB_GCA_003142435.1:0.45461
)11.0:0.02592)28.0:0.03241)5.0:0.01647)6.0:0.02023,(((GB_GCA_012103495.1:0.35861,(RS_GCF_900
639915.1:0.223252,bin.194:0.18907):0.238708)90.0:0.04017,GB_GCA_018241565.1:0.28808)70.0:0.
043,GB_GCA_009885935.1:0.53589)40.0:0.02191)3.0:0.01925)7.0:0.02401)2.0:0.0209)3.0:0.03374)0
.0:0.03435)0.0:0.02065,(((GB_GCA_004796385.1:0.34113,(GB_GCA_014384675.1:0.32392,RS_GCF_0
16097415.1:0.36036)'44.0:o__GCF-
002020875':0.03579)24.0:0.02949,((GB_GCA_002733135.1:0.38286,(GB_GCA_003646305.1:0.24191,
(GB_GCA_011322755.1:0.36647,((GB_GCA_002352385.1:0.20943,GB_GCA_003666325.1:0.37637)45
.0:0.01742,(RS_GCF_900155475.1:0.3247,RS_GCF_000421465.1:0.30026)56.0:0.02635)39.0:0.0204)7
4.0:0.02354)59.0:0.03292)'100.0:o__Methylococcales':0.06303,GB_GCA_018671575.1:0.43088)66.0:
0.02876)8.0:0.02332,(((GB_GCA_015484945.1:0.18046,(GB_GCA_011052255.1:0.21971,(GB_GCA_00
3233345.1:0.33413,GB_GCA_011052215.1:0.20624)33.0:0.02752)43.0:0.02945)80.0:0.06237,(((GB_
GCA_013817245.1:0.33147,GB_GCA_002432635.1:0.36319)14.0:0.01991,(GB_GCA_001735895.1:0.4
7941,GB_GCA_016191795.1:0.32214)68.0:0.03389)16.0:0.02672,((((GB_GCA_003696465.1:0.21734,(
GB_GCA_014762505.1:0.16417,(RS_GCF_000754095.2:0.26617,(GB_GCA_003555285.1:0.34654,RS_
GCF_000378965.1:0.20661)86.0:0.03873)'59.0:o__Ectothiorhodospirales':0.02143)56.0:0.02161)39.0
:0.01767,(GB_GCA_018822845.1:0.35856,GB_GCA_011330835.1:0.29567)36.0:0.02831)51.0:0.01932
,(GB_GCA_013696315.1:0.26921,GB_GCA_014075255.1:0.35871)86.0:0.05396)69.0:0.02806,(((RS_G
CF_012044895.1:0.25422,(RS_GCF_009295635.1:0.21113,(GB_GCA_002813635.1:0.40497,GB_GCA_
007127935.1:0.33659)41.0:0.01742)55.0:0.02518)'100.0:o__Nitrococcales':0.08139,(RS_GCF_900112
605.1:0.2685,RS_GCF_001614315.2:0.28676)64.0:0.04725)39.0:0.02415,(GB_GCA_018623495.1:0.24
542,GB_GCA_002281295.1:0.46877)'81.0:o__Halothiobacillales':0.05953)4.0:0.02204)2.0:0.02025)3.
0:0.0178)0.0:0.01215,(((((GB_GCA_011389995.1:0.28511,(GB_GCA_011775625.1:0.27074,GB_GCA_
018224385.1:0.28577)'88.0:o__HK1':0.04498)71.0:0.04359,(GB_GCA_002085445.1:0.43223,((GB_GC
A_003694425.1:0.26333,(GB_GCA_013697045.1:0.33451,(GB_GCA_002299125.1:0.37551,GB_GCA_
002292285.1:0.37663)25.0:0.03448)27.0:0.02425)54.0:0.03303,GB_GCA_016198885.1:0.38064)55.0:
0.03086)14.0:0.01693)17.0:0.02043,(GB_GCA_013215805.1:0.56419,RS_GCF_000024725.1:0.24732)'
99.0:o__Nitrosococcales':0.07681)7.0:0.01627,(((((GB_GCA_015490755.1:0.30402,(GB_GCA_005240
065.1:0.2001,(GB_GCA_016195665.1:0.20658,GB_GCA_903824965.1:0.33511)45.0:0.03618)17.0:0.0
2372)6.0:0.01936,((GB_GCA_015492075.1:0.2886,GB_GCA_006218035.1:0.21651)69.0:0.05202,(((G
B_GCA_016716465.1:0.23197,GB_GCA_015488555.1:0.20231)76.0:0.03173,(GB_GCA_016124415.1:
0.24745,GB_GCA_016218365.1:0.23445)66.0:0.03726)88.0:0.03055,(GB_GCA_003230195.1:0.2236,
GB_GCA_013151745.1:0.28338)22.0:0.04592)17.0:0.01941)19.0:0.0166)5.0:0.01435,(GB_GCA_0014
47805.1:0.24117,(GB_GCA_015490395.1:0.2569,GB_GCA_011375345.1:0.16026)82.0:0.08195)3.0:0.
02658)16.0:0.02437,((((GB_GCA_002450975.1:0.20424,GB_GCA_001801365.1:0.22051)67.0:0.03402
,GB_GCA_002448875.1:0.33717)20.0:0.0203,GB_GCA_003229995.1:0.30274)50.0:0.02136,GB_GCA_
015487785.1:0.25672)1.0:0.00962)0.0:0.01417,((RS_GCF_013343005.1:0.2141,RS_GCF_003967195.1
:0.22039)17.0:0.03618,(((GB_GCA_011322575.1:0.23006,GB_GCA_014762495.1:0.20021)95.0:0.037
75,(GB_GCA_013348725.1:0.18499,GB_GCA_007128895.1:0.23397)59.0:0.03937)26.0:0.02382,(GB_
GCA_016194635.1:0.36929,GB_GCA_015231435.1:0.42873)21.0:0.02012)15.0:0.01768)0.0:0.01248)
1.0:0.02053)0.0:0.01446,(((GB_GCA_012965975.1:0.28955,(GB_GCA_018812525.1:0.27299,GB_GCA

_009908845.1:0.40311)'100.0:o__Chromatiales':0.05465)76.0:0.03065,(RS_GCF_003337735.1:0.215
8,(GB_GCA_008501635.1:0.2043,GB_GCA_003645555.1:0.28486)80.0:0.03977)8.0:0.02345)4.0:0.01
764,((GB_GCA_003527885.1:0.39212,(GB_GCA_004357005.1:0.32277,(((GB_GCA_018971365.1:0.29
268,GB_GCA_003558125.1:0.28025)77.0:0.03609,(GB_GCA_009692245.1:0.45624,((GB_GCA_01300
3425.1:0.30503,((GB_GCA_013817465.1:0.2293,(GB_GCA_003567475.1:0.26052,RS_GCF_01106454
5.1:0.23618)78.0:0.03899)21.0:0.01737,(GB_GCA_002729495.1:0.38594,GB_GCA_008975185.1:0.27
958)3.0:0.02287)5.0:0.01718)1.0:0.01377,(GB_GCA_013003115.1:0.24555,GB_GCA_002868855.1:0.
22598)19.0:0.04941)22.0:0.01586)33.0:0.04091)26.0:0.02408,GB_GCA_007130745.1:0.31694)27.0:0.
02403)9.0:0.02125)1.0:0.01924,(((GB_GCA_011321775.1:0.18471,GB_GCA_905480305.1:0.28184)81
.0:0.03241,(GB_GCA_003695825.1:0.21889,(GB_GCA_013151035.1:0.24936,(GB_GCA_002699185.1:
0.16075,GB_GCA_018819175.1:0.17906)55.0:0.02681)39.0:0.02001)53.0:0.01946)84.0:0.0419,(GB_
GCA_002255365.1:0.25941,(RS_GCF_003751635.1:0.19367,GB_GCA_015487895.1:0.16932)94.0:0.0
6398)55.0:0.0427)10.0:0.01939)0.0:0.01932)0.0:0.01656)0.0:0.01757)0.0:0.02433)1.0:0.02295)0.0:0.
0173,((GB_GCA_003696645.1:0.40448,GB_GCA_002432655.1:0.33049)77.0:0.05914,((GB_GCA_0032
28915.1:0.28781,(RS_GCF_003184265.1:0.36895,(GB_GCA_002451085.1:0.23426,(GB_GCA_001785
115.1:0.25035,GB_GCA_003251795.1:0.25477)48.0:0.02745)94.0:0.03844)98.0:0.03895)'98.0:o__Aci
diferrobacterales':0.05206,((((GB_GCA_018263405.1:0.30149,RS_GCF_014651515.1:0.40884)100.0:0
.05808,(((GB_GCA_905478855.1:0.34885,GB_GCA_002456995.1:0.3887)84.0:0.04788,(GB_GCA_009
839585.1:0.56904,GB_GCA_007570945.1:0.39892)39.0:0.02373)97.0:0.04004,GB_GCA_011052285.1
:0.39458)100.0:0.03623)100.0:0.02941,(GB_GCA_011052015.1:0.26491,GB_GCA_002897635.1:0.309
63)57.0:0.04576)42.0:0.01685,GB_GCA_003972845.1:0.34504)'94.0:o__Arenicellales':0.02334)99.0:0
.08125)7.0:0.02167)0.0:0.0189,(GB_GCA_019090185.1:0.50144,(((GB_GCA_016717705.1:0.3037,((RS
_GCF_000429065.1:0.33288,(GB_GCA_016721845.1:0.26711,RS_GCF_003264855.1:0.32987)89.0:0.0
2391)86.0:0.02665,RS_GCF_004343305.1:0.2398)100.0:0.05475)100.0:0.12226,((GB_GCA_01300250
5.1:0.34718,RS_GCF_010499265.1:0.43191)100.0:0.04926,(GB_GCA_015485125.1:0.29441,(RS_GCF
_002000055.1:0.54086,GB_GCA_003233095.1:0.31262)66.0:0.04045)48.0:0.02532)86.0:0.03258)'10
0.0:o__Xanthomonadales':0.06979,((RS_GCF_000733765.1:0.29958,GB_GCA_013823855.1:0.34957)
100.0:0.09416,(RS_GCF_003151135.1:0.29113,(RS_GCF_002088235.1:0.34306,RS_GCF_000732535.1
:0.3843)55.0:0.02705)94.0:0.03568)'100.0:o__Nevskiales':0.0982)9.0:0.0259)6.0:0.02176)0.0:0.0197
6)0.0:0.01996)0.0:0.02299)3.0:0.02804)67.0:0.05018,(RS_GCF_000423825.1:0.17833,RS_GCF_00372
1225.1:0.35935)'100.0:o__Acidithiobacillales':0.14972)76.0:0.05431)'99.0:c__Gammaproteobacteria'
:0.10987,(GB_GCA_001872725.1:0.50074,(GB_GCA_015487905.1:0.34643,(GB_GCA_015489095.1:0.
28659,GB_GCA_015488125.1:0.32064)100.0:0.1036)'100.0:o__Mariprofundales':0.22543)'96.0:c__Z
etaproteobacteria':0.07361)89.0:0.04816,((RS_GCF_002109495.1:0.30706,((((GB_GCA_015228995.1:
0.36565,GB_GCA_015229045.1:0.30507)88.0:0.04463,((GB_GCA_015229005.1:0.26179,GB_GCA_01
5231925.1:0.31062)55.0:0.04796,(GB_GCA_015231965.1:0.29951,GB_GCA_015233935.1:0.2854)75.
0:0.05393)13.0:0.02663)22.0:0.02773,((GB_GCA_015231775.1:0.41515,GB_GCA_015232645.1:0.432
46)96.0:0.03532,GB_GCA_015232395.1:0.23953)73.0:0.03254)47.0:0.03401,((GB_GCA_015234045.1
:0.27153,GB_GCA_015233785.1:0.24504)66.0:0.03586,GB_GCA_015231265.1:0.26535)71.0:0.03753)
26.0:0.03438)'100.0:c__Magnetococcia;o__Magnetococcales':0.22793,((((GB_GCA_016125375.1:0.2
411,GB_GCA_002436405.1:0.31838)100.0:0.07411,(GB_GCA_018623135.1:0.31053,GB_GCA_00345
0915.1:0.45941)99.0:0.07585)82.0:0.03929,(GB_GCA_002689455.1:0.34907,GB_GCA_002422365.1:0
.6156)84.0:0.06572)'100.0:o__UBA1280':0.16444,((GB_GCA_002238905.1:0.98364,(RS_GCF_000742
475.1:0.64582,(GB_GCA_016870135.1:0.49558,RS_GCF_000469665.2:0.83862)100.0:0.21643)'95.0:o
__Holosporales':0.08265)41.0:0.09767,(((GB_GCA_002632265.1:0.42861,GB_GCA_009780035.1:0.63
069)48.0:0.08807,(((GB_GCA_003252195.1:0.25764,(GB_GCA_002410125.1:0.2493,GB_GCA_017964
705.1:0.49848)100.0:0.0826)100.0:0.06486,(GB_GCA_009779595.1:0.55824,((GB_GCA_017507765.1
:0.30573,GB_GCA_900768465.1:0.34621)39.0:0.04436,(GB_GCA_009929265.1:0.40019,GB_GCA_01

5062395.1:0.43108)100.0:0.06146)43.0:0.02414)94.0:0.04531)'97.0:o__RF32':0.05382,(((GB_GCA_9
02787665.1:0.73854,GB_GCA_016735395.1:0.89179)99.0:0.12475,(GB_GCA_003250835.1:0.28418,
GB_GCA_016936785.1:0.42757)100.0:0.13283)47.0:0.0462,GB_GCA_017938435.1:0.49299)'99.0:o__
Rs-
D84':0.16491)87.0:0.05641)31.0:0.03348,((((((GB_GCA_016794225.1:0.45956,(GB_GCA_004295055.
1:0.35789,GB_GCA_903887035.1:0.52844)90.0:0.05321)75.0:0.04485,(GB_GCA_018063245.1:0.4530
2,(((((RS_GCF_000963705.1:0.3661,(((((RS_GCF_003385955.1:0.32161,((GB_GCA_002937525.1:0.256
92,GB_GCA_002434595.1:0.277)71.0:0.0227,GB_GCA_015222795.1:0.25083)85.0:0.0273)'100.0:o__
UBA8366':0.04558,((GB_GCA_002340345.1:0.28218,GB_GCA_905480575.1:0.36929)14.0:0.03874,((
RS_GCF_014191775.1:0.22855,(GB_GCA_001657395.1:0.19712,GB_GCA_003693905.1:0.18976)83.0
:0.0187)33.0:0.01931,(RS_GCF_000686045.1:0.27286,(RS_GCF_000515255.1:0.35498,GB_GCA_0143
24045.1:0.29433)16.0:0.02498)19.0:0.02063)'100.0:o__Kiloniellales':0.04107)10.0:0.01932)8.0:0.014
57,(GB_GCA_018823745.1:0.24655,(GB_GCA_003280605.1:0.222233,bin.590:0.182939):0.0741174)
24.0:0.04152)2.0:0.01707,(((GB_GCA_011523655.1:0.38103,GB_GCA_018699755.1:0.26289)30.0:0.0
2708,GB_GCA_003230015.1:0.34375)30.0:0.02538,((((RS_GCF_000264455.2:0.33886,((((((((GB_GCA
_013389075.1:0.27656,GB_GCA_903857685.1:0.52343)62.0:0.02443,GB_GCA_004356335.1:0.31732
)50.0:0.02286,((GB_GCA_013151255.1:0.41532,(RS_GCF_900103475.1:0.38138,(RS_GCF_001854405
.1:0.47854,(GB_GCA_015234495.1:0.53071,GB_GCA_002337085.1:0.47598)77.0:0.03588)62.0:0.023
32)100.0:0.06608)'100.0:o__Caulobacterales':0.04925,(GB_GCA_002469865.1:0.41959,(((GB_GCA_0
11008935.1:0.218665,bin.422:0.103992):0.0894997,bin.364:0.123522):0.0201341,bin.118:0.109362)
:0.409491)69.0:0.0447)21.0:0.02625)32.0:0.0275,((((((GB_GCA_017353825.1:0.48017,GB_GCA_015
494185.1:0.25893)50.0:0.01841,RS_GCF_003992725.1:0.34228)24.0:0.01448,RS_GCF_000828475.1:
0.37845)100.0:0.05489,(GB_GCA_016764455.1:0.28977,(RS_GCF_000496075.1:0.25259,((((((RS_GCF
_000688515.1:0.29051,RS_GCF_001931685.1:0.37418)8.0:0.01451,((RS_GCF_003324485.1:0.27543,
GB_GCA_019192585.1:0.30104)14.0:0.02117,(GB_GCA_013911885.1:0.24017,GB_GCA_002746425.
1:0.27599)43.0:0.0342)26.0:0.0253)5.0:0.01782,(RS_GCF_003258835.1:0.1906,RS_GCF_003053845.1
:0.19244)98.0:0.03378)1.0:0.01738,(RS_GCF_000380505.1:0.26113,((RS_GCF_003258595.1:0.35085,
RS_GCF_009600605.1:0.17552)99.0:0.04157,(RS_GCF_002770725.1:0.29272,GB_GCA_012517025.1:
0.3035)63.0:0.0195)35.0:0.01554)33.0:0.02035)5.0:0.02768,(GB_GCA_002365175.1:0.29099,((RS_GC
F_014635245.1:0.24874,RS_GCF_001693515.2:0.43184)20.0:0.01746,((RS_GCF_002982075.1:0.1951
2,RS_GCF_005144885.1:0.23354)89.0:0.03417,RS_GCF_001043955.1:0.33889)56.0:0.01821)56.0:0.0
2622)100.0:0.04709)6.0:0.02112,(RS_GCF_000971275.1:0.48432,GB_GCA_011524605.1:0.37437)39.
0:0.05247)0.0:0.02415)4.0:0.02371)2.0:0.02386)1.0:0.01899,((GB_GCA_900149695.1:0.27786,RS_GC
F_000746275.1:0.2819)44.0:0.03331,RS_GCF_007474605.1:0.26866)100.0:0.06268)0.0:0.01985,(GB_
GCA_002402005.2:0.42342,(GB_GCA_003232175.1:0.26901,GB_GCA_002869085.1:0.20807)85.0:0.0
3948)7.0:0.03348)'18.0:o__Rhizobiales':0.0368,(RS_GCF_012848855.1:0.24474,(GB_GCA_902624095
.1:0.38576,GB_GCA_002715765.1:0.26823)46.0:0.02294)'81.0:o__Parvibaculales':0.03093)18.0:0.03
794)3.0:0.02104,((GB_GCA_006844605.1:0.28894,GB_GCA_903884125.1:0.30526)11.0:0.0316,GB_G
CA_009693835.1:0.29667)19.0:0.02953)5.0:0.04594,((GB_GCA_016124315.1:0.248944,bin.504:0.21
6231):0.0480356,(((RS_GCF_001281485.1:0.368979,bin.327:0.13097):0.00106032,bin.229:0.128394)
:0.550011,((GB_GCA_003577275.1:0.2339,GB_GCA_013152615.1:0.43225)100.0:0.04351,(GB_GCA_
004356215.1:0.33333,(GB_GCA_015485385.1:0.37864,RS_GCF_014647255.1:0.27111)65.0:0.02581)
96.0:0.03305)67.0:0.02666)'65.0:o__Sphingomonadales':0.02537)62.0:0.02242)3.0:0.02597,((RS_GC
F_003173035.1:0.3013,GB_GCA_003576705.1:0.26848)72.0:0.03435,(GB_GCA_002732675.1:0.3461,
GB_GCA_016865035.1:0.32944)95.0:0.03296)23.0:0.01713)1.0:0.01463,(GB_GCA_018667855.1:0.08
37023,bin.379:0.0777317):0.271468)0.0:0.01549,((GB_GCA_016869275.1:0.31688,((GB_GCA_00969
4165.1:0.235504,bin.257:0.23641):0.0694964,GB_GCA_016869515.1:0.30771)42.0:0.04714)53.0:0.0
3708,(GB_GCA_018658465.1:0.3423,GB_GCA_002348365.1:0.28938)100.0:0.03251)38.0:0.02454)5.

0:0.02504)0.0:0.01389,((GB_GCA_002717225.1:0.43444,(GB_GCA_012267605.1:0.3074,GB_GCA_00
2238685.1:0.47731)70.0:0.03016)28.0:0.02452,(GB_GCA_015490605.1:0.52105,(GB_GCA_00272136
5.1:0.21212,GB_GCA_002937585.1:0.3977)89.0:0.0497)5.0:0.02014)16.0:0.02329)2.0:0.01883,(((GB_
GCA_002239065.1:0.24139,GB_GCA_016869145.1:0.21769)81.0:0.04574,((GB_GCA_002937455.1:0.
274184,bin.241:0.18525):0.0395758,GB_GCA_012103315.1:0.315)55.0:0.0267)'99.0:o__MPNO01':0.
04655,((GB_GCA_002746255.1:0.28923,GB_GCA_016776635.1:0.26562)64.0:0.03082,GB_GCA_0093
80075.1:0.29674)65.0:0.02987)55.0:0.01841)0.0:0.01277,(((((((((GB_GCA_002709525.1:0.259572,bi
n.668:0.189105):0.0289032,bin.246:0.171637):0.000450451,bin.442:0.206232):0.00232886,bin.259:
0.181603):0.060695,((GB_GCA_001830425.1:0.23527,(GB_GCA_018660465.1:0.21036,(GB_GCA_018
662005.1:0.138085,bin.380:0.120797):0.0696855)94.0:0.02426)21.0:0.01731,(GB_GCA_003354135.1
:0.19666,GB_GCA_016776525.1:0.16411)62.0:0.03471)12.0:0.01494)13.0:0.0169,GB_GCA_01676325
5.1:0.31701)17.0:0.01625,((GB_GCA_013414705.1:0.34351,GB_GCA_009693905.1:0.34114)43.0:0.0
2918,GB_GCA_018432935.1:0.25393)34.0:0.02031)51.0:0.02174,((GB_GCA_002723345.1:0.209791,
bin.359:0.183312):0.120159,RS_GCF_000968135.1:0.2337)52.0:0.02884)59.0:0.03128,((GB_GCA_01
3373205.1:0.2982,RS_GCF_017922435.1:0.3282)78.0:0.04729,GB_GCA_009649675.1:0.28235)51.0:0
.03049)52.0:0.02309,((GB_GCA_002753155.1:0.27389,RS_GCF_001650635.1:0.2955)98.0:0.02828,RS
_GCF_014197855.1:0.36648)89.0:0.04406)'56.0:o__Rhodospirillales':0.02965)0.0:0.01766)0.0:0.0111
7)0.0:0.01654,((((RS_GCF_017311575.1:0.51334,GB_GCA_016202695.1:0.23523)3.0:0.0339,(GB_GC
A_016780605.1:0.0874021,bin.534:0.071024):0.281088)7.0:0.02451,(GB_GCA_009694185.1:0.23942
,GB_GCA_008081395.1:0.37772)16.0:0.06778)0.0:0.02514,(GB_GCA_019244535.1:0.37096,((((GB_G
CA_009694125.1:0.22889,GB_GCA_016869415.1:0.25132)63.0:0.03575,(GB_GCA_009693995.1:0.27
174,GB_GCA_016793965.1:0.23631)22.0:0.03759)6.0:0.02173,(GB_GCA_016792605.1:0.23442,(GB_
GCA_016869645.1:0.21168,GB_GCA_016869285.1:0.29882)71.0:0.02753)8.0:0.019)3.0:0.02357,GB_
GCA_016183985.1:0.42167)3.0:0.01145)0.0:0.01447)0.0:0.00955)0.0:0.01352)0.0:0.03822,(RS_GCF_
004923295.1:0.31512,GB_GCA_013003675.1:0.26548)44.0:0.0221)0.0:0.02892,((GB_GCA_00176873
5.1:0.36526,GB_GCA_018332375.1:0.26739)21.0:0.03287,GB_GCA_019192455.1:0.33906)10.0:0.023
53)0.0:0.0261,((GB_GCA_010031785.1:0.33578,(GB_GCA_903919315.1:0.22827,GB_GCA_01612495
5.1:0.24066)100.0:0.05887)'100.0:o__UBA9219':0.0603,(GB_GCA_016699305.1:0.39188,GB_GCA_00
2343265.1:0.3312)70.0:0.05162)90.0:0.04161)0.0:0.04901,(GB_GCA_013289145.1:0.47126,(GB_GCA
_007116755.1:0.37304,(GB_GCA_001767915.1:0.52886,GB_GCA_003250865.1:0.38818)62.0:0.0234
7)'89.0:o__Micavibrionales':0.03863)9.0:0.02973)1.0:0.0276)2.0:0.03389)2.0:0.02088,((GB_GCA_002
691725.1:0.34102,GB_GCA_902547925.1:0.30875)100.0:0.08806,(GB_GCA_902612965.1:0.44583,G
B_GCA_003210055.1:0.53052)96.0:0.07346)'91.0:o__Puniceispirillales':0.12957)2.0:0.02299,(((GB_G
CA_902761495.1:0.73046,(((GB_GCA_016869075.1:0.27575,RS_GCF_000742835.1:0.31428)100.0:0.
07052,GB_GCA_903861255.1:0.42399)'93.0:o__Paracaedibacterales':0.04953,GB_GCA_902795615.1
:0.80075)49.0:0.0506)33.0:0.03816,(GB_GCA_002422845.1:0.47436,GB_GCA_903930075.1:0.39505)
'81.0:o__UBA6184':0.07752)8.0:0.03115,((((GB_GCA_002257235.1:0.41257,GB_GCA_018662925.1:0
.29914)'100.0:o__16-39-
46':0.0699,(GB_GCA_002869105.1:0.52754,(GB_GCA_016869935.1:0.33965,GB_GCA_002426825.1:
0.4478)100.0:0.06695)'93.0:o__UBA7879':0.08844)61.0:0.03184,GB_GCA_001897475.1:0.42599)9.0:
0.02637,((GB_GCA_016742295.1:0.36906,((RS_GCF_002117145.1:0.32708,GB_GCA_016794245.1:0.
32538)89.0:0.03535,(GB_GCA_013911655.1:0.40001,GB_GCA_009993885.1:0.37711)75.0:0.04091)1
00.0:0.05329)'99.0:o__Caedimonadales':0.03435,(GB_GCA_905479605.1:0.24821,GB_GCA_0132876
65.1:0.30593)'100.0:o__UBA9655':0.12349)76.0:0.02622)4.0:0.02147)30.0:0.04265)1.0:0.01718,((((G
B_GCA_002422205.1:0.31756,GB_GCA_002291845.1:0.3184)100.0:0.04067,(GB_GCA_016869895.1:
0.32536,(GB_GCA_013286285.1:0.32137,GB_GCA_013288305.1:0.29443)86.0:0.03142)36.0:0.02683
)100.0:0.0542,(GB_GCA_016124905.1:0.35035,GB_GCA_017302755.1:0.26876)99.0:0.06334)99.0:0.
04971,((GB_GCA_002787615.1:0.42933,(((RS_GCF_003072485.1:1.1529,((GB_GCA_016780625.1:0.5

8073,GB_GCA_903927415.1:0.64837)69.0:0.0513,((GB_GCA_016860565.1:0.62723,(GB_GCA_01413
2315.1:0.53423,GB_GCA_903864455.1:0.8307)47.0:0.07526)52.0:0.07427,((RS_GCF_008189405.1:0.
91681,(GB_GCA_002325765.1:1.1732,GB_GCA_001730065.1:1.13438)100.0:0.30872)85.0:0.19615,G
B_GCA_905480015.1:0.81315)27.0:0.09953)34.0:0.05111)43.0:0.04711)38.0:0.04177,(((GB_GCA_01
3214525.1:0.56703,RS_GCF_003015145.1:0.69715)90.0:0.04495,(GB_GCA_002422875.1:0.49507,GB
_GCA_000970895.1:0.36079)79.0:0.04679)55.0:0.0341,GB_GCA_001897445.1:0.54543)53.0:0.02514
)56.0:0.06913,(GB_GCA_002422795.1:0.333,GB_GCA_016722515.1:0.39737)96.0:0.04361)22.0:0.02
752)3.0:0.02057,((GB_GCA_010031735.1:0.27006,GB_GCA_003531345.1:0.46824)26.0:0.06818,(GB_
GCA_903821695.1:0.29521,GB_GCA_013288565.1:0.28369)50.0:0.04308)39.0:0.06124)12.0:0.03412
)'52.0:o__Rickettsiales':0.07116)0.0:0.02615,((((GB_GCA_002701885.1:0.447,(GB_GCA_002712065.1
:0.44814,(((GB_GCA_902588615.1:0.72137,GB_GCA_902623545.1:0.76068)'100.0:o__HIMB59':0.097
44,GB_GCA_902536485.1:0.6869)32.0:0.04534,(GB_GCA_002717245.1:0.56328,(GB_GCA_90252353
5.1:0.60658,(GB_GCA_902622825.1:0.419384,bin.341:0.24997):0.348526)'100.0:o__Pelagibacterales
':0.07961)8.0:0.03233)60.0:0.05794)69.0:0.0396)42.0:0.0384,(GB_GCA_009839325.1:0.54896,(GB_G
CA_002732015.1:0.61806,GB_GCA_002171375.1:0.44212)'98.0:o__TMED109':0.05871)52.0:0.07852
)28.0:0.04304,(RS_GCF_008932295.1:0.60188,(GB_GCA_902533755.1:0.54762,GB_GCA_902632055.
1:0.56972)'92.0:o__Rhizobiales_B':0.0572)9.0:0.0727)1.0:0.03788,(GB_GCA_014323835.1:0.6725,(G
B_GCA_013216065.1:0.55997,GB_GCA_014238935.1:0.68509)'70.0:o__JABSOH01':0.07767)73.0:0.0
6834)2.0:0.02113)3.0:0.02585)10.0:0.04017)93.0:0.08876)'93.0:c__Alphaproteobacteria':0.1224)76.
0:0.05721)'92.0:p__Proteobacteria':0.08401,(GB_GCA_016936735.1:0.67675,GB_GCA_905233945.1:
0.87725)'100.0:p__UBP6;c__UBA1177':0.19112)3.0:0.03136)0.0:0.03069,(((((GB_GCA_003230795.1:
0.37608,GB_GCA_016786595.1:0.38603)'100.0:o__SZUA-
149':0.16705,((((((GB_GCA_900299205.1:0.49945,GB_GCA_017853605.1:0.3745)57.0:0.05826,(GB_G
CA_012797725.1:0.36944,GB_GCA_002343185.1:0.42936)57.0:0.06347)27.0:0.02763,GB_GCA_9039
22175.1:0.40019)51.0:0.05013,(GB_GCA_012514925.1:0.42522,GB_GCA_003696425.1:0.29993)8.0:0
.03981)15.0:0.03878,GB_GCA_009926305.1:0.45656)40.0:0.0427,GB_GCA_003694225.1:0.4102)'100
.0:o__UBA2361':0.16448)'100.0:p__Bdellovibrionota_C;c__UBA2361':0.16852,((GB_GCA_016199125
.1:0.44641,((GB_GCA_015232315.1:0.3085,GB_GCA_015231885.1:0.31554)70.0:0.04594,GB_GCA_9
02590435.1:0.50851)100.0:0.15013)'100.0:o__SAR324':0.1225,(GB_GCA_016735295.1:0.76626,(GB_
GCA_002995645.1:0.54053,GB_GCA_001783695.1:0.42098)100.0:0.11594)'100.0:o__XYD2-FULL-50-
16':0.1318)'100.0:p__SAR324;c__SAR324':0.18107)16.0:0.03774,((((GB_GCA_016715815.1:0.3974,((
GB_GCA_002376845.1:0.42268,(GB_GCA_014584465.1:0.3678,(GB_GCA_014376935.1:0.56426,((GB
_GCA_015163875.1:0.67809,GB_GCA_002453155.1:0.42842)14.0:0.01933,GB_GCA_002321895.1:0.
37069)63.0:0.05673)30.0:0.0263)65.0:0.04206)54.0:0.0369,GB_GCA_002778785.1:0.40736)'100.0:o_
_Bdellovibrionales':0.09586)'100.0:c__Bdellovibrionia':0.20476,((RS_GCF_004006665.1:0.99987,((GB
_GCA_903867855.1:0.32655,GB_GCA_011087955.1:0.37503)'100.0:o__CAIPTA01':0.19906,(GB_GCA
_002387735.1:0.42477,((GB_GCA_013286765.1:0.42485,GB_GCA_002722705.1:0.37432)42.0:0.0309
2,GB_GCA_018969905.1:0.43593)100.0:0.13355)'100.0:o__UBA1018':0.08944)'100.0:c__Bdellovibrio
nia_A':0.06132)96.0:0.06379,(RS_GCF_009208585.1:0.78723,(((GB_GCA_903844895.1:0.35229,GB_
GCA_002238945.1:0.68247)76.0:0.08699,GB_GCA_013215275.1:0.38474)84.0:0.06185,((GB_GCA_90
2766925.1:0.58002,RS_GCF_900177345.1:0.40104)56.0:0.04194,(GB_GCA_903928445.1:0.4172,GB_
GCA_018060885.1:0.45727)63.0:0.03594)40.0:0.02506)'100.0:o__Oligoflexales':0.2371)'100.0:c__Oli
goflexia':0.15811)32.0:0.03426)17.0:0.03457,((GB_GCA_009927165.1:0.259009,bin.416:0.231597):0.
483601,(GB_GCA_002343335.1:0.74098,GB_GCA_018266335.1:0.74105)73.0:0.05936)41.0:0.05493)
34.0:0.03167,GB_GCA_001799195.1:0.70077)'4.0:p__Bdellovibrionota':0.03479)4.0:0.03729,((GB_G
CA_003247575.1:0.53576,((GB_GCA_003353845.1:0.29854,GB_GCA_018262735.1:0.29744)91.0:0.0
4953,(GB_GCA_002709835.1:0.49481,(GB_GCA_004356735.1:0.31837,(GB_GCA_011055735.1:0.293
42,(GB_GCA_900696485.1:0.3046,GB_GCA_905339255.1:0.25176)56.0:0.03368)46.0:0.02824)38.0:0

.03136)85.0:0.0365)'100.0:o__UBA9160':0.12215)'100.0:c__UBA9160':0.16681,((GB_GCA_00272467
5.1:0.224457,bin.415:0.302533):0.291923,((GB_GCA_903861005.1:0.54664,GB_GCA_016717595.1:0
.38521)31.0:0.03972,((GB_GCA_016792625.1:0.41412,(GB_GCA_002296975.1:0.32842,GB_GCA_002
683315.1:0.49681)92.0:0.08451)92.0:0.07691,(GB_GCA_016927815.1:0.41026,GB_GCA_005776735.
1:0.58251)76.0:0.04472)59.0:0.03185)'100.0:o__UBA796':0.18763)'100.0:c__UBA796':0.11243)'24.0:
p__Myxococcota_A':0.0514)2.0:0.02704)0.0:0.0218)0.0:0.02362)0.0:0.02135)0.0:0.02185)3.0:0.0481
6)2.0:0.0291)5.0:0.04713)0.0:0.01621)0.0:0.02719)0.0:0.02463)1.0:0.02787)0.0:0.01994)0.0:0.02862
)0.0:0.02079)14.0:0.03449)d__Bacteria;
